# An IL-17-DUOX2 axis controls gastrointestinal colonization by *Candida albicans*

**DOI:** 10.1038/s41467-026-72174-5

**Published:** 2026-05-04

**Authors:** Pallavi Kakade, Juan F. Burgueno, Shabnam Sircaik, Nicole O. Ponde, Jinke Li, Iuliana V. Ene, Jiwoong Kim, Shen-Huan Liang, Rebecca Yunker, Ipsita Dey, Yasutada Akiba, Shipra Vaishnava, Jonathan D. Kaunitz, Sing Sing Way, Andrew Y. Koh, Sarah L. Gaffen, Maria T. Abreu, Richard J. Bennett

**Affiliations:** 1 Department of Molecular Microbiology and Immunology, Brown University, Providence, RI USA; 2 Division of Gastroenterology, Department of Medicine, Leonard M. Miller School of Medicine, University of Miami, Miami, FL USA; 3 Division of Rheumatology & Clinical Immunology, University of Pittsburgh, Pittsburgh, PA USA; 4 Institut Pasteur, Université Paris Cité, Fungal Heterogeneity Group, Paris, France; 5 O’Donnell School of Public Health, University of Texas Southwestern Medical Center, Dallas, TX USA; 6 Greater Los Angeles Veterans Affairs Healthcare System, Los Angeles, CA USA; 7 Department of Medicine, David Geffen School of Medicine at UCLA, Los Angeles, CA USA; 8 Center for Inflammation and Tolerance, Division of Infectious Disease, Cincinnati Children’s Hospital Medical Center, Cincinnati, OH USA; 9 Department of Pediatrics, University of Texas Southwestern Medical Center, Dallas, TX USA; 10 Department of Microbiology, University of Texas Southwestern Medical Center, Dallas, TX USA; 11 Harold C. Simmons Comprehensive Cancer Center, University of Texas Southwestern Medical Center, Dallas, TX USA; 12 Department of Microbiology and Immunology, Leonard M. Miller School of Medicine, University of Miami, Miami, FL USA; 13 Present Address: F. Widjaja Inflammatory Bowel Disease Institute, Cedars-Sinai, Los Angeles, CA USA

**Keywords:** Fungal host response, Fungal biology, Pathogens, Immunology

## Abstract

*Candida albicans* is a ubiquitous fungus in the human gut yet there is little understanding as to how crosstalk between the fungus and the host regulates gut colonization. Here, we performed global expression profiling on germ-free mice colonized with *C. albicans* and found that *Duox2* and *Duoxa2*, encoding a dual NADPH oxidase activity, were upregulated in the ileum and colon. Induction of *Duox2/Duoxa2* was dependent on both candidalysin toxin secreted by *C. albicans* hyphae and host IL-17 receptor signaling. IL-17A stimulation of colonoids also efficiently induced *Duox2/Duoxa2* expression together with the concomitant production of hydrogen peroxide. The IL-17-DUOX2 axis significantly impacted *C. albicans* gut commensalism; loss of IL-17 signaling increased colonization whereas loss of DUOX2 activity reduced colonization. These results reveal how a complex interplay between *C. albicans* toxin production and a host IL-17-DUOX2 axis regulates fungal gut colonization.

## Abstract (web summary)

The fungus *Candida albicans* is ubiquitous and usually harmless in the human gut, but can also cause systemic infection. Here, Kakade et al. show that fungal secretion of an immunomodulatory toxin, candidalysin, activates a host IL-17-DUOX2 axis that regulates *C. albicans* colonization of the intestine.

## Introduction

The human gastrointestinal (GI) tract is constitutively colonized with fungal cells that are acquired early in life and are lifelong members of the gut microbiome^1–3^. Despite representing a small proportion of the gut microbiome, there is increasing evidence that fungal species play pleiotropic roles during both homeostatic conditions and intestinal dysbiosis^4–7^. *Candida albicans* is one of the most abundant and clinically relevant fungal species present in the gut. This species is a pathobiont that elicits local and systemic immune responses which can help maintain GI homeostasis, yet it can also escape this niche and spread to other organs causing systemic infection^8–11^. Several reports further implicate this species in inflammatory bowel disease (IBD), including ulcerative colitis (UC)^4,11,12^. *C. albicans* grows in a wide variety of morphological forms, with cells in the yeast form deemed optimal for colonization of germ-free or antibiotic-treated hosts^13–16^. In contrast, the hyphal form promotes the colonization of hosts harboring natural bacterial loads, in part due to the secretion of the hyphal-specific toxin candidalysin that inhibits commensal bacterial growth^17^.

Microbial homeostasis in the GI tract is regulated by multi-directional interactions including those between microbes, epithelial cells, immune cells and metabolites. Studies have shown how host factors (e.g., HIF-1α), immune responses (e.g., intestinal IgA), or changes in metabolites (e.g., short-chain fatty acids) can impact fungal gut commensalism^18–21^. *C. albicans* virulence factors such as Als3, Ece1, and secreted aspartyl proteases can also elicit important host responses in oral and systemic infection models^22^ yet a detailed understanding of the crosstalk between *C. albicans* and host during gut colonization is lacking. This includes the potential for fungal manipulation of host immune responses to enable propagation in this niche, as well as the impact of *C. albicans* colonization on the gut-brain axis^23,24^.

In this study, we addressed how *C. albicans* colonization impacts the global host transcriptome in the mammalian intestinal tract. We demonstrate that *C. albicans* cells elicit distinct gene expression changes in the ileum and colon, and yet *Duox2*/*Duoxa2* (encoding a dual NADPH oxidase activity) was highly induced in both tissues in response to *C. albicans* in germ-free hosts. The hyphal-specific gene *ECE1* (encoding the toxin candidalysin) was crucial for inducing *Duox2*/*Duoxa2* in these tissues. Elevated expression of *Duox2*/*Duoxa2* and associated H_2_O_2_ production required *C. albicans* induction of the pro-inflammatory cytokine IL-17A. Notably, DUOX2 promoted *C. albicans* gut colonization whereas IL-17 signaling inhibited it, revealing intricate crosstalk between candidalysin-induced inflammation and gut commensalism. This study therefore reveals how a dynamic interplay between secreted *C. albicans* factors (such as candidalysin) and host signaling factors (including DUOX2 and IL-17) impacts fungal commensalism in the intestinal niche.

## Results

### Gastrointestinal colonization with *C. albicans* leads to a signature host response

**Fig. 1:**
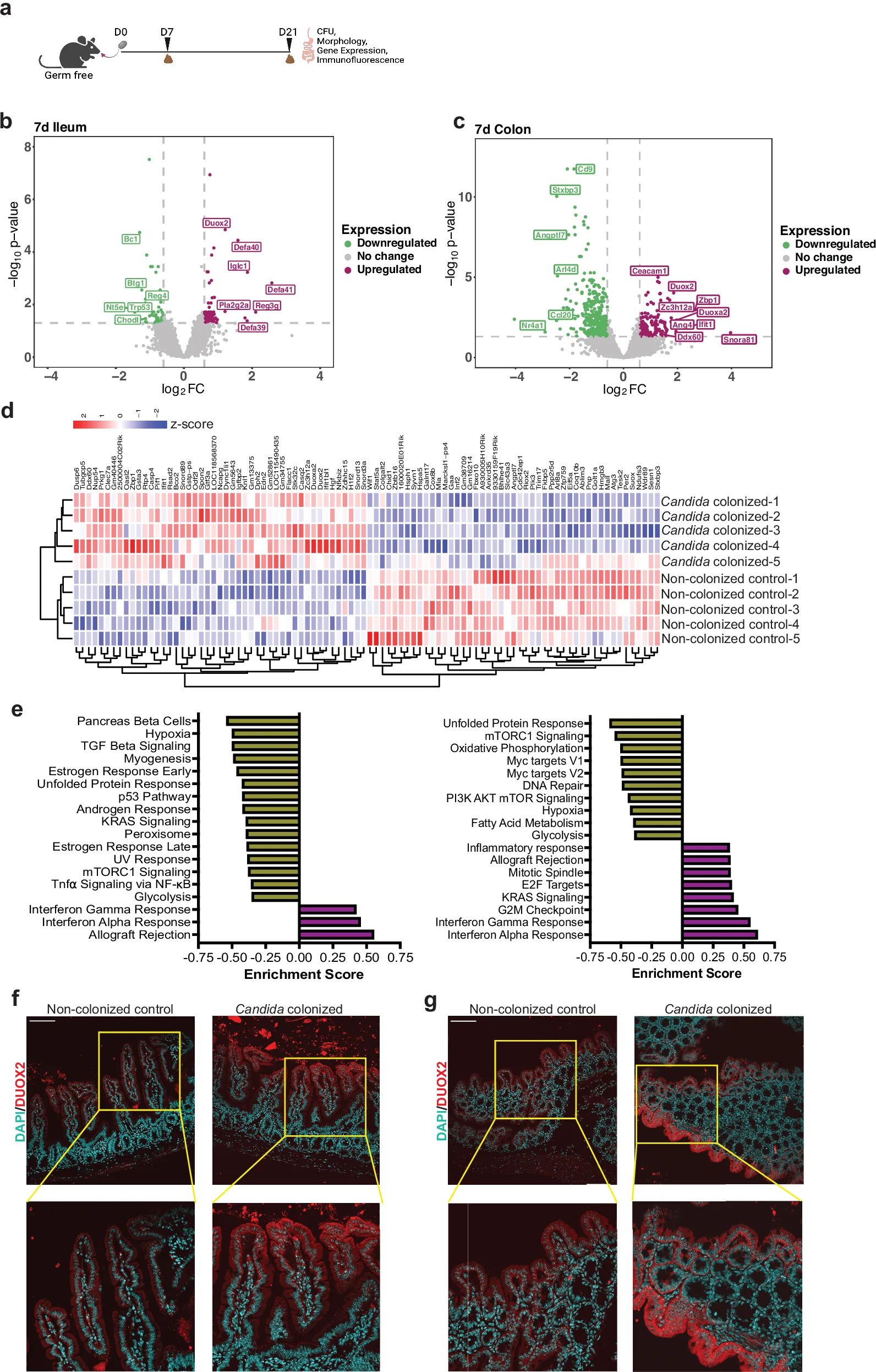
Host transcriptomic changes in response to *C. albicans* gut colonization. **a** Germ-free C57BL/6 mice were colonized with *C. albicans* WT SC5314 cells. On days 7 and 21 of colonization, mice were sacrificed to analyze fungal burdens, fungal morphology and host gene expression changes compared to a control group of non-colonized mice. Created in BioRender. Kakade, P. (2026) and published under a BioRender CC-BY publication license (https://BioRender.com/g6uabca). Volcano plots showing differentially expressed genes in ileal (**b**) and colonic (**c**) tissues of *C. albicans*-colonized mice at 7 dpi versus non-colonized mice. Genes showing expression level changes ≥1.5 and p≤0.05 are highlighted with upregulated genes shown in magenta and downregulated genes shown in green. *P*-values were attained using a Wald test corrected for multiple testing using Benjamini and Hochberg method. **d** Heat map depicting upregulated and downregulated gene clusters in the colons of *C. albicans*-colonized mice at 7 dpi versus non-colonized mice. *n* = 5 mice per group (3 females, 2 males). The z-score was used for scaling, log_2_ fold-change for magnitude and Benjamini- Hochberg test for FDR adjusted *p*-values. **e** Gene set enrichment analysis (GSEA) of significantly upregulated and downregulated genes (expression level changes ≥1.5 and p≤0.05) in day 7 ileal and colonic samples with enrichment scores. The enrichment scores were calculated using modified Kolmogorov-Smirnov test. Immunofluorescence analysis of DUOX2 in the ileum (**f**) and colon (**g**) of non-colonized and *C. albicans*-colonized mice. DUOX2 was stained with an anti-DUOX2 antibody followed by a DyLight 594-coupled secondary antibody. Epithelial nuclei were stained with DAPI. Scale bar, 100 μm. Imaging was carried out from ileum and colon tissues of all the 5 mice from each group and representative images are shown. The source data is provided as a Source data file.

*C. albicans* readily colonizes the murine GI tract in the absence of competing bacteria. To address the impact of *C. albicans* colonization on host gene expression, we utilized germ-free C57BL/6 mice colonized with the reference *C. albicans* strain SC5314 (Fig. 1a). At 7- and 21-days post inoculation (dpi), mice were sacrificed alongside a control group of germ-free mice. Fungal loads were determined from both fecal samples and GI organs and showed that *C. albicans* uniformly colonized throughout the small and large intestine and produced ~10^7^ colony forming units (CFUs)/g in fecal pellets (Supplementary Fig. 1a). The ability of *C. albicans* cells to colonize the gut is impacted by whether they adopt the yeast or filamentous state, with the yeast-locked state deemed optimal for colonization when bacterial loads are low or absent^13–16^. Fluorescent in situ hybridization (FISH) was carried out using a Cy-3 labeled pan-fungal probe and both fungal morphotypes were observed in luminal and mucosal spaces of the colon (Supplementary Fig. 1b). The proportion of hyphal cells increased from the duodenum to the colon, with ~40% of cells in the hyphal state in the duodenum which increased to ~75% of cells in the colon (Supplementary Fig. 1c).

To define the host response to *C. albicans*, distal ileum and colon tissues were collected on days 7 and 21 of colonization for bulk RNA sequencing (see “Methods”). At day 7, *C. albicans* colonization led to expression changes in 138 ileal genes and 614 colonic genes, while fewer expression changes were observed at 21 days with the altered expression of 21 genes in the ileum and 385 genes in the colon (Fig. 1b, c and Supplementary Fig. 2a, b). The top 50 genes that were the most significantly upregulated or downregulated by *C. albicans* at day 7 in colonic tissues are shown in Fig. 1d.

Most of the genes impacted by fungal colonization were distinct between the ileum and colon (Supplementary Fig. 2c–f). Defensin genes *Defa39*, *Defa40* and *Defa41*, as well as the antimicrobial peptide gene *Reg3γ*^25^, were upregulated in the ileum at 7 days, while several genes involved in immune regulation such as *Nt5e*, *Bc1*, *Btg1*, *Chodl* were downregulated in this tissue (Fig. 1b). Colonic genes upregulated by *C. albicans* at day 7 included *Ang4*, *Ddx60*, *Ifit1*, *Zbp1*, *Zc3h12a*, *Snora81* and *Ceacam1* (Fig. 1c). *Ang4* encodes a ribonuclease which serves as an antimicrobial peptide implicated in colitis and carcinogenesis^26^ while *Ddx60, Ifit1*, *Zbp1*, and *Ceacam1* drive innate immune responses. *Zc3h12a* encodes an RNase which regulates immune responses through an mRNA decay pathway^27^ while *Snora81* has been linked to cancer cell proliferation and migration^28^. Downregulated colonic genes included the immune regulatory genes *Cd9*, *Ccl20*, *Nr4a1*, *Arl4d*, *Angptl7* and *Stxbp3* (Fig. 1c). CD9 is a tetraspanin that negatively affects mucosal healing in a colitis model^29^ whereas CCL20 is a ligand for CCR6 and is involved in gut lymphoid development, with the CCL20-CCR6 axis linked to chronic IBD^30^. *Nr4a1* and *Arl4d* regulate inflammation-associated intestinal fibrosis and induction of regulatory T-cells, respectively^31,32^, while *Stxbp3* has been linked to very early onset of IBD^33^. Overall, *C. albicans* colonization impacted multiple host genes whose function has been linked to intestinal immunity and/or gut homeostasis.

The host transcriptional response to *C. albicans* at day 21 differed from that at day 7 (Supplementary Fig. 2a–f). Multiple defensin genes (e.g., *Defa3, Defa17*, and *Defa40*) were again induced in ileal samples whereas immune response genes such as *Zbtb16* were suppressed by *C. albicans* colonization. Pathway analysis of day 7 ileum and colon samples was carried out and showed that upregulated genes exhibited an enrichment for interferon alpha and gamma response pathways while downregulated genes showed an enrichment for hypoxia, unfolded protein response and mTORC1 signaling in both ileal and colonic tissues of *C. albicans*-colonized mice (Fig. 1e).

Notably, *C. albicans* significantly induced *Duox2* (~4 fold) in colonic samples at days 7 and 21, as well as in ileal samples at day 7 (~2.5 fold). *Duox2* encodes dual oxidase 2 which consists of both a peroxidase domain and a gp91phox domain and catalyzes the production of extracellular hydrogen peroxide (H_2_O_2_) in a calcium-dependent manner^34^. *Duox2* activity requires the dual oxidase maturation factor 2 encoded by *Duoxa2*. Several studies have linked these genes to GI homeostasis with increased *Duox2/Duoxa2* expression detected in individuals with IBD^35,36^ and certain *Duox2/Duoxa2* variants being predisposing factors for IBD^37^. We validated that *Duox2* and *Duoxa2* were induced by *C. albicans* in the ileum and colon at day 7 by qRT-PCR, with these genes showing 4-11-fold higher expression in colonized versus control mice (Supplementary Fig. 2g). Immunofluorescence analysis also showed increased expression of DUOX2 protein on the apical surface of epithelial cells upon *C. albicans* colonization of the ileum and colon (Fig. 1f, g and Supplementary Fig. 2h). Together, these experiments establish that *C. albicans* colonization generally brings about distinct transcriptional changes in the ileum and colon, with the dual oxidase genes *Duox2* and *Duoxa2* induced in both tissues.

### *ECE1* encoding the toxin candidalysin mediates *C. albicans* induction of *Duox2*/*Duoxa2*

**Fig. 2:**
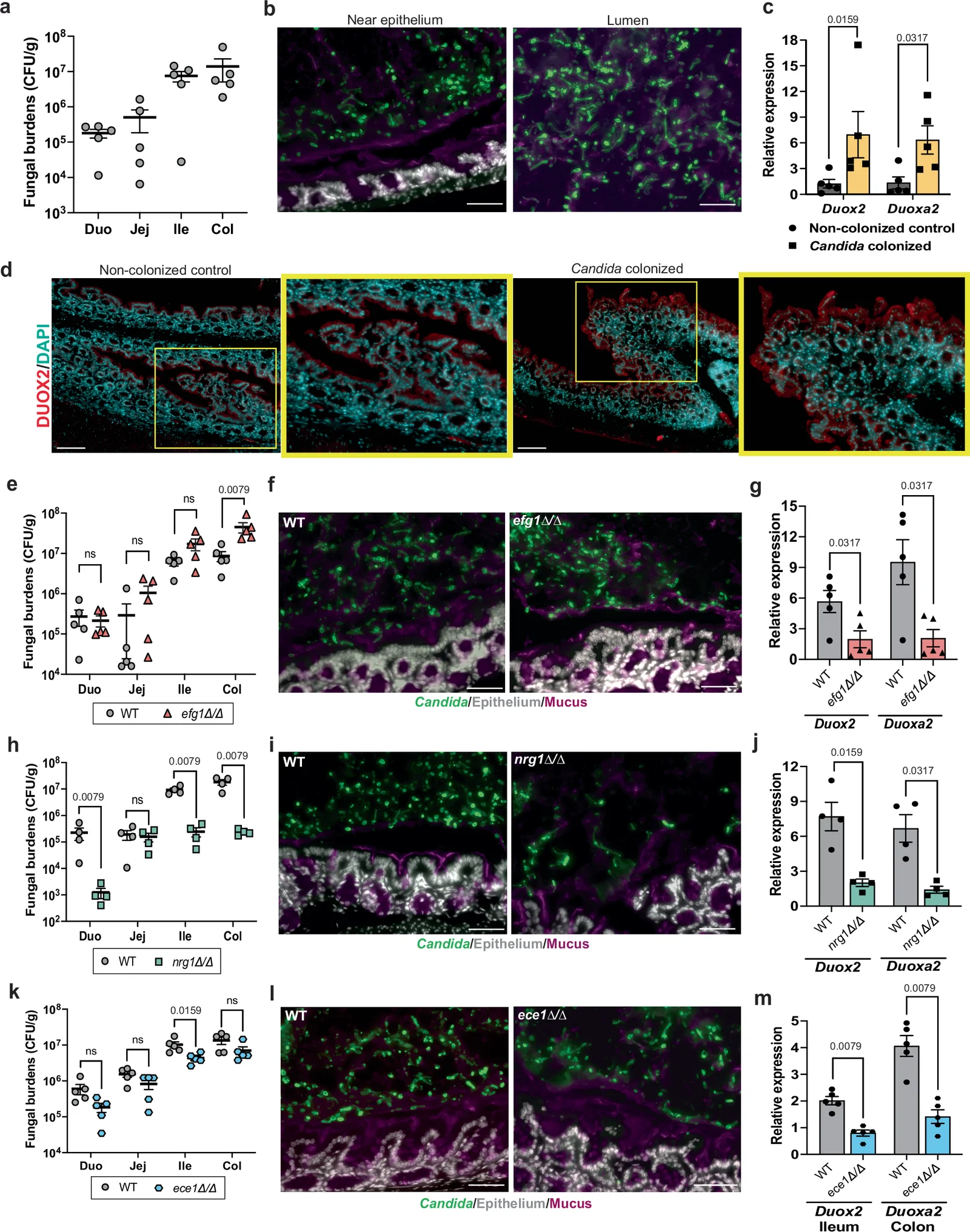
*C. albicans* morphogenesis and the hyphal-specific factor *ECE1* regulate *Duox2/Duoxa2* expression. Conventionally housed C57BL/6J mice were colonized with *C. albicans* WT SC5314 or derived strains with continued antibiotic treatment (see Supplementary Fig. 3a). **a** Fungal burdens in GI organs. **b** Immunofluorescence staining of colonic sections of *C. albicans*-colonized mice using an anti-*Candida* FITC antibody. Nuclei were stained with DAPI and mucus was stained with rhodamine-conjugated UEA-1/WGA-1. In **c**,** g**,** j**, **m** qPCR analysis was carried out to determine the relative expression of *Duox2*/*Duoxa2*. **c** *Duox2*/*Duoxa2* expression in WT colonized mice (7 days dpi) versus non-colonized controls. Error bars indicate standard error of mean (SEM) and statistical significance was determined using a two-tailed Mann-Whitney test. **d** Immunofluorescence of DUOX2 in the colon of *C. albicans*-colonized and non-colonized mice. DUOX2 was stained with an anti-DUOX2 antibody followed by a DyLight 594-coupled secondary antibody. Epithelial nuclei were stained with DAPI. **e** Antibiotic-treated hosts were colonized with WT or *efg1Δ/Δ* cells for 7 days (see Supplementary Fig. 3e) and fungal burdens in GI organs were determined. Each data point shows data from individual mouse and is presented as SEM. Statistical significance was determined using a two-tailed Mann-Whitney test. **f** Colonic tissue sections were stained with FITC-labeled anti-*C. albicans* antibody. Epithelial nuclei were stained with DAPI and mucus was stained with rhodamine-conjugated UEA-1 and WGA-1. **g** *Duox2/Duoxa2* expression in the colon of mice colonized with WT or *efg1Δ/Δ* cells in comparison to non-colonized controls. **h** Experimental plan is shown in Supplementary Fig. 4a. Fungal burdens from GI organs of WT or *nrg1Δ/Δ* colonized mice. **i** Fungal morphologies of WT and *nrg1Δ/Δ* cells detected by anti-*C. albicans* staining of colonic sections. **j** *Duox2/Duoxa2* expression in mice colonized with WT or *nrg1Δ/Δ* cells as determined by qRT-PCR. **k** Colonization levels of WT and *ece1Δ/Δ* strains in GI organs (see Supplementary Fig. 4c). **l** Morphologies of WT and *ece1Δ/Δ* cells in colonic sections after anti-*C. albicans* antibody staining. **m** *Duox2/Duoxa2* expression in the ileum and colon of mice colonized with WT or *ece1Δ/Δ* mutant cells as determined by qRT-PCR. Duo-Duodenum, Jej-Jejunum, Ile-Ileum, Col-Colon. For all experiments, *n* = 5 mice per group except for the *nrg1Δ/Δ* colonization experiment where *n* = 4 per group. For all the panels, data is presented as standard error of mean (SEM) and *p*-values were determined using a two-tailed Mann-Whitney test. Scale bar, 50 μm. The source data is provided as a Source data file.

We examined whether the genes induced by *C. albicans* in germ-free mice were also induced in conventionally housed hosts given antibiotics. Conventionally housed C57BL/6 J mice were treated with penicillin/streptomycin for 4 days prior to inoculation with *C. albicans* wild type (WT) SC5314 cells for 7 days (Supplementary Fig. 3a). Colonization levels were similar to those in germ-free hosts with ~10^7^ CFUs/g present in fecal samples and 10^5^–10^7^ CFUs/g present in GI organs (Fig. 2a and Supplementary Fig. 3b). Microscopic analysis revealed that, as in germ-free mice, the proportion of *C. albicans* cells in the hyphal form increased in descending the GI tract from the duodenum to the colon (Fig. 2b and Supplementary Fig. 3c).

*C. albicans*-induced changes in gene expression were examined by qRT-PCR and showed that colonization of antibiotic-treated hosts increased *Duox2* and *Duoxa2* expression 5–6 fold in the colon with no significant change observed in the ileum (Fig. 2c and Supplementary Fig. 3d). Immunofluorescence showed that colonization increased DUOX2 protein levels on the surface of colonic epithelial cells (Fig. 2d). We also analyzed the expression of other NADPH oxidases including *Duox1*, *Nox1* and *Nox2* but found that *C. albicans* did not significantly alter the expression of these genes (Supplementary Fig. 3e, f).

We addressed if *C. albicans* morphology impacts the host response by comparing colonization with WT SC5314 cells to that with yeast-locked *efg1Δ/Δ* cells (Supplementary Fig. 3g). Both WT and *efg1Δ/Δ* cells colonized to similar levels in antibiotic-treated hosts based on analysis of fecal pellets and GI organs, except for the colon where *efg1Δ/Δ* colonization was ~5-fold higher than the WT strain (Fig. 2e and Supplementary Fig. 3h). This is consistent with previous studies where yeast-locked cells showed higher gut fitness than WT cells when bacterial loads were low or absent^14,17,38^. *Candida*-specific antibody staining was carried out on colonic tissues and showed that WT cells adopted both yeast and hyphal forms whereas *efg1Δ/Δ* cells were almost exclusively in the yeast state (Fig. 2f and Supplementary Fig. 3i), consistent with a recent study^17^. Strikingly, yeast-locked *efg1Δ/Δ* cells showed little induction of *Duox2*/*Duoxa2* compared to WT cells, establishing that *C. albicans* hyphal formation is critical for induction of these genes (Fig. 2g).

To further address if hyphal cells contribute to *Duox2*/*Duoxa2* expression, conventionally housed antibiotic-treated mice were colonized with WT cells or *nrg1Δ/Δ* cells that are locked in the hyphal form (Supplementary Fig. 4a). Analysis showed that *nrg1Δ/Δ* cells were defective in their ability to colonize the murine host consistent with a previous study^39^. Thus, the hyphal-locked strain exhibited 10–100-fold lower colonization levels than WT cells in fecal pellets as early as 1 day after inoculation, as well as lower colonization levels in GI organs at the day 7 endpoint (Fig. 2h and Supplementary Fig. 4b). The morphology of *nrg1Δ/Δ* cells was examined in the colon and confirmed that *nrg1Δ/Δ* cells predominantly formed hyphal cells (Fig. 2i and Supplementary Fig. 4c). Hyphal-locked *nrg1Δ/Δ* cells failed to induce the expression of *Duox2*/*Duoxa2* upon colonization of the GI tract (Fig. 2j). These experiments demonstrate the importance of both morphological forms of *C. albicans* for effective colonization and induction of *Duox2/Duoxa2*.

Interestingly, *efg1Δ/Δ* cells do not express the hyphal-specific *ECE1* gene that encodes for the candidalysin toxin, while *nrg1Δ/Δ* cells are also defective in toxin production due to a decreased ability to secrete the mature toxin^40^. We directly tested whether *ECE1* is important for *C. albicans* modulation of host expression by colonizing antibiotic-treated mice with WT or *ece1Δ/Δ* cells for 7 days (Supplementary Fig. 4d). No differences were observed in the colonization levels of WT and *ece1Δ/Δ* cells in fecal samples or in GI organs except in the ileum where *ece1Δ/Δ* colonization was ~4-fold lower (Fig. 2k and Supplementary Fig. 4e). In addition, no differences were observed between WT and *ece1Δ/Δ* cells in the proportion of yeast to hyphal cells present in the GI tract (Fig. 2l, Supplementary Fig. 4f). Notably, however, *ece1Δ/Δ* cells showed marked defects in inducing *Duox2* in the ileum and *Duoxa2* in the colon (Fig. 2m). Candidalysin therefore plays a key role in inducing *Duox2*/*Duoxa2* expression in gut epithelial cells.

### *C. albicans* induction of *Duox2* involves host IL-17 signaling

To determine whether the upregulation of *Duox2*/*Duoxa2* by *C. albicans* can activate NADPH oxidase activity, we stimulated murine colonoids with 10^7^ cells/mL of heat-killed *C. albicans* yeast or hyphal forms and quantified the extracellular release of H_2_O_2_. Neither yeast nor hyphal cells increased H_2_O_2_ production compared to non-treated colonoids (Supplementary Fig. 5a). The addition of yeast cell wall polysaccharides, including curdlan, zymosan A, β-glucan and mannans also failed to induce the release of H_2_O_2_ (with these compounds pretreated with polymyxin B (PMB) to block activation by potential lipopolysaccharide (LPS) contamination; Supplementary Fig. 5b–d). These findings suggest that direct interactions between *C. albicans* and colonic cells do not induce H_2_O_2_ production.

**Fig. 3:**
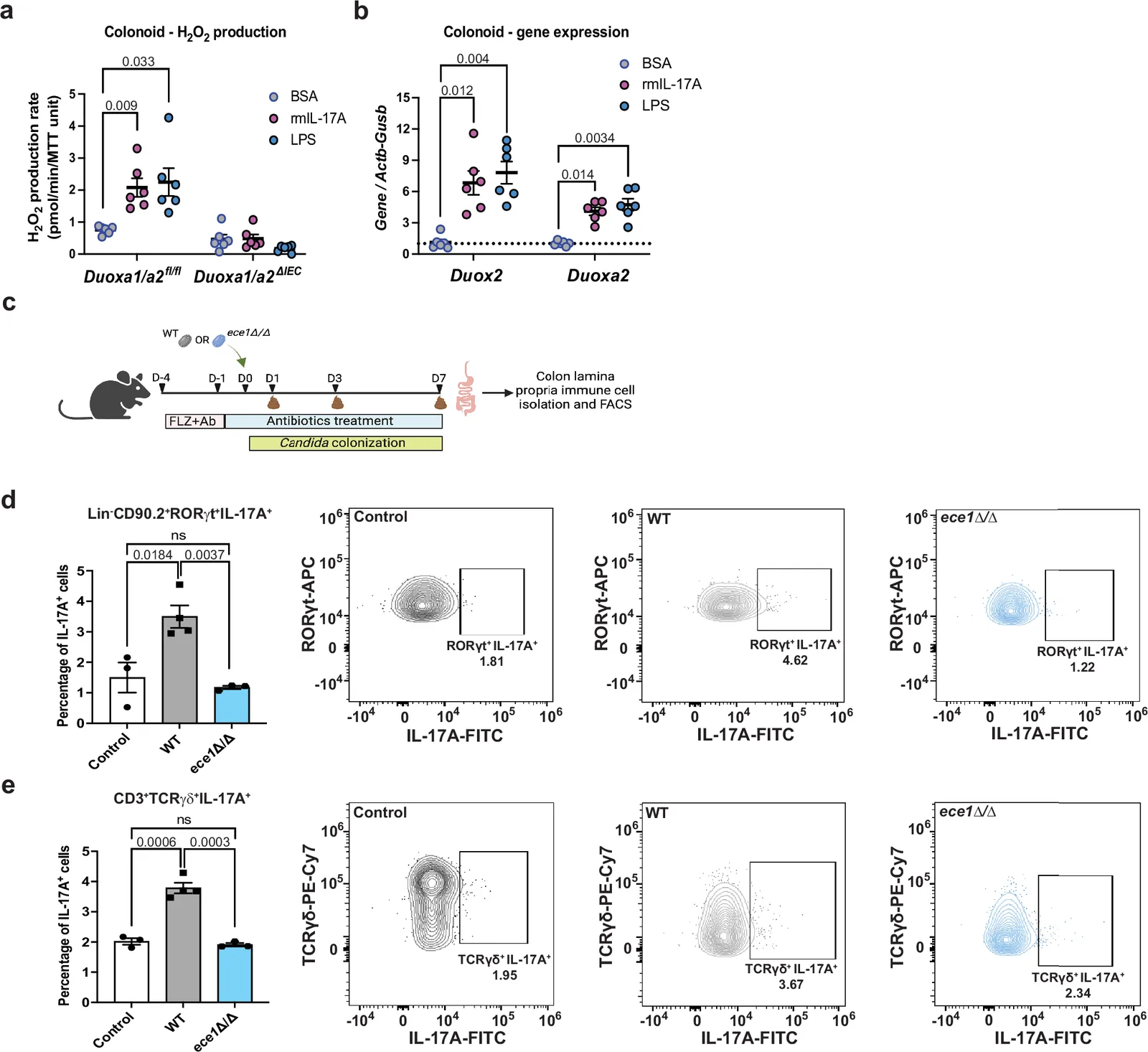
*C. albicans*-induced IL-17A promotes H_2_O_2_ production in a *Duox2/Duoxa2* dependent manner. **a**,** b** Colonoids were prepared from control mice (*Duoxa1/a2*^*fl/fl*^) or those lacking functional intestinal DUOX2 (*Duoxa1/a2*^*ΔIEC*^ mice) to evaluate H_2_O_2_ production and gene expression changes in response to recombinant murine IL-17A (rmIL-17A). Colonoids were stimulated for 24 h with 5 ng/mL of rmIL-17A or the equivalent amount of carrier BSA protein (*n* = 6 cultures). Lipopolysaccharide (LPS) was used as a positive control. **a** H_2_O_2_ production rates were normalized to MTT viability values. Data were analyzed by two-way ANOVA followed by Tukey’s post-hoc test. **b** Transcript expression levels for *Duox2*/*Duoxa2* were determined in colonoids stimulated for 24 h with BSA, rmIL-17A, or LPS (*n* = 6 cultures). Data were analyzed by means of Kruskal-Wallis test for each individual gene. **c–e** WT-C57BL/6J mice were treated with fluconazole (FLZ) and antibiotics (Ab) and colonized with either WT or *ece1Δ/Δ* cells (SC5314 background) for 7 days to quantify different subsets of IL-17A-producing immune cells (alongside non-colonized controls). On day 7 of colonization, colon tissues were subjected to immune cell isolation from lamina propria. Created in BioRender. Kakade, P. (2026) and published under a BioRender CC-BY publication license (https://BioRender.com/u0jres3). Percentage of IL-17A producing (**d**) ILC3s (gated as CD45^+^ Lin^–^ CD90.2^+^ RORγt^+^ IL-17A^+^) and (**e**) γδ T cells (gated as CD45^+^ CD3^+^ TCRγδ^+^ IL-17A^+^). Representative contour plots are shown from control, WT-colonized and *ece1Δ/Δ*-colonized mice. Detailed gating strategy is shown in Supplementary Figs. 7 and 8. *n* = 3, 4 mice per group. Data are presented as SEM, each data point represents an individual mouse and statistical significance was determined using a two-tailed unpaired t-test. ns- not significant. The source data is provided as a Source data file.

*C. albicans* candidalysin can act synergistically with IL-17A in the oral mucosa to drive host inflammation^11,41^. Given this connection, we hypothesized that *Duox2/Duoxa2* induction in gut epithelia may arise from IL-17A produced in response to *C. albicans* in this niche. To test this, colonoids were stimulated with PMB-treated murine IL-17A or a bovine serum albumin (BSA) control and IL-17A found to induce a significant increase in H_2_O_2_ production relative to BSA, at levels similar to those seen with LPS (Fig. 3a). Consistent with a model that IL-17A drives H_2_O_2_ production via DUOX2 activity, this cytokine significantly induced expression of *Duox2*/*Duoxa2* genes in WT colonoids (Fig. 3b). To establish that increased H_2_O_2_ production by IL-17A is indeed dependent on *Duox2/Duoxa2*, colonoids were prepared from mice lacking functional DUOX2 in the intestinal tract (*Duoxa1/a2*^*ΔIEC*^ mice) and shown to be non-responsive to exogenous IL-17A administration (Fig. 3a). Together, these results reveal that IL-17A increases the expression of *Duox2/Duoxa2* in colonic epithelial cells and drives DUOX2-dependent H_2_O_2_ production by these cells.

### *C. albicans* candidalysin induces host IL-17A

To investigate whether *C. albicans*-generated candidalysin drives IL-17A production during gut colonization, mice were colonized with either WT or *ece1Δ/Δ* cells for 7 days (Fig. 3c) and immune cell populations from the colonic lamina propria were evaluated. *C. albicans* colonization did not impact the percentages of ILC1 cells (CD45⁺ Lin^−^ CD90.2⁺ Tbet⁺), ILC2 cells (CD45⁺ Lin^−^ CD90.2⁺ GATA3⁺), ILC3 cells (CD45⁺ Lin^−^ CD90.2⁺ RORγt⁺), or γδ T cells (CD45⁺ CD3^+^ TCRγδ⁺)(Supplementary Figs. 6–8). In contrast, colonization with WT cells led to a significant increase in IL-17A-positive ILC3 and γδ T cell populations (Fig. 3d, e), whereas mice colonized with *ece1Δ/Δ* cells exhibited levels similar to uncolonized controls. These findings demonstrate that *C. albicans* Ece1/candidalysin induces IL-17A production in an important subset of immune cell types during GI colonization.

### IL-17 signaling promotes *Duox2* expression and suppresses *C. albicans* colonization

To further establish the dependence of *Duox2*/*Duoxa2* induction on IL-17 signaling, we assessed *C. albicans* colonization in *Il17ra*^−/−^ mice, which lack the receptor for the IL-17 cytokine family^42^. *Il17ra*^−/−^ mice initially harbored segmented filamentous bacteria (SFB) which are known inducers of IL-17^43^ and we therefore first compared *C. albicans* colonization in WT Taconic mice (WT-Tac) that naturally harbor SFBs with that in WT Jackson mice (WT-JAX) that do not harbor SFBs (and were used in the first part of this study). Mice were treated with a cocktail of antibiotics, including vancomycin to decrease SFB levels (and total bacterial loads) in WT-Tac mice as shown by qPCR (Supplementary Fig. 9a, b). Comparable fungal burdens were obtained from antibiotic-treated WT-Tac mice and WT-JAX mice (~10^7^ CFUs/g in fecal pellets; Supplementary Figs. 3b and 9c). Organs also showed similar proportions of yeast and hyphal cells except in the ileum where a smaller proportion of hyphal cells were present in WT-Tac mice than in WT-JAX mice (Supplementary Figs. 3c and 9d). Notably, *C. albicans* colonization led to high induction of *Duox2*/*Duoxa2* (25-125-fold) in the ileum of WT-Tac mice (Supplementary Fig. 9e). These data establish that *C. albicans* colonization induces gut *Duox2/Duoxa2* expression and that prior colonization with SFB may prime this response resulting in even higher levels of induction by fungal cells.

**Fig. 4:**
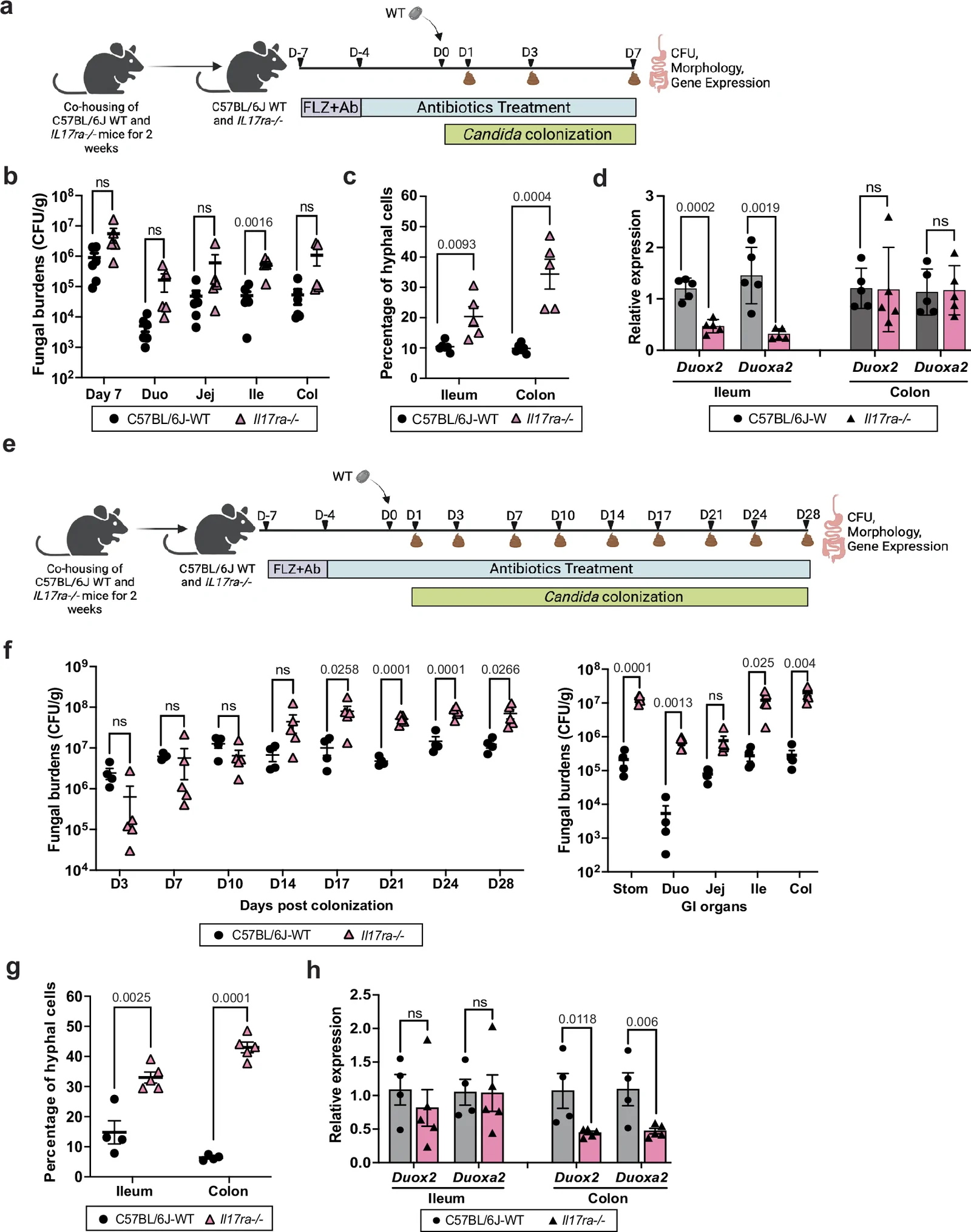
IL-17 receptor signaling regulates *Duox2* expression and suppresses *C. albicans* gut colonization. **a** WT C57BL/6J and *Il17ra*^*−/−*^ mice were co-housed for 2 weeks and then treated with fluconazole (Flz) and the antibiotics penicillin, streptomycin, and vancomycin (Ab) followed by colonization with *C. albicans* SC5314 for 7 days with antibiotic treatment (Ab) continued throughout. Created in BioRender. Kakade, P. (2026) and published under a BioRender CC-BY publication license (https://BioRender.com/p9nlk1v). **b** Fungal colonization levels were determined from fecal pellets and GI organs at 7 dpi. Duo-Duodenum, Jej-Jejunum, Ile-Ileum, Col-Colon. Data is shown as SEM, each data point represents an individual mouse and statistical significance was determined using a two-tailed unpaired t-test, ns-not significant. **c** The proportion of yeast and hyphal cells was determined from the ileum and colon of *C. albicans-*colonized WT (*n* = 5 mice per group) and *Il17ra*^*−/−*^ (*n* = 5 mice per group) mice. Paraffin embedded tissue sections were deparaffinized and stained with an anti-*Candida* antibody, epithelial nuclei were stained with DAPI and mucus was stained with rhodamine-conjugated UEA-1 and WGA-1. 500–1000 cells were counted from each tissue section (for each of 10 mice). Data is presented as SEM, each data point shows an individual mouse. Statistical significance was determined using two-tailed unpaired t-test. **d** *Duox2*/*Duoxa2* expression was determined by qRT-PCR in ileum and colon tissues of *C. albicans* SC5314-colonized mice (*n* = 5 mice per group) and *Il17ra*^*−/−*^ mice (*n* = 5 mice per group). Data is presented as relative expression with SEM; each data point represents an individual mouse. A two-tailed unpaired t-test was used to determine statistical significance; ns, not significant. **e** Experimental plan for *C. albicans* colonization of WT and *Il17ra*^*−/−*^ mice. Created in BioRender. Kakade, P. (2026) and published under a BioRender CC-BY publication license (https://BioRender.com/zwzzlyy). **f** Fungal burdens were determined from fecal samples over a period of 28 days and GI organs harvested on day 28. Data are presented as SEM with each data point showing an individual mouse and statistical significance was determined using a two-tailed unpaired t-test. **g** The proportion of yeast and hyphal morphotypes were compared from ileum and colon tissues of WT and *Il17ra*^*−/−*^ mice and presented as percent hyphal cells. Tissue sections were stained with an anti-*Candida* antibody, epithelial nuclei were stained with DAPI and mucus was stained with rhodamine-conjugated UEA-1 and WGA-1. 800–1200 cells were examined from each section (for each of 9 mice) to determine the percentage of hyphal cells. WT (*n* = 4 mice per group) and *Il17ra*^*−/−*^ (*n* = 5 mice per group) mice. Data is shown as SEM with each datapoint representing an individual mouse and statistical significance was derived using a two-tailed unpaired t-test. **h** Expression levels of *Duox2*/*Duoxa2* were evaluated from ileum and colon tissues of WT and *Il17ra*^*−/−*^ mice by qRT-PCR analysis. Data are presented as SEM, each data point shows an individual mouse and statistical significance was determined using an unpaired t-test (two-tailed). Duo, Duodenum; Jej, Jejunum; Ile, Ileum; Col, Colon. The source data is provided as a Source data file.

Next, *C. albicans* colonization was performed in antibiotic-treated WT-JAX and *Il17ra*^−/−^ mice that were co-housed for 2 weeks prior to WT SC5314 colonization (Fig. 4a). After 7 days, *Il17ra*^−/−^ mice harbored significantly higher *C. albicans* burdens in the ileum than WT mice, and colonization levels trended higher in other GI organs but did not reach significance (Fig. 4b). The *Il17ra*^−/−^ mice also contained a higher proportion of hyphal cells (both in the ileum and colon) relative to WT mice (Fig. 4c). Notably, *Duox2* and *Duoxa2* expression were significantly reduced in the ileum of *Il17ra*^−/−^ mice compared to WT mice whereas no difference was observed in expression in the colon (Fig. 4d). These data demonstrate that IL-17 signaling is required for upregulation of *Duox2*/*Duoxa2* in response to *C. albicans*, and that this signaling suppresses fungal colonization at the 7-day time point.

We next evaluated *C. albicans* colonization in WT and *Il17ra*^−^^/^^−^ mice for 4 weeks (Fig. 4e). *Il17ra*^−^^/^^−^ mice exhibited significantly increased fungal levels in fecal samples relative to those in WT mice from day 17 to day 28, and fungal levels were also higher in GI organs at the day 28 endpoint (Fig. 4f). Morphological analysis of yeast and hyphal forms in ileum and colon tissues revealed a higher percentage of hyphae in *Il17ra*^−^^/^^−^ mice than in WT mice (Fig. 4g and Supplementary Fig. 10). *Duox2*/*Duoxa2* expression was quantified in the ileum and colon and revealed a significant decrease in *Duox2*/*Duoxa2* expression in *Il17ra*^−/−^ colonic tissues relative to WT controls (Fig. 4h). Together, these findings reveal that IL-17 signaling in response to *C. albicans* increases *Duox2/Duoxa2* expression and suppresses fungal colonization and filamentation.

### *C. albicans* colonization levels are regulated by DUOX2

**Fig. 5:**
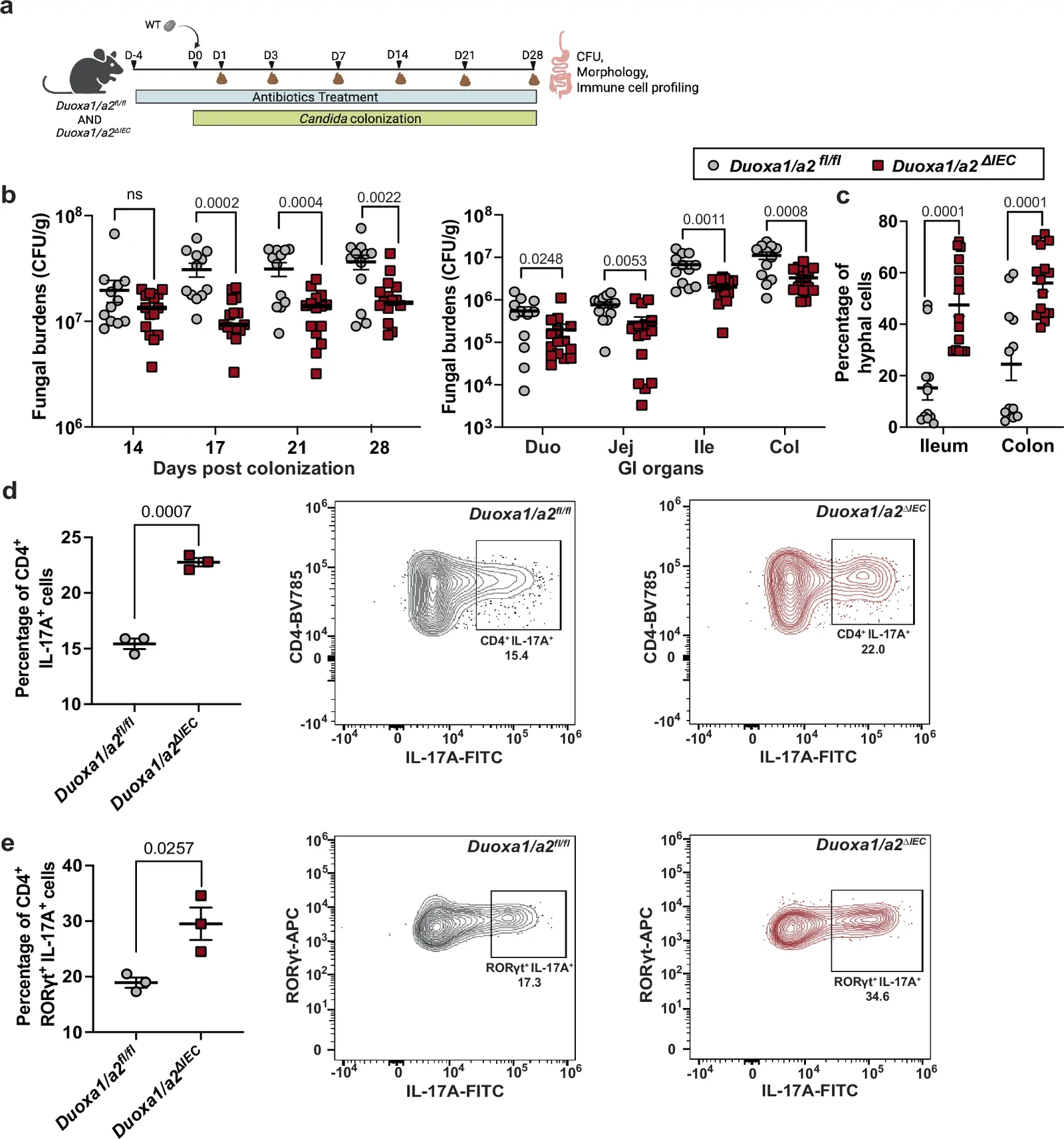
DUOX2 governs *C. albicans* colonization and filamentation through IL-17 signaling. **a** Experimental plan for *C. albicans* colonization of control *Duoxa1/a2*^*fl/fl*^ and DUOX2*-*deficient *Duoxa1/a2*^*ΔIEC*^ mice. Ab, antibiotic treatment. Created in BioRender. Kakade, P. (2026) and published under a BioRender CC-BY publication license (https://BioRender.com/84262zl). **b**. *C. albicans* colonization levels in fecal samples at the indicated time points and in GI organs 28 days post inoculation. Data is pooled from three independent experiments and error bars show SEM with a two-tailed unpaired t-test used for determining the statistical significance. For *Duoxa1/a2*^*fl/fl*^, *n* = 12 mice per group, and for *Duoxa1/a2*^*ΔIEC*^, *n* = 15 mice per group. **c** Proportion of *C. albicans* yeast/hyphal cells in the ileum and colon. Tissue sections were stained with an anti-*Candida* antibody. Epithelial nuclei were stained with DAPI and mucus was stained with rhodamine-conjugated UEA-1 and WGA-1. 800–1000 cells were examined by microscopy from each section (for each of 27 mice). Data is presented as standard error of mean (SEM) and statistical significance was determined using a two-tailed unpaired t-test. ns- not significant. Mice were given antibiotics (penicillin/streptomycin) and colonized with WT *C. albicans* cells for 28 days to quantify subsets of T cells from the lamina propria of colon tissues. Percentage of (**d**) CD4^+^ IL-17A^+^ (gated as CD45^+^ CD4^+^ IL-17A^+^) cells and (**e**) RORγt^+^ IL-17A^+^ (gated as CD45^+^ CD4^+^ RORγt^+^ IL-17A^+^) cells. Representative contour plots are shown from wild type and DUOX2-deficient mice for each cell type. Detailed gating strategy is shown in Supplementary Fig. 13. *n* = 3 mice per group. Data are presented as SEM and statistical significance was determined using a two-tailed unpaired t-test. Duo-Duodenum, Jej-Jejunum, Ile-Ileum, Col-Colon. The source data is provided as a Source data file.

To this point, our experiments indicate that *C. albicans* increases IL-17 signaling and *Duox2*/*Duoxa2* expression, and that loss of IL-17 signaling promotes fungal colonization. To directly evaluate whether DUOX2 impacts fungal commensalism we utilized mice lacking *Duoxa1*/*a2* in gut epithelial cells. These mice lack functional DUOX2 as the DUOXA2 maturation factor is essential for NADPH oxidase function^44^. Control *Duoxa1*/*a2*^*fl/fl*^ mice and mutant *Duoxa1/a2*^*ΔIEC*^ mice were treated with penicillin/streptomycin and colonized with *C. albicans* WT SC5314 cells (Supplementary Fig. 11a). No differences in fungal colonization levels were observed between *Duoxa1/a2*^*fl/fl*^ and *Duoxa1/a2*^*ΔIEC*^ mice over the first 7 days either in fecal pellets or in GI organs (Supplementary Fig. 11b). However, *Duoxa1/a2*^*ΔIEC*^ mice harbored significantly lower levels of *C. albicans* than control mice from days 17 to 28 (Fig. 5a, b). A higher proportion of hyphal cells was present in *Duoxa1/a2*^*ΔIEC*^ mice compared to *Duoxa1/a2*^*fl/fl*^ mice at 28 days (Fig. 5c and Supplementary Fig. 12a). Transepithelial translocation of fungal cells to mesenteric lymph nodes (MLNs) was minimal in both *Duoxa1/a2*^*fl/fl*^ and *Duoxa1/a2*^*ΔIEC*^ mice as determined by ITS1 PCR (Supplementary Fig. 12b).

Previous studies have shown that *C. albicans* colonization can induce hyphal-specific IgA responses in the gut^19^. To investigate whether DUOX2 influences these responses, we measured both total and *C. albicans*-specific IgA levels in the cecal contents of *Duoxa1/a2*^*fl/fl*^ and *Duoxa1/a2*^*ΔIEC*^ mice after 28 days of colonization. While total IgA levels were comparable between the two groups, *C. albicans*-specific IgA levels showed a significant increase in *Duoxa1/a2*^*ΔIEC*^ mice (Supplementary Fig. 12c).

While our experiments focused on *C. albicans* colonization of germ-free or antibiotic-treated mice, we also evaluated colonization of conventionally housed mice without antibiotic dysbiosis. *Duoxa1/a2*^*fl/fl*^ and *Duoxa1/a2*^*ΔIEC*^ mice were fed either a standard diet (SD) or a purified diet (PD), with the latter diet promoting stable *C. albicans* colonization even in the absence of antibiotics. In these models, no significant differences were observed between colonization levels in *Duoxa1/a2*^*fl/fl*^ and *Duoxa1/a2*^*ΔIEC*^ mice either in SD- or PD-fed mice over 28 days (Supplementary Fig. 17a–d). These findings indicate that, in the presence of a replete bacterial microbiome, the DUOX2 pathway does not significantly impact *C. albicans* colonization levels in the gut.

### DUOX2 tuning of gut immune responses

The role of DUOX2 in defining immune cell populations was evaluated after 7 or 28 days of *C. albicans* colonization in antibiotic-treated *Duoxa1/a2*^*fl/fl*^ and *Duoxa1/a2*^*ΔIEC*^ mice. At 7 days of colonization no differences between control and DUOX2-deficient mice were observed in lineage-negative CD90.2^+^ NKp46^+^ RORγt^+^ cells or CD90.2^+^ NKp46^−^ RORγt^+^ ILC3 cells that are innate producers of IL-17A (Supplementary Figs. 14d,e and 16) or in T cell populations involved in adaptive responses (Th1, Th2, Th17 and Treg; Supplementary Figs. 14a–c and 15). There were also no significant differences in the percentage of CD4^+^ Tbet^+^ (Th1), CD4^+^ GATA3^+^ (Th2), CD4^+^ FOXP3^+^ (Treg), CD4^+^ RORγt^+^ (Th17) or CD4^+^ RORγt^+^ FOXP3^+^ double positive cells between *Duoxa1/a2*^*fl/fl*^ and *Duoxa1/a2*^*ΔIEC*^ mice at 28 days of colonization, (Supplementary Fig. 12d–g). However, fungal colonization led to a significant increase in the proportion of CD4^+^ IL-17A^+^ cells in DUOX2-deficient mice relative to control mice (Fig. 5d). These included CD4^+^ RORγt^+^ (Th17) cells with increased IL-17A expression (Fig. 5d, e and Supplementary Figs. 12, 13). Thus, we conclude that DUOX2 suppresses IL-17A production from Th17 cells and loss of this signaling results in increased GI colonization by *C. albicans*.

## Discussion

*C. albicans* is a key component of the gut mycobiome where it elicits both local and systemic responses, but can escape this niche to cause systemic disease. The gut epithelial layer therefore represents a critical interface between fungus and host for maintaining homeostasis. Previous studies have identified several host factors that restrict *C. albicans* colonization, including hypoxia-inducible factor-1α (HIF1α) and the antimicrobial peptide LL-37^18^. Immune cells such as CX3CR1^+^ monocytes and the IL-9/mast cell axis also control *C. albicans* under both homeostatic and disease conditions^45^, while overexpression of the chitin-binding receptor FIBCD1 in gut epithelial cells limits *Candida* colonization^46^. Recent studies have further shown that the host specifically targets the hyphal form of *C. albicans*, considered the more invasive form of the species, with peptide YY produced by Paneth cells exhibiting selective activity against hyphae^47^, while secretion of IgA also selects against filamentous cells to limit intestinal damage^19,21^. These studies establish that multiple host factors impact *C. albicans* commensalism and yet a global analysis of host transcriptomic changes induced by this fungus had yet to be performed.

Here, RNA-seq was performed on the ileum and colon to define host responses to *C. albicans* in germ-free mice. Fungal colonization induced a suite of defensin genes in the ileum, including *Defa3*, *Defa17*, *Defa39*, *Defa40*, *Defa41*, as well as the antimicrobial peptide-encoding gene *Reg3γ*^25^. Host responses in the colon were largely distinct from those in the ileum and included increased expression of immune regulatory/effector genes such as *Zbp1*, *Zc3h12a*, *Ifit1*, and *Ddx60*. *Ang4*, encoding an anti-microbial peptide^48^, was also induced by *C. albicans* in the colon, as was the immunoglobulin superfamily factor *Ceacam1* which is a known sensor of pathogenic bacteria and viruses^49^. Many of these genes are expressed by gut epithelial cells upon encounter with microbial antigens and are associated with antibacterial defense, although these factors have not, to our knowledge, been previously associated with fungal commensalism.

We focused on *Duox2/Duoxa2* as both genes are upregulated in response to *C. albicans* colonization in the ileum and colon. DUOX2, together with its maturation factor DUOXA2, is considered a primordial defense system through production of H_2_O_2_ in the GI tract^50^. DUOX2 is among seven known NADPH oxidases with only two, NOX1 and DUOX2, expressed in intestinal epithelial cells. *C. albicans* colonization increased *Duox2/Duoxa2* expression on the apical surfaces of epithelial cells in both germ-free and conventionally housed hosts, while no changes in *Nox1* expression were observed. *Duox2* has also been shown to be induced by bacterial dysbiosis and in IBD, with several bacterial species, including SFB, *Citrobacter rhodentium* and *Enterobacteriaceae,* linked to increased *Duox2* expression^36,37,50–52^. The present study demonstrates that a fungal pathobiont similarly induces *Duox2/Duoxa2* expression which in turn can enhance H_2_O_2_ production.

*C. albicans* transitions between yeast and hyphal forms in response to environmental cues and secretes immunomodulatory factors such as the hyphal-specific toxin candidalysin^53–57^. We show that the yeast-hyphal transition dictates host transcriptomic responses in the gut, with yeast-locked *efg1Δ/Δ* cells, hyphal-locked *nrg1Δ/Δ* cells, and those lacking candidalysin (*ece1Δ/Δ* cells) unable to induce *Duox2/Duoxa2* expression. These results build on recent observations that connect filamentation and candidalysin to intestinal damage and induction of IL-1β, IL-17/Th17 and antibody responses, with links to patient inflammation and IBD^11,19,21,39,58^. Although a driver of inflammation, Ece1 expression can also promote *C. albicans* colonization, particularly in the presence of high bacterial loads, highlighting how this factor provides an intrinsic benefit to *C. albicans* commensalism^17^. It will now be important to define if other hyphal-specific factors also enable colonization given recent evidence that several of these factors are targets of intestinal IgA^19^.

We reveal that induction of *Duox2/Duoxa2* by *C. albicans* requires the pro-inflammatory cytokine IL-17A. Treatment of colonoids with recombinant IL-17A induced *Duox2*/*Duoxa2* and H_2_O_2_ production, while IL-17A treatment of colonoids lacking functional DUOX2 did not generate H_2_O_2_. Notably, IL-17 signaling restricted *C. albicans* gut colonization as increased fungal burdens were observed in *Il17ra*^−/−^ mice relative to control mice (post day 17 of inoculation). A previous study observed slightly elevated *C. albicans* colonization of *Il17a*^−/−^ and *Il17ra*^−/−^ mice compared to WT mice at extended time points, but reported that these differences did not reach statistical significance^39^. In contrast, we observed that fungal burdens in WT fecal pellets were ~10-fold less than those in *Il17ra*^−/−^ mice at 28 dpi, with an even bigger difference evident in intestinal organs (Fig. 4f). The difference between the previous study and the current one could be due to our use of WT SC5314 versus that of a derivative strain in which filamentation was under regulatable control.

Defects in IL-17 signaling have long been recognized as predisposing the host to oral and systemic candidiasis due to defects in neutrophil recruitment and antimicrobial peptide (AMP) production^59–65^. In line with these observations, treatment of individuals with biologics targeting the IL-17 pathway can increase the risk of oropharyngeal, esophageal, and cutaneous candidiasis due to reduced anti-*Candida* immunity^66^. In contrast, much less is known about IL-17 responses to fungi in the intestinal tract, although *C. albicans* and other mucosa-associated fungi increase the levels of Th17 cells and neutrophils in the gut^11,45,67^. Th17 cells can play contrasting roles in the gut; they can promote epithelial barrier function during homeostasis but can increase IBD during dysbiosis^68^. Our results demonstrate that IL-17 signaling also restrains *C. albicans* colonization levels in the antibiotic-treated gut.

While mice lacking IL-17 responsiveness showed increased *C. albicans* gut colonization, those lacking functional DUOX2 showed decreased colonization levels from day 17 until the experimental endpoint at day 28. Interestingly, while IL-17A was linked to induction of DUOX2, DUOX2-defective mice showed increased levels of IL-17A at day 28, indicating that DUOX2 negatively feedbacks on IL-17 signaling. Indeed, we hypothesize that the increase in IL-17A at later time points is responsible for the decreased colonization levels observed in DUOX2-defective mice.

Interestingly, Duan et al. recently showed that ER stress can promote Th17 differentiation in the gut through DUOX2-mediated production of H_2_O_2_; the latter increased the release of xanthine from epithelial cells which led to increased Th17 cell differentiation^69^. In contrast, however, we found that *C. albicans* colonization resulted in a higher proportion of IL-17A-producing Th17 (CD4^+^ RORγt^+^ IL-17A^+^) cells in DUOX2-deficient mice than in control mice. A possible explanation for this apparent contradiction comes from studies showing that reactive oxygen species (ROS), including H_2_O_2_, can inhibit the expression of RORγt and production of IL-17A in Th17 cells^70^. Our study is therefore consistent with a model in which reduced levels of H_2_O_2_ in mice lacking DUOX2 results in increased IL-17A levels, driven primarily by intestinal Th17 cells.

**Fig. 6:**
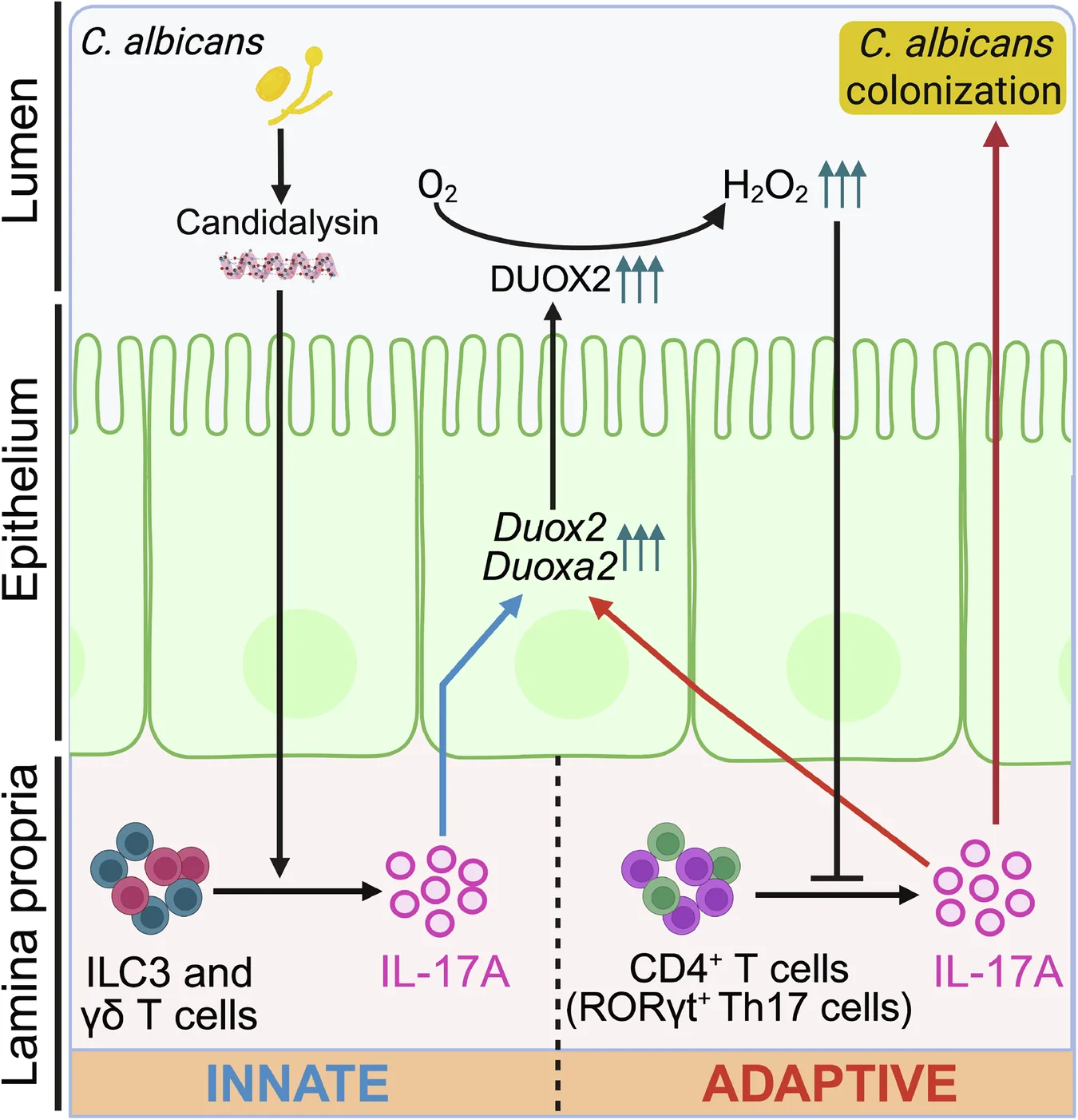
The IL-17-DUOX2 axis regulates *C. albicans* gut colonization. *C. albicans* hyphal cells secrete candidalysin toxin that induces IL-17A from innate ILC3 and γδ T cells. Increased IL-17A levels lead to elevated expression of *Duox2/Duoxa2* in gut epithelial cells. This in turn leads to increased H_2_O_2_ levels and suppression of IL-17A production in adaptive immune cell populations, including CD4^+^ RORγt^+^ Th17 cells. Overall, IL-17 signaling inhibits fungal colonization whereas DUOX2 promotes colonization, likely due to its inhibition of adaptive IL-17 signaling. Created in BioRender. Kakade, P. (2026) and published under a BioRender CC-BY publication license (https://BioRender.com/ypbdiyo).

Together, our results suggest that *C. albicans* colonization activates an IL-17-DUOX2 axis as shown in Fig. 6. At early time points (7 dpi), colonization induces IL-17A from ILC3 and γδ T cells. This induction is driven, at least in part, by the hyphal-specific toxin candidalysin. IL-17A signaling in turn increases the expression of *Duox2/Duoxa2* and elevates H_2_O_2_ levels. At later time points (>17 dpi), DUOX2 levels remain elevated which leads to a reduction in IL-17A. We note that IL-17 signaling has a bigger impact on *C. albicans* colonization than DUOX2-mediated effects; fungal colonization levels were enhanced ~5-fold by DUOX2 signaling (at day 28), whereas loss of IL-17 signaling resulted in a 10–100-fold increase in *C. albicans* GI burdens at this time point. This indicates that IL-17 activates additional, DUOX2-independent pathways that suppress fungal gut colonization.

As a part of this study, we also assessed the relative proportions of *C. albicans* yeast and hyphal cells during gut colonization given prior links between morphology and colonization fitness^14–17^. We found that the proportion of hyphal cells increased significantly in mice lacking either IL-17 signaling or functional DUOX2. Colonization levels were elevated in the former but decreased in the latter, demonstrating that changes in morphology did not correlate with overall colonization fitness. Ost et al. previously showed that *C. albicans* colonization of *Rag1*^−/−^ mice also exhibits a higher proportion of hyphal cells and linked this to the loss of hyphal-targeting IgA^19^. In contrast, we observed that *Candida*-specific IgA was higher in DUOX2-defective mice, where hyphal proportions were elevated, indicating that a simple correlation between IgA levels and hyphal formation does not always exist. Thus, multiple forces regulate the yeast-hyphal dichotomy in addition to IgA-mediated selection against hyphal forms.

In conclusion, our results establish how a complex crosstalk between the hyphal-specific toxin candidalysin, epithelial expression of DUOX2, and host IL-17 signaling can impact *C. albicans* commensal colonization of the mammalian GI tract.

We note several limitations to this study. First, in addressing the role of *C. albicans* morphology, we utilized a hyphal-locked strain (*nrg1Δ/Δ*), which colonizes poorly relative to WT or yeast-locked (*efg1Δ/Δ*) strains. We are therefore unable to ascertain whether the reduced response to the hyphal-locked strain is due to its morphology or simply a consequence of its low colonization fitness. A second limitation is that *Duoxa1/a2*^*ΔIEC*^ mice lack functional DUOX1 as well as functional DUOX2. However, *Duox1*/*Duoxa1* are expressed at very low levels in the gut, and these levels were not increased by *C. albicans* colonization. We therefore propose that *Duoxa1/a2*^*ΔIEC*^ mice are an appropriate model for evaluating intestinal DUOX2 function as previously described^51^. A third question is whether DUOX2 is acting via the increased production of H_2_O_2_ in the GI tract. This is technically challenging to address with limited tools available to accurately determine ROS levels in the host. Finally, certain differences were observed between responses to *C. albicans* in the ileum and colon, with *C. albicans*-colonized mice lacking IL-17 signaling showing a significant defect in *Duox2*/*Duoxa2* expression only in the ileum (3-4-fold down) at day 7, whereas at day 28 the IL-17-defective mice showed a significant decrease in *Duox2*/*Duoxa2* expression only in the colon (2-fold down). These differences could indicate that different mechanisms operate in the ileum versus the colon in response to *C. albicans* and additional experiments are necessary to address this possibility.

## Methods

All the animal studies were performed according to approved protocols by the Institutional Animal Care and Use Committee (IACUC) of each institution in the US. The IACUC approval number for studies conducted at Brown University is 24-09-0007.

### Materials

All the reagents used in this study are listed in Supplementary Table 4.

### Strains

*C. albicans* SC5314 and derived strains are listed in Supplementary Table 1 and were grown on YPD medium (1% yeast extract, 2% peptone, 2% dextrose) at 30 °C as standard.

### Mice

Wild type C57BL/6J mice were purchased from Jackson Laboratories (Strain#000664) and C57BL/6NTac from Taconic Biosciences. Germ-free mice were obtained from the gnotobiotic facility at Brown University and experiments involving germ-free mice were carried out in this facility. Epithelial specific knockouts of *Duox2* (*Duoxa1/a2*^*ΔIEC*^) were generated by crossing the *Duoxa1/2* -floxed (*Duoxa1*/*a2*^*fl/fl*^) mice (generated at the Mouse Biology Program at UCDavis under the guidance of Dr Kaunitz [University of California, Los Angeles]) with villin-cre (Tg[Vil1-cre]997Gum) mice purchased from Jackson Laboratory. The expression of DUOX1 in the gut is remarkably low and hence *Duoxa1/a2*^*ΔIEC*^ mice are commonly accepted as a model to explore the role of intestinal DUOX2^51^. Mouse colonies of *Duoxa1*/*a2*^*fl/fl*^ and *Duoxa1/a2*^*ΔIEC*^ were established at the Brown University SPF facility and mice were genotyped by carrying out multiplexed, touchdown PCR using primers 16775, 16776, and oIMR9074. For each experiment involving these mice, littermate controls were used. Experiments involving *Il17ra*^*−/−*^ mice were carried out in the Gaffen lab. *Il17ra*^*−/−*^ mice were a gift from Amgen. All the mouse strains used in this study are listed in Supplementary Table 2.

### Germ-free gut colonization model

11–12 weeks old germ-free C57BL/6 males or females (Brown University facility) were colonized with *C. albicans* SC5314 by inclusion of 10^7^ cells in 500 mL of drinking water for 3 days in gnotobiotic chambers. *C. albicans* cells were grown overnight in YPD at 30 °C on a rotary shaker and cultures were then diluted 1:50 in 5 mL YPD and grown for additional 4 h at 30 °C on a rotary shaker. Cells were then washed with sterile water for 3 times, resuspended in sterile water and enumerated using a hemocytometer. A group of control non-colonized mice was housed in a separate gnotobiotic chamber. On day 3, water containing *C. albicans* cells was replaced with sterile water and colonization was continued for 21 days. On days 7 and 21 of colonization, fecal samples were collected to analyze fungal burdens and mice were sacrificed to harvest GI organs.

### Antibiotic-treated colonization model

10–12 weeks old C57BL/6J female mice were purchased from Jackson laboratories and were allowed to acclimate for 4 days and given free access to food and water. 8–10 weeks old *Il17ra*^*−/−*^ and WT C57BL/6J female mice were used for colonization experiments. To establish GI colonization, mice were fed a standard chow (Labdiet #5010) and the drinking water was supplemented with antibiotics (1.5 mg/mL penicillin, 2 mg/mL streptomycin) or (1.5 mg/mL penicillin, 2 mg/mL streptomycin, 0.25 mg/mL vancomycin) and 2.5% glucose for 4 days prior to colonization. Mice from the same experimental group were co-housed throughout the experiment and antibiotics containing water was changed every 3–4 days. *C. albicans* strains were grown overnight in YPD at 30 °C on a rotary shaker and cultures were then diluted 1:50 in 5 mL YPD and grown for additional 4 h at 30 °C on a rotary shaker. Cells were washed with sterile water 3 times, resuspended in sterile water and enumerated using a hemocytometer. For inoculation, 10^7 ^*C. albicans* cells were added to 500 mL of drinking water containing antibiotics and 2.5% glucose. This water was replaced with water containing only antibiotics and 2.5% glucose after 3 days and colonization continued for 7, 21, or 28 days depending on the experiment. Fecal pellets were collected at different time points to assess fungal burdens and homogenized in PBS solution supplemented with antibiotics (500 μg/mL penicillin, 500 μg/mL ampicillin, 250 μg/mL streptomycin, 225 μg/mL kanamycin, 125 μg/mL chloramphenicol, and 125 μg/mL doxycycline) and plated on YPD plates. At the end of the experiment, fungal burdens were determined in GI organs by homogenizing organs in PBS supplemented with antibiotics and plating on YPD plates that were incubated at 30 °C for 2 days.

### Analysis of *C. albicans* morphology in the murine gut

To assess yeast and hyphal morphotypes of *C. albicans*, GI sections were imaged by fluorescence in situ hybridization (FISH) as previously described^14^. One-to-two cm pieces of duodenum, jejunum, ileum, and colon were fixed in methacarn (American Master Tech Scientific) immediately after harvesting and stored at room temperature. After 24-48 h, the tissues were washed twice with 70% ethanol and embedded in paraffin blocks. Ten micrometer sections were deparaffinized, and a previously described protocol was followed for staining^14^. *Candida* cells were stained with a Cy3-labeled PAN fungal 28S ribosomal RNA (rRNA) probe, epithelial cells were stained with 4,6-diamidino-2-phenylindole (DAPI, Molecular Probes, Invitrogen), and the mucosal layer was stained with fluorescein-labeled WGA-1 and UEA-1 (Vector Laboratories). Tissue imaging was carried out using ileum and colonic tissue sections, and images were captured using a Zeiss Axio Observer microscope. Eight to ten Z-stacks were merged to generate final images.

To evaluate *Candida* morphology in the GI tract, 10 μm tissue sections were deparaffinized, blocked with PBS plus 5% FBS for 30 min at 22 °C, and incubated with an anti-*Candida* antibody coupled to fluorescein isothiocyanate (FITC) (1:500 dilution; ThermoFisher Scientific) overnight at 4 °C. This was followed by three washes with PBS at 22 °C and staining of the epithelial nuclei with DAPI. Cell counting was carried out using a Zeiss Axio Observer microscope. 500–1000 *Candida* cells per section were assessed for morphology and proportion of yeast and hyphal morphotypes is presented as percentage.

### Immunofluorescence analysis

For the detection of DUOX2, ileum and colonic tissues were fixed in methacarn for 24–48 h and then embedded in paraffin. Ten-micrometer sections were prepared from paraffin blocks using a microtome and deparaffinized with sequential treatment of xylenes, ethanol, and PBS. Citrate buffer in combination with boiling was used for antigen retrieval and slides were blocked with 1% bovine serum albumin (BSA) prepared in PBS. Slides were stained with an anti-DUOX2 antibody (1:250; Novus Biologicals, NB110-61576) overnight at 4 °C in the dark followed by 3 washes with PBS for 10 min each and staining with secondary antibody coupled with DyLight 594 (ThermoFischer, SA5-10040). Epithelial nuclei were stained with DAPI and tissue sections were imaged using a Zeiss Axio Observer microscope. Eight to ten Z-stacks were merged to generate final images.

### RNA isolation and sequencing

Distal ileum and colon tissues were collected from control and *C. albicans*-colonized germ-free mice and stored in RNA-later solution at −80 °C until RNA extraction. 30–40 mm piece of each tissue were subjected to total RNA isolation using a PureLink RNA isolation kit. Organs were homogenized in lysis buffer using a homogenizer prior to following the kit protocol. An additional DNaseI treatment was carried out on column eluted RNA which was then checked for any genomic DNA remnants by PCR. RNA quality was determined by running RNA samples on Bioanalyzer and RNA samples with RIN value ≥7 were used to prepare libraries. RNA concentration was determined by Qubit and 500 ng of total RNA was used to prepare libraries using a 3’ end Quant-seq preparation kit (Lexogen). Concentration of each library was determined by qRT-PCR and pooled together in an equimolar concentration for sequencing on an Illumina Hi-Seq 4000.

Trim Galore (https://www.bioinformatics.babraham.ac.uk/projects/trim_galore/) was used for quality and adapter trimming. The mouse reference genome sequence and gene annotation data, mm39, were downloaded from UCSC Genome Browser and NCBI RefSeq genome database. The quality of RNA-sequencing data was determined by mapping reads onto mouse transcript and ribosomal RNA sequences using Burrows-Wheeler Aligner (BWA, v0.7.17)^71^. STAR (2.7.10b)^72^ was employed to align the reads onto the mouse genome, SAMtools (v1.16.1)^72^ was employed to sort the alignments, and HTSeq Python package^73^ was employed to count reads per gene. DESeq2 R Bioconductor package^74,75^ was used to normalize read counts and identify differentially expressed (DE) genes. The enriched pathways were identified using GSEA software (v4.3.3)^76^. Volcano plots and heat maps were generated in R-studio using required packages. Venn diagrams were created using Draw Venn Diagram tool (https://bioinformatics.psb.ugent.be/webtools/Venn/).

### Organoid culture and stimulation

Colonic epithelial cells were isolated from mice in steady state conditions by chelation in 20 mM EDTA in Hank’s balanced salt solution (HBSS) for 1 h at 22 °C, followed by gentle shaking. Isolated colonic epithelial cells were mixed with ice-cold Cultrex reduced growth factor basement membrane type R1 (R&D Systems) and cultured for 5 days in 50% conditioned medium containing wnt3a, R-spondin-3, noggin, and 20% fetal bovine serum supplemented with Chir99021 (5 µM), Thiazovivin (2.5 µM), and Primocin (100 µg/mL). On day 4, colonoids were challenged with sonicates of *C. albicans* SC5314 in yeast and hyphal forms (10^7^ cells/mL); curdlan from *Alcaligenes faecalis* (100 µg/mL in DMSO; InvivoGen); mannan (250 µg/mL in 1:1 PBS/DMSO solution; Millipore-Sigma), zymosan A (250 µg/mL in 1:1 PBS/DMSO solution; Millipore-Sigma), β-glucan from *Saccharomyces cerevisiae* (100 µg/mL in 1:1 PBS/DMSO solution; Millipore-Sigma); recombinant mouse IL-17A (5 ng/mL; R&D Systems); or the appropriate vehicles and carrier proteins for 24 h. All ligands and cytokines were preincubated with polymyxin B (PMB; 25 µg/mL; Millipore-Sigma) for 30 min at 37 °C to prevent activation by lipopolysaccharide (LPS) contamination. Ultrapure LPS (1 µg/mL; InvivoGen) was used as a positive control for the induction of *Duox2*.

### Determination of H_2_O_2_ production

The kinetic release of extracellular H_2_O_2_ by colonic epithelial cells was measured via the horseradish peroxidase-mediated oxidation of Amplex red. Colonoids seeded in 96 well plates were incubated in Dulbecco’s PBS solution containing Ca^2+^, Mg^2+^, 0.1 U/mL horseradish peroxidase, and 30 µM Amplex red (Biotium) with modifications^52^. Fluorescence was read at 40–60 s intervals for 15 min at 37 °C (Ex 530 nm/Em 590 nm) in a Synergy H1 fluorometer (BioTek). Following measurement of H_2_O_2_, cellular viability was assessed by incubating colonoids in 4 mM MTT (Cayman Chemical) solution in DMEM/F12 medium for 1 h at 37 °C. H_2_O_2_ production data were normalized to MTT viability values. All conditions were assayed in triplicate.

### Quantitative PCR analysis

For isolation of RNA, colonoids cultured in 96-well plates lysed in TRIzol underwent the phenol-chloroform extraction method. One hundred nanograms of RNA were retrotranscribed into cDNA by means of the PrimeScript RT reagent Kit (Takara Bio Inc.), followed by amplification using SYBR Premix Ex Taq (Takara) on a LightCycler 480 II instrument (Roche Applied Science). The primers used are shown in Supplementary Table 3 (*Duox2* qRT_F2, *Duox2* qRT_R2, *Duoxa2* qRT_F2, *Duoxa2* qRT_R2). A melting curve analysis was consistently performed for each reaction to verify the specificity of the amplification products. mRNA expression levels were calculated using the ΔΔCt method and normalized to the geometric mean of the housekeeping genes *Actb* and *Gusb*.

For qRT-PCR analysis of host genes from ileum and colonic tissues, the RNA extraction protocol as mentioned above was followed. One microgram of total RNA was converted to cDNA using the iScript kit (Bio-Rad). cDNA was diluted 2-fold and 1 μL was used for each PCR reaction with the Biorad qRT-PCR mix using a CFX Maestro (Bio-rad) machine. Primers used for the expression analysis of different host genes are listed in Supplementary Table 3 (*Duox2* qRT_F1, *Duox2* qRT_R1, *Duoxa2* qRT_F1, *Duoxa2* qRT_R1). Transcript levels were calculated using the ΔΔCt method and normalized to housekeeping gene *Rps29*.

### Preparation of lamina propria lymphocytes from colon

Lamina propria lymphocytes were isolated as described^77^. In short, mice were euthanized using isoflurane followed by cervical dislocation. Colonic tissue was harvested, contents were cleaned with ice-cold PBS and cut longitudinally first and then into 5-6 pieces and thoroughly washed with ice-cold HBSS. Colonic epithelium was removed from the underlying tissue in a stepwise manner, first by incubation for 10 min at 37 °C in HBSS (with 4.17 mM NaHCO_3_ and 3% FCS) with 1 mM DTT, 30 mM EDTA, followed by vigorous shaking. Tissue pieces were incubated in HBSS (with 4.17 mM NaHCO_3_ and 3% FCS) with 30 mM EDTA for 10 min at 37 °C. Remaining tissues were digested with Collagenase I (Sigma- Aldrich) and DNase I (Sigma- Aldrich) in RPMI complete media for 1 h at 37 °C. Cells were filtered through 70 μm cell strainers, re-suspended in RPMI complete media (3% FBS) and applied onto a 40%:80% Percoll gradient (GE Healthcare, Pittsburgh, Pennsylvania). Lamina propria lymphocytes were found at the interface of 40%:80% fractions in the Percoll gradient and collected cells were further stained for surface and intracellular markers.

### Antibody staining and flow cytometry analysis

Cells obtained from colon LP preparations were incubated for 3 h with 1x Cell Stimulation cocktail and 1x Protein Transport Inhibitor (eBioscience) at 37 °C with 5% CO_2_. Surface antigen staining was carried out with fluorescently labeled antibodies for 30 min at 22 °C. Antibodies used to stain surface markers included CD4 Antibody (BioLegend, 100551), CD45 Monoclonal Antibody (eBioscience, 64-9459-42), CD45 Antibody (BioLegend, 147716), anti-mouse CD90.2 (Thy-1.2) Antibody (BioLegend, 140319), CD335 (NKp46) Monoclonal Antibody (ThermoFisher, 12-3351-80), CD3e Monoclonal Antibody (Invitrogen, 364-0031-82), anti-mouse TCR γ/δ Antibody (BioLegend, 118123) and Biotin anti-mouse Lineage Panel (BioLegend, 133307). The lineage cocktail included biotinylated anti-CD3ε, anti-Ly-6G/Ly-6C, anti-CD11b, anti-CD45R/B220 and anti-Ter-119 antibodies. After surface staining, cells were re-suspended in Fixation/Permeabilization solution (eBioscience Foxp3 Staining Buffer Set) overnight at 4 °C, followed by intracellular cytokine and transcription factor staining using antibodies as per the manufacturer’s protocol. Antibodies used for the intracellular staining comprised of Anti-T-bet Antibody (BioLegend, 644819), Gata-3 Monoclonal Antibody (eBioscience, 46-9966-41), ROR gamma (t) Monoclonal Antibody (eBioscience, 17-6981-80), FOXP3 Monoclonal Antibody (eBioscience, 15-5773-80), and IL-17A Monoclonal Antibody (Thermo Scientific, 53-7177-81). All the antibodies used for the staining of surface as well as intracellular molecules were used at a dilution of 1:100 in staining buffer. Cells were gated on Cytek Aurora and flow cytometry data were analyzed using FlowJo V10.10.0.

### Estimation of total IgA from cecal contents

The protocol of Ost et al. was used to determine total IgA levels in cecal supernatants^19^. Briefly, SC5314 cells were grown overnight in YPD at 30 °C and then diluted the next day to 0.5 OD/mL in RPMI supplemented with 10% serum for 90 min. Cells were washed twice with PBS and resuspended in PBS containing 1% BSA and 0.01% sodium azide. Twenty-five microliters of cell suspensions were mixed with 25 µl of cecal supernatants. The antibody staining steps described in Ost et al. were followed with IgA binding to fungal cells assessed by flow cytometry.

### Statistical analysis and data reproducibility

All data analysis and plots were performed using Prism10 (GraphPad Software, Inc.) and compared using Unpaired t-test (Two-tailed), Friedman test for matched samples, Kruskal-Wallis test, or two-way ANOVA, as indicated. Results are presented with either standard deviation (SD) or standard error of mean (SEM). *P* values are reported for each analysis and comparison. All the experiments were repeated at least twice.

### Reporting summary

Further information on research design is available in the Nature Portfolio Reporting Summary linked to this article.

## Supplementary information

Supplementary Information

Reporting Summary

Transparent Peer Review file

## Source data

Source Data

## Data Availability

Raw data and processed data files for RNA-seq have been deposited at NCBI with GEO accession numbers GSE274260 and PRJNA1145473, respectively. These data files are available at https://www.ncbi.nlm.nih.gov/geo/query/acc.cgi?acc=GSE274260. Source data are provided with this paper.
