# Supplementary material for: An IL-17-DUOX2 axis controls gastrointestinal colonization by Candida albicans
Source: Nat Commun. 2026 May 4;17:6013. doi: 10.1038/s41467-026-72174-5 (PMC13346697; doi:10.1038/s41467-026-72174-5)
Supplement: Supplementary file 1 — Supplementary Information [file 41467_2026_72174_MOESM1_ESM.pdf]

Supplementary Information for-

An IL-17-DUOX2 axis controls gastrointestinal colonization by *Candida albicans*

Pallavi Kakade[1], Juan F. Burgueno[2], Shabnam Sircaik[1], Nicole O. Ponde[3], Jinke Li[1], Iuliana V. Ene[4], Jiwoong Kim[5], Shen-Huan Liang[1], Rebecca Yunker[1], Ipsita Dey[3], Yasutada Akiba[6,7], Shipra Vaishnava[1], Jonathan D. Kaunitz[6,8], Sing Sing Way[9], Andrew Y. Koh[10,11,12], Sarah L. Gaffen[3], Maria T. Abreu[2,13], and Richard J. Bennett*[1]

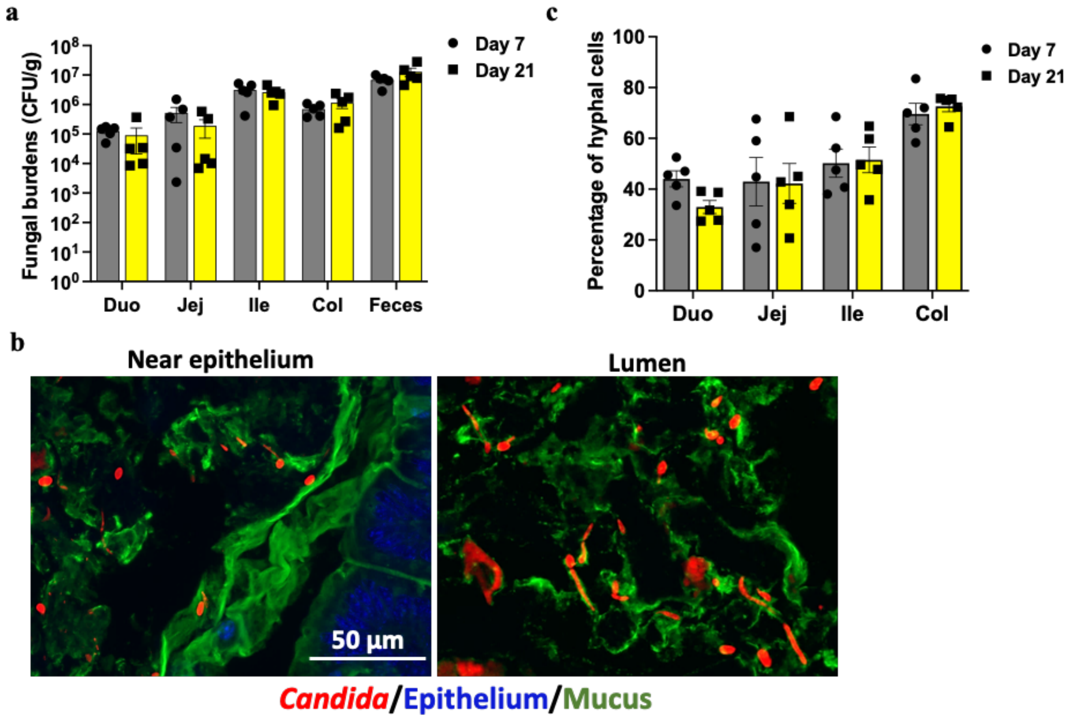

Supplementary Figure 1. ***C. albicans*** **gut colonization of germ-free mice.**

a. Fungal colonization levels following introduction of *C. albicans* SC5314 cells into C57BL6 germ-free hosts were determined by CFU analysis from fecal pellets or from different parts of the small intestine or colon collected on days 7 and 21 of colonization. n = 5 mice (3 females, 2 males) per group. **b.** Fluorescence in situ hybridization (FISH) was carried out to visualize *C. albicans* yeast and hyphal cells in colonic sections. *Candida* cells were stained with a Cy-3 labelled PAN-fungal probe, epithelial nuclei were stained with DAPI and mucus was stained with fluorescein-conjugated UEA-1/WGA-1. Scale bar, 50 µm. n = 5 mice per group. **c.** The percentage of *C. albicans* yeast and hyphal morphotypes were determined from different parts of the small intestine and colon sampled on days 7 and 21 of colonization. 500-1000 cells were counted from each tissue (in each of 10 mice). Error bars represent SEM. Duo-Duodenum, Jej-Jejunum, Ile-Ileum, Col-Colon. The source data is provided as a Source Data file.

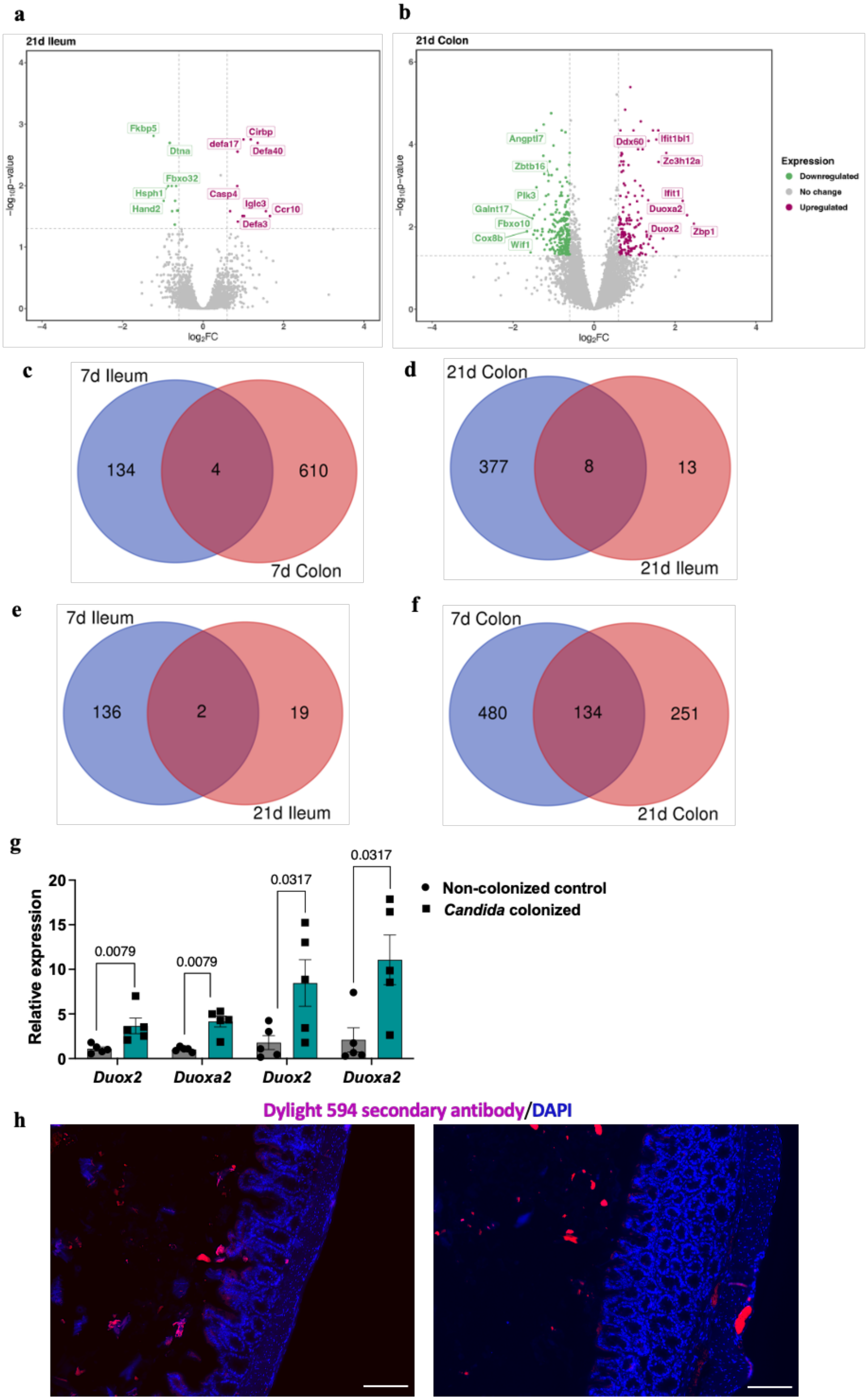
a
21d Ileum
Fkbp5
Dtna
Fbxo32
Hsph1
Hand2
Cirbp
defa17
Defa40
Casp4
Iglc3
Ccr10
Defa3
log2FC
−log10p-value
b
21d Colon
Angptl7
Zbtb16
Plk3
Galnt17
Fbxo10
Cox8b
Wif1
Ddx60
Ifit1bl1
Zc3h12a
Ifit1
Duoxa2
Duox2
Zbp1
Expression
Downregulated
No change
Upregulated
c
7d Ileum
134
4
610
7d Colon
d
21d Colon
377
8
13
21d Ileum
e
7d Ileum
136
2
19
21d Ileum
f
7d Colon
480
134
251
21d Colon
g
Relative expression
0.0079
0.0079
0.0317
0.0317
Duox2
Duoxa2
Duox2
Duoxa2
Non-colonized control
Candida colonized
h
Dylight 594 secondary antibody/DAPI

Supplementary Figure 2. Host expression changes induced by *C. albicans* colonization of a germ-free host include *Duox2/Duoxa2*.

Volcano plots depicting differentially expressed genes in germ-free mice that were colonized with *C. albicans* SC5314 cells for 21 days. Comparison of gene expression in ileal (**a**) and colonic (**b**) tissues with/without *C. albicans* colonization. Genes showing expression changes ≥1.5 and p≤0.05 were considered significant. P-values were attained using a Wald test corrected for multiple testing using the Benjamini and Hochberg method. **c-f.** Differentially expressed genes between the datasets are shown by Venn diagram. **c.** Comparison of *C. albicans*-induced gene expression changes between day 7 ileum vs. day 7 colon. **d.** Comparison of *C. albicans*-induced gene expression changes between day 21 ileum vs. day 21 colon samples. **e.** Comparison of *C. albicans*-induced gene expression changes between day 7 ileum vs. day 21 ileum samples. **f.** Comparison of *C. albicans*-induced gene expression changes between day 7 colon vs. day 21 colon samples. **g.** qRT-PCR validation of *Duox2* and *Duoxa2* genes from day 7 colon samples with and without *C. albicans* colonization. Statistical significance is shown as p-values and was determined using a two-tailed Mann-Whitney test. **h**. Immunostaining of colon sections from mice colonized with *C. albicans* with a DyLight 594-coupled secondary antibody (only) shown in red as a negative control. Epithelial nuclei were stained with DAPI. Scale bar, 50 μm. The source data is provided as a Source Data file.

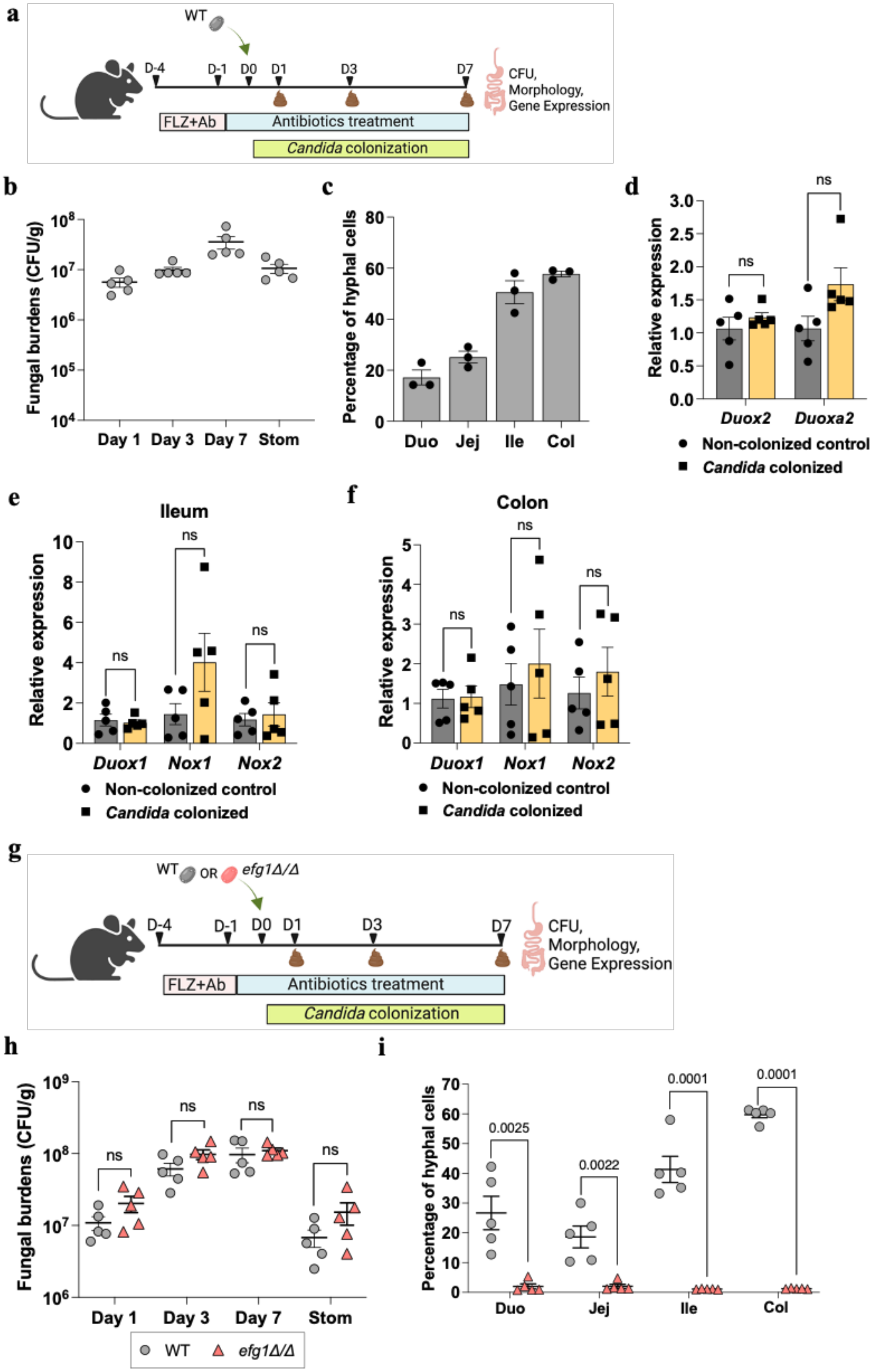
a
WT
D-4
D-1
D0
D1
D3
D7
CFU,
Morphology,
Gene Expression
FLZ+Ab
Antibiotics treatment
Candida colonization
b
Fungal burdens (CFU/g)
Day 1
Day 3
Day 7
Stom
c
Percentage of hyphal cells
Duo
Jej
Ile
Col
d
Relative expression
ns
Duox2
Duoxa2
Non-colonized control
Candida colonized
e
Ileum
Relative expression
ns
Duox1
Nox1
Nox2
Non-colonized control
Candida colonized
f
Colon
Relative expression
ns
Duox1
Nox1
Nox2
Non-colonized control
Candida colonized
g
WT
OR
efg1Δ/Δ
D-4
D-1
D0
D1
D3
D7
CFU,
Morphology,
Gene Expression
FLZ+Ab
Antibiotics treatment
Candida colonization
h
Fungal burdens (CFU/g)
ns
Day 1
Day 3
Day 7
Stom
WT
efg1Δ/Δ
i
Percentage of hyphal cells
0.0025
0.0022
0.0001
0.0001
Duo
Jej
Ile
Col

Supplementary Figure 3. ***C. albicans*** **colonization properties in antibiotic-treated hosts.**
a. Experimental plan. C57BL/6J mice were pre-treated with fluconazole (Flz) and penicillin/streptomycin (Ab) after which they were maintained on Ab (without Flz) for the rest of the experiment. *C. albicans* SC5314 cells were introduced by gavage and fecal samples analyzed at the indicated time points. GI organs were harvested after 7 days of *C. albicans* colonization and analyzed for fungal CFUs, host gene expression and fungal morphology. Created in BioRender. Kakade, P. (2026) and published under a BioRender CC-BY publication license (https://BioRender.com/gilhvtj). **b.** Fungal burdens were analyzed from fecal pellets collected at the indicated time points and from stomach (Stom) harvested on day 7 of colonization. **c.** The proportion of yeast and hyphal cells was determined from GI tissues after staining with an anti-*Candida* antibody. 500-1000 cells were counted from each tissue section. Error bars indicate SEM. **d-f.** qRT-PCR analysis of host genes from the ileum and colon tissues of control and *C. albicans* colonized mice. Data is presented as standard error of mean (SEM) and p-values were determined using a two-tailed Mann-Whitney test. **g.** Experimental plan. C57BL/6J mice were treated with antibiotics and colonized with SC5314 WT cells or *efg1Δ/Δ* cells. Created in BioRender. Kakade, P. (2026) and published under a BioRender CC-BY publication license (https://BioRender.com/mv5818u). **h.** Fungal colonization levels were determined from fecal samples collected on indicated time points and stomach (Stom) harvested on day 7. **i.** Yeast and hyphal cells were enumerated from different parts of the GI tract after staining the sections with anti-*Candida* antibody, DAPI and rhodamine-conjugated UEA1 and WGA1. n= 5 mice per group. Error bars represent SEM. A two-tailed Mann-Whitney test was used to determine statistical significance, ns-not significant. Stom-Stomach, Duo-Duodenum, Jej-Jejunum, Ile-Ileum, Col-Colon. The source data is provided as a Source Data file.

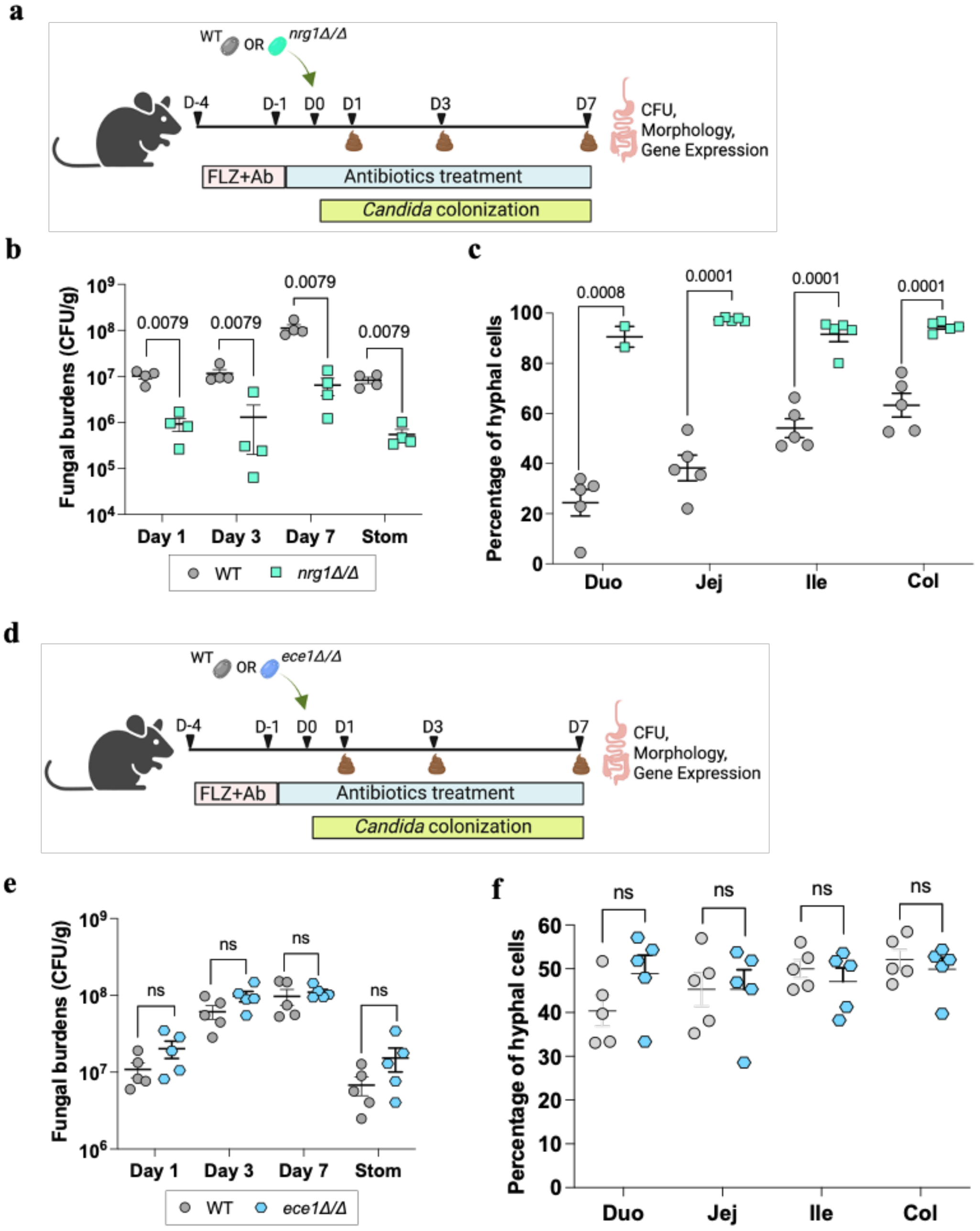

Supplementary Figure 4. Colonization of conventionally housed mice with *C. albicans* WT, hyphal-locked (*nrg1Δ/Δ*) and candidalysin-deficient (*ece1Δ/Δ*) strains.

a. Experimental plan. Antibiotic-treated mice were colonized with SC5314 WT or *nrg1Δ/Δ* (hyphal-locked) strains. n=4 mice per group. Created in BioRender. Kakade, P. (2026) and published under a BioRender CC-BY publication license (https://BioRender.com/lbvfzbt). **b.** Colonization levels of WT and *nrg1Δ/Δ* cells were determined from fecal samples collected at the indicated time points and from stomach (Stom) harvested on 7 dpi. **c.** Proportion of yeast and hyphal cells was determined from small and large intestine upon staining different tissue sections

with anti-*Candida* antibody, DAPI and rhodamine-conjugated UEA1 and WGA1. **d.** Experimental details for the colonization of antibiotic-treated mice with WT or *ece1Δ/Δ* (candidalysin-deficient) cells. n=5 mice per group. Created in BioRender. Kakade, P. (2026) and published under a BioRender CC-BY publication license (https://BioRender.com/2e1bawp). **e.** Fungal burdens were evaluated from fecal samples and stomach. Error bars represent SEM. A two-tailed unpaired t-test was used to determine statistical significance. ns-not significant. **f.** Proportion of hyphal cells in GI organs colonized with WT or *ece1Δ/Δ* cells. Error bars represent SEM. p-values were determined using a two-tailed Mann-Whitney test, ns-not significant. Stom-Stomach, Duo-Duodenum, Jej-Jejunum, Ile-Ileum, Col-Colon. The source data is provided as a Source Data file.

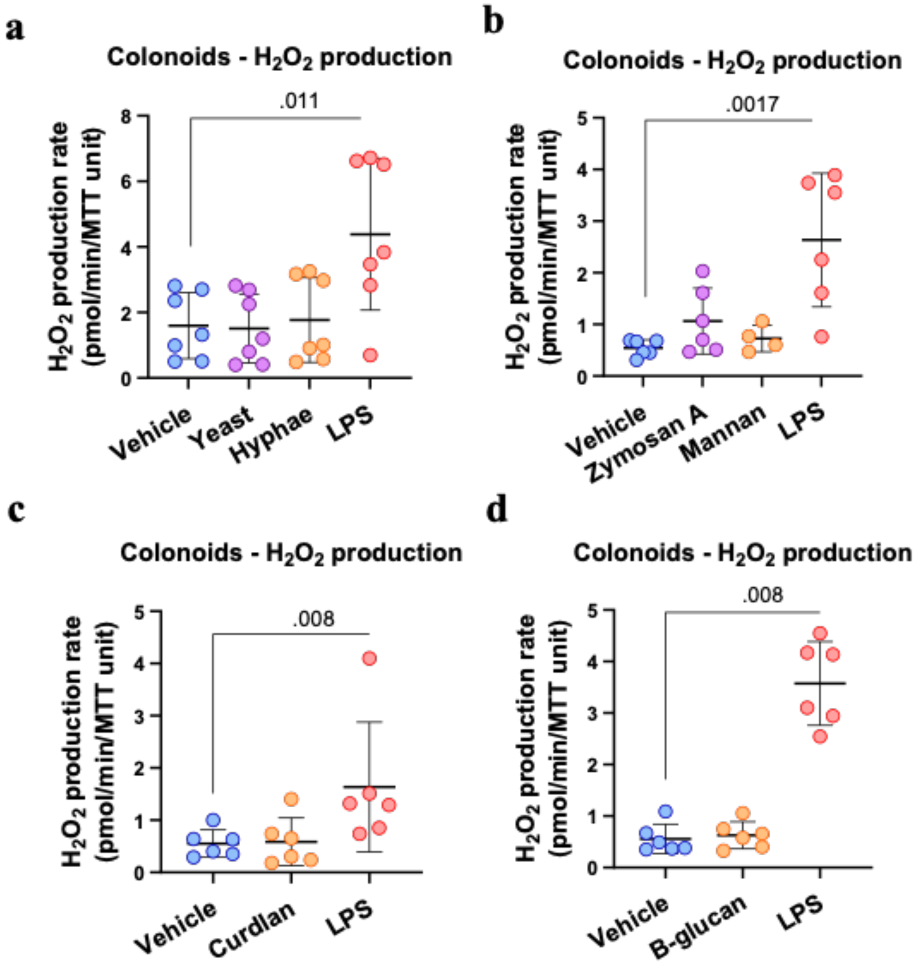

Supplementary Figure 5. Fungal cell wall components do not induce H_2O_2 production in colonoids.

Colonoids were stimulated for 24 h with PMB-pretreated **(a)** 10^7 cells/mL of heat killed *C. albicans* yeast and hyphae (n=7 cultures); **(b)** 100 µg/mL of curdlan prepared in DMSO (n=6 cultures); **(c)** 250 µg/mL of mannan and zymosan A from *S. cerevisiae* prepared in 1:1 PBS/DMSO (n=4-6 cultures); **(d)** 100 µg/mL of β-glucan from *S. cerevisiae* prepared in 1:1 PBS/DMSO (n=6 cultures). H_2O_2 production rates were normalized to MTT viability values. Data were analyzed by means of a Friedman test for matched samples in experiments in (**a**), (**b**), and (**d**); Kruskal-Wallis test (**c**). The source data is provided as a Source Data file.

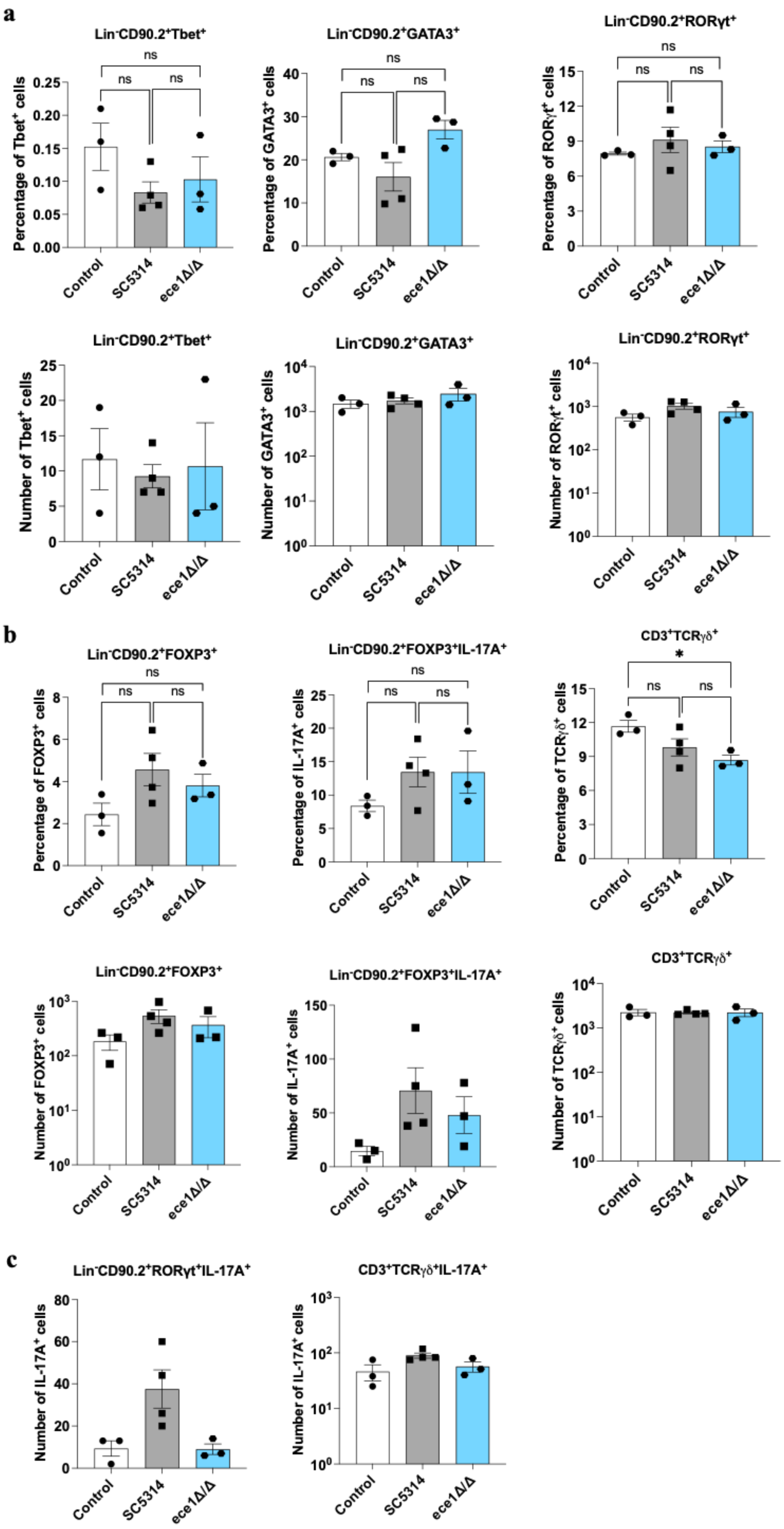
a
Lin⁻CD90.2⁺Tbet⁺
Percentage of Tbet⁺ cells
Lin⁻CD90.2⁺GATA3⁺
Percentage of GATA3⁺ cells
Lin⁻CD90.2⁺RORγt⁺
Percentage of RORγt⁺ cells
Number of Tbet⁺ cells
Number of GATA3⁺ cells
Number of RORγt⁺ cells
b
Lin⁻CD90.2⁺FOXP3⁺
Percentage of FOXP3⁺ cells
Lin⁻CD90.2⁺FOXP3⁺IL-17A⁺
Percentage of IL-17A⁺ cells
CD3⁺TCRγδ⁺
Percentage of TCRγδ⁺ cells
Number of FOXP3⁺ cells
Number of IL-17A⁺ cells
Number of TCRγδ⁺ cells
c
Lin⁻CD90.2⁺RORγt⁺IL-17A⁺
Number of IL-17A⁺ cells
CD3⁺TCRγδ⁺IL-17A⁺
Control
SC5314
ece1Δ/Δ
ns
*

Supplementary Figure 6. ***C. albicans*** **mediated candidalysin-dependent early immune responses and the gating strategies used.**

a-c. The proportion of innate lymphoid cells and γδ T cells was compared between non-colonized controls, SC5314-colonized and *ece1*Δ/Δ-colonized mice. The percentage and numbers of ILC1 gated as $CD45^+$, Lin^-, $CD90.2^+$, $Tbet^+$, ILC2 gated as $CD45^+$, Lin^-, $CD90.2^+$, $GATA3^+$, ILC3 gated as $CD45^+$, Lin^-, $CD90.2^+$, $ROR\gamma t^+$, γδ T cells gated as $CD45^+$, $CD3^+$, $TCR\gamma\delta^+$. n=3-4 mice per group. Error bars indicate SEM. A two-tailed unpaired t-test was used to determine statistical significance, ns-not significant. The source data is provided as a Source Data file.

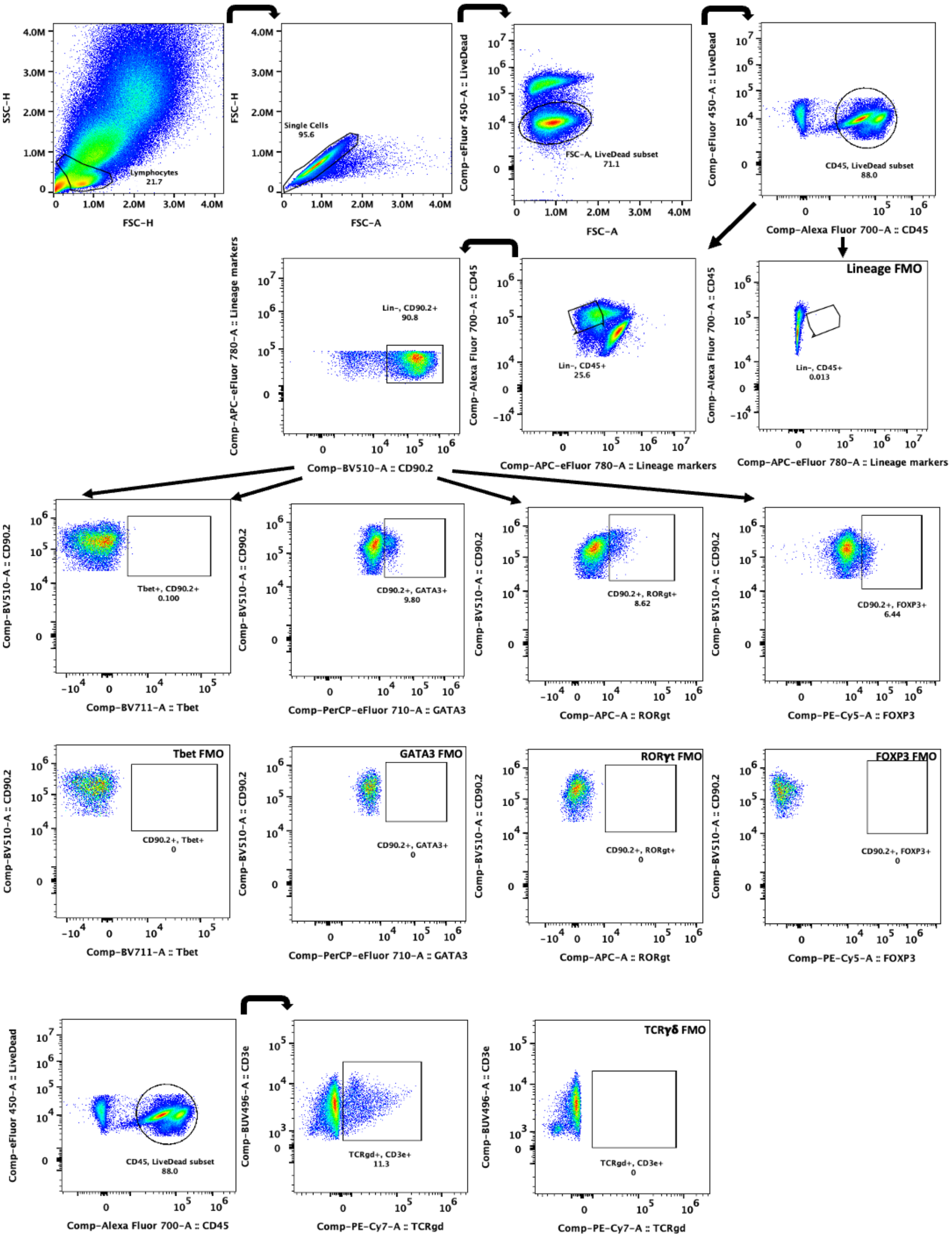

Supplementary Figure 7. Strategies used to gate different immune cell populations with FMO controls.

Strategies to gate different innate cell populations from the colon lamina propria of control, SC5314-colonized and *ece1*Δ/Δ-colonized mice after 7 days of colonization.

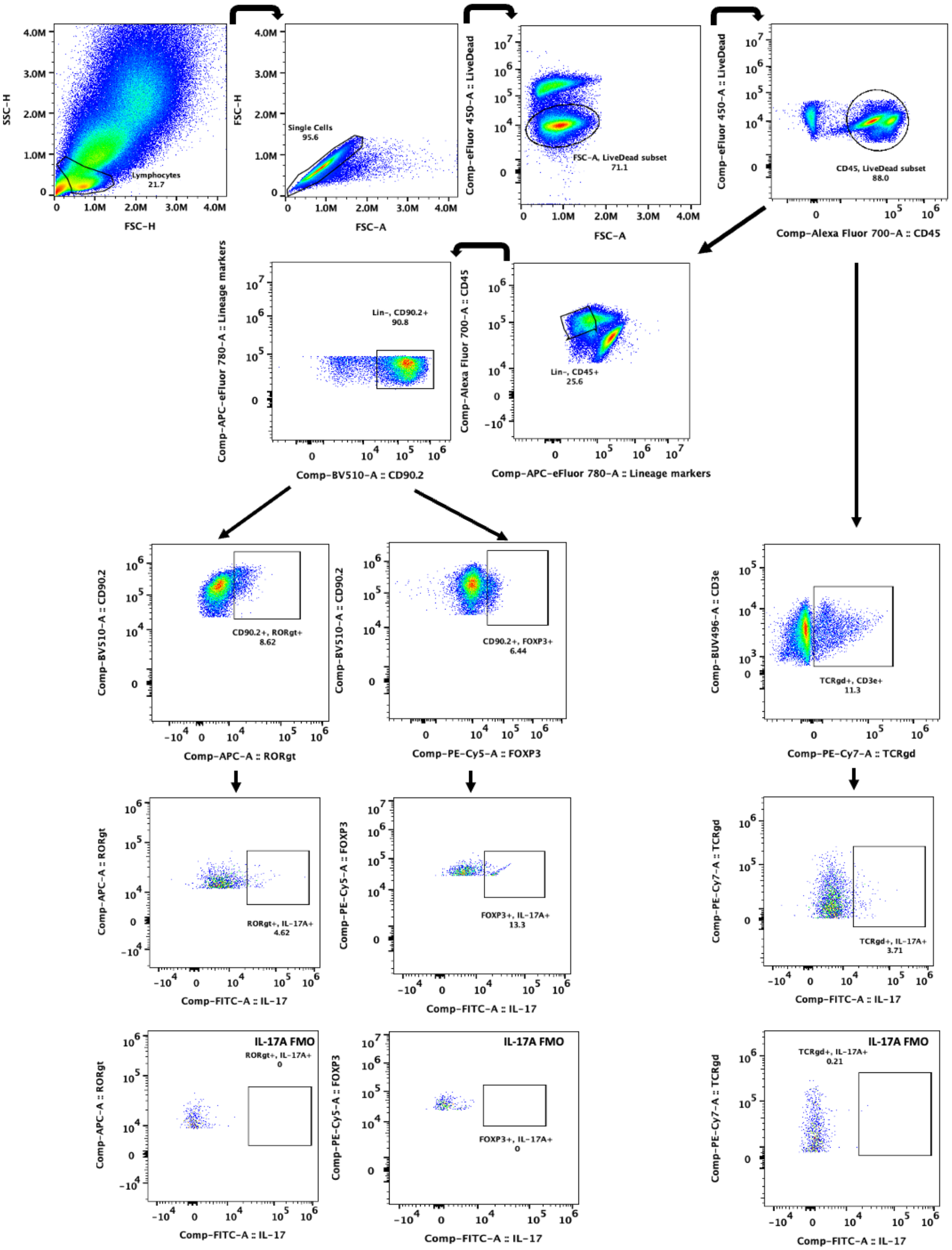

Supplementary Figure 8. Strategies used to gate IL-17A producing immune cell populations with FMO controls.

Strategies used to gate different IL-17A producing innate immune cells from the colon lamina propria of control SC5314-colonized and *ece1*Δ/Δ-colonized mice after 7 days of colonization.

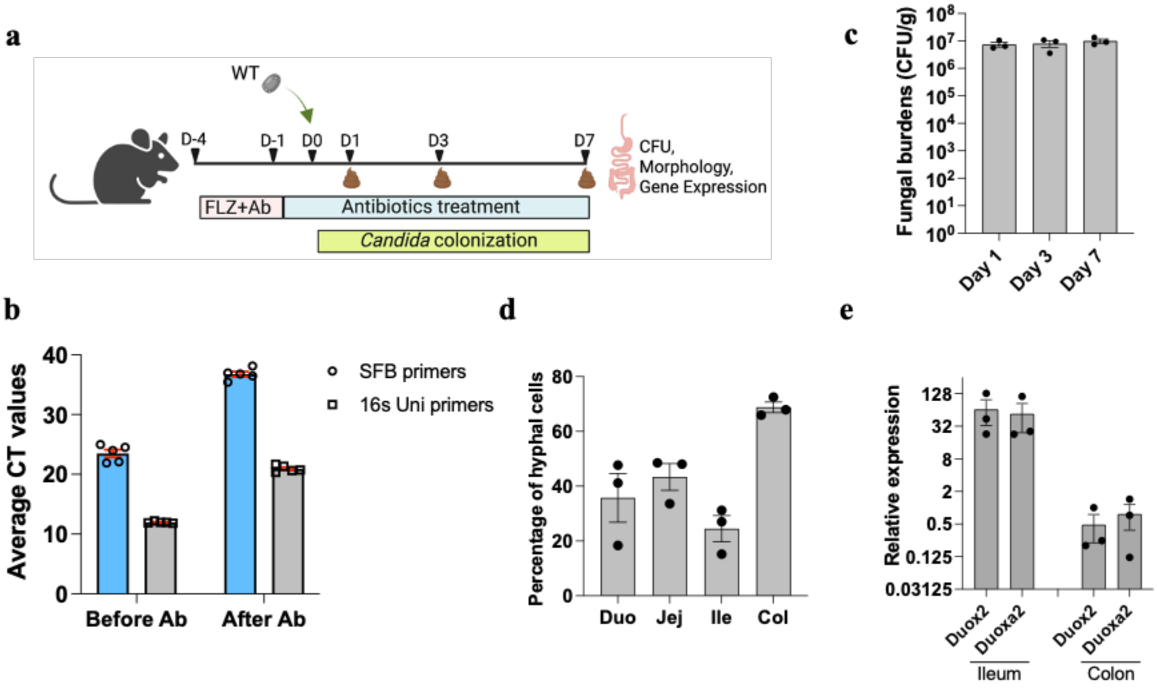

Supplementary Figure 9. Analysis of *C. albicans* colonization and gene expression in hosts that previously housed segmented filamentous bacteria (SFB).

a. Experimental plan. C57B6/Tac mice (Taconic Biosciences) were pre-treated with fluconazole (Flz) and antibiotics penicillin, streptomycin, and vancomycin (Ab) prior to inoculation with *C. albicans*. Antibiotic treatment was then continued for the remainder of the experiment. Created in BioRender. Kakade, P. (2026) and published under a BioRender CC-BY publication license (https://BioRender.com/gilhvtj). **b.** Antibiotic clearance of gut bacteria was evaluated by qPCR on genomic DNA isolated from fecal samples using 16S universal primers or primers specific for SFB. Higher CT values indicate lower bacterial loads and vice versa. **c.** Fungal burdens were evaluated from fecal samples collected 1, 3 or 7 dpi. n=2 for control group and n=3 for *C. albicans* colonized group. **d.** Yeast and hyphal percentage was determined after staining sections of the indicated GI organs with an anti-*Candida* antibody. 500-1000 fungal cells were evaluated for each mouse and error bars indicate SEM. **e.** Transcript levels of host genes were analyzed from ileum and colon tissues of both groups of mice and are presented as relative expression values. Error bars indicate SEM. The source data is provided as a Source Data file.

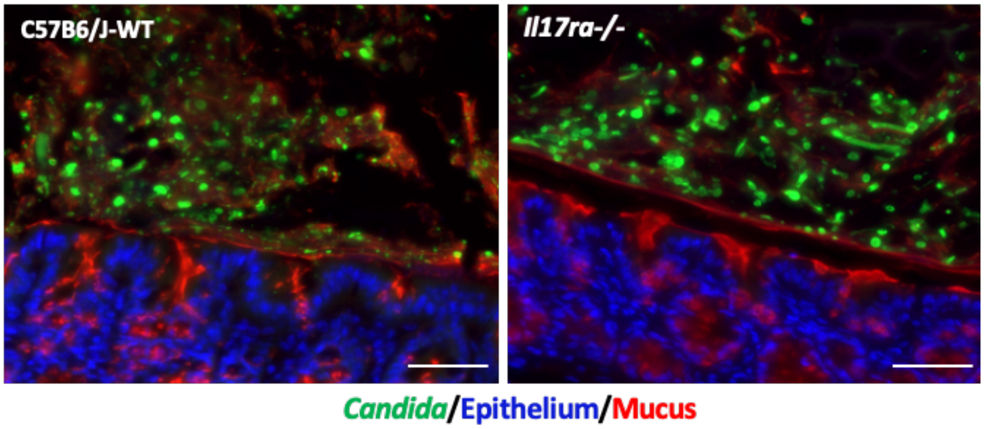

Supplementary Figure 10. Yeast hyphal morphologies from WT control and *Il17ra-/-* mice.

Colon tissue sections from WT control and *Il17ra-/-* mice were stained with anti-*Candida* antibody, epithelial nuclei with DAPI and mucus with rhodamine conjugated UEA1 and WGA1. Scale bar- 50 µm.

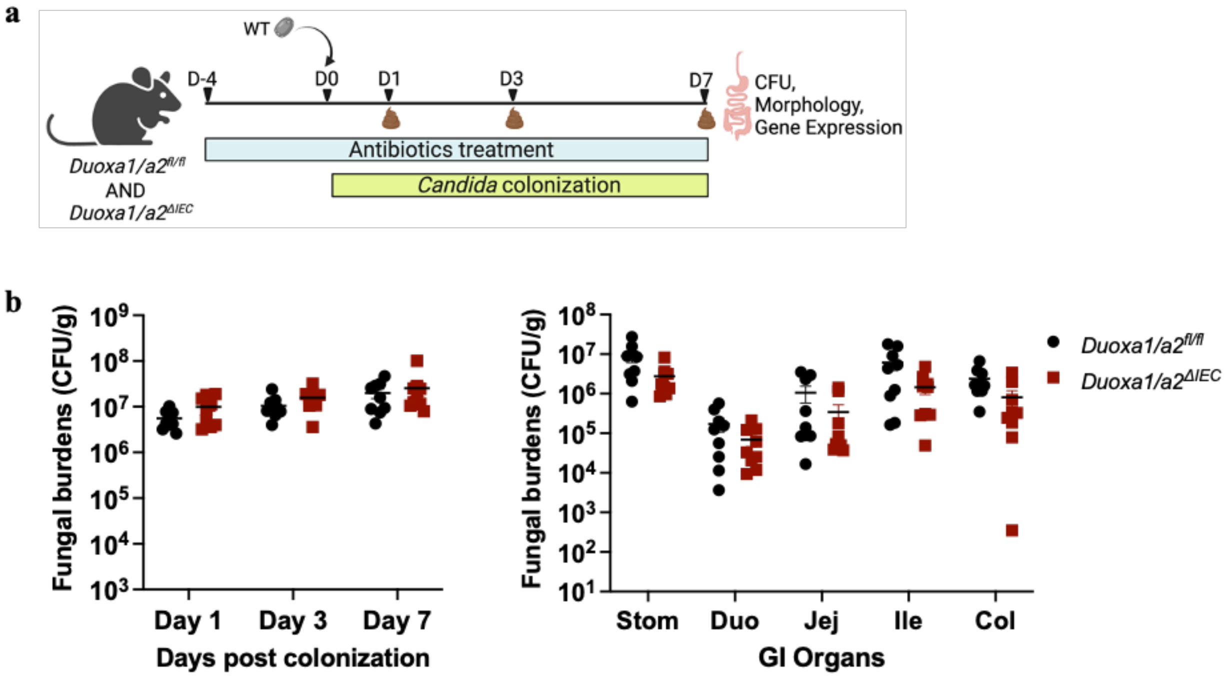

Supplementary Figure 11. ***C. albicans*** **colonization in control** ***Duoxa1/a2***$^{fl/fl}$ **mice and in DUOX2-deficient** ***Duoxa1/a2***$^{\Delta IEC}$ **mice.**

a. Experimental plan. Created in BioRender. Kakade, P. (2026) and published under a BioRender CC-BY publication license (https://BioRender.com/xfgfk1i). **b.** Colonization levels of *C. albicans* were determined from fecal pellets collected 1, 3 and 7 dpi. GI organs were harvested from *Duoxa1/a2*$^{fl/fl}$ and *Duoxa1/a2*$^{\Delta IEC}$ mice and analyzed for fungal burdens at 7 dpi. n=9 mice per group. Error bars indicate SEM. The source data is provided as a Source Data file.

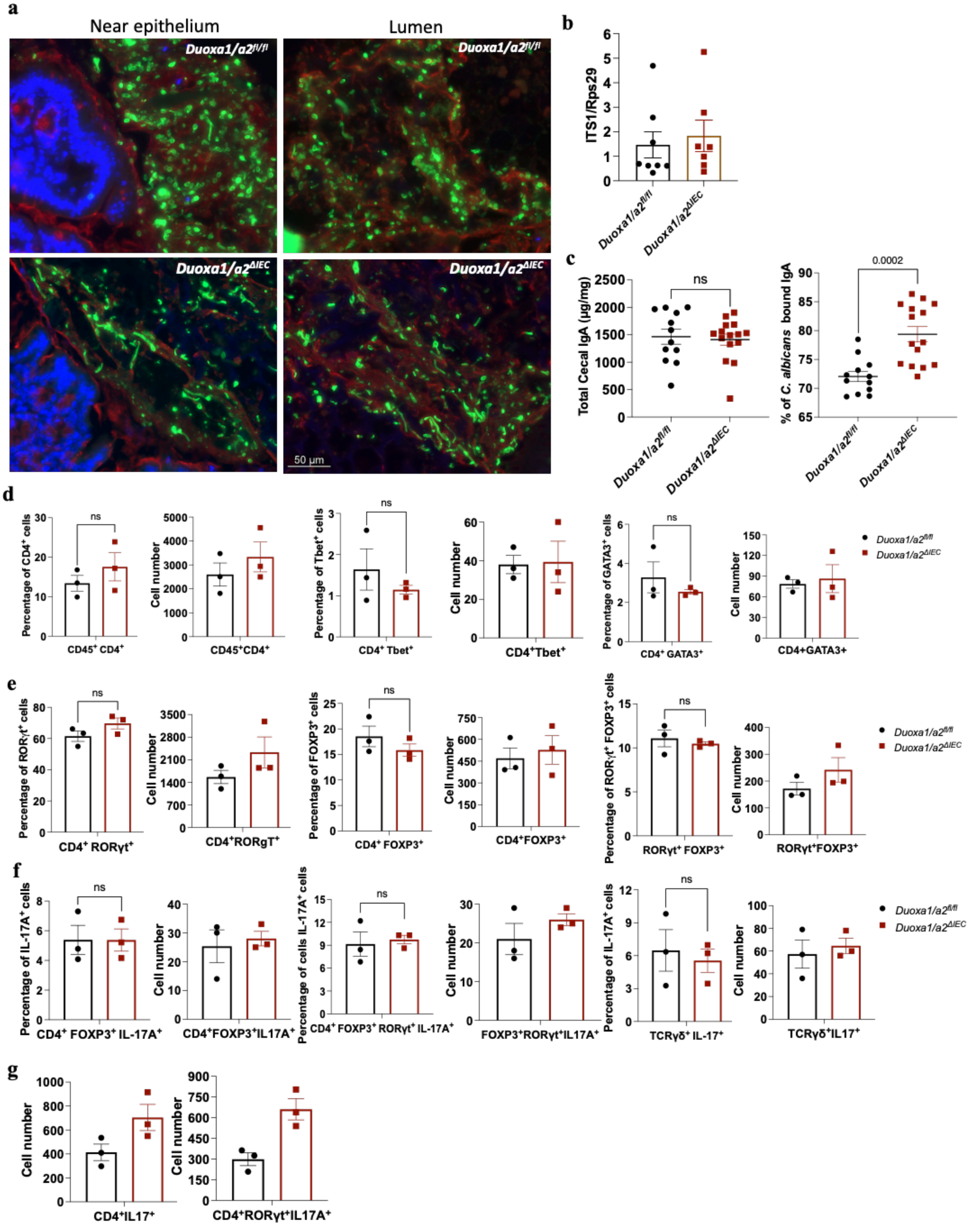
a
Near epithelium
Lumen
Duoxa1/a2fl/fl
Duoxa1/a2ΔIEC
50 µm
b
ITS1/Rps29
c
Total Cecal IgA (µg/mg)
ns
% of C. albicans bound IgA
0.0002
d
Percentage of CD4+ cells
Cell number
Percentage of Tbet+ cells
Percentage of GATA3+ cells
CD45+ CD4+
CD4+ Tbet+
CD4+ GATA3+
CD4+GATA3+
e
Percentage of RORγt+ cells
Percentage of FOXP3+ cells
Percentage of RORγt+ FOXP3+ cells
CD4+ RORγt+
CD4+RORgT+
CD4+ FOXP3+
RORγt+ FOXP3+
f
Percentage of IL-17A+ cells
Percentage of cells IL-17A+ cells
CD4+ FOXP3+ IL-17A+
CD4+ FOXP3+ RORγt+ IL-17A+
FOXP3+RORγt+IL17A+
TCRγδ+ IL-17+
TCRγδ+IL17+
g
CD4+IL17+
CD4+RORγt+IL17A+

Supplementary Figure 12. Absence of functional DUOX2 promotes *C. albicans* filamentation in the gut. a. Colonic tissue sections were stained with an anti-*Candida* antibody to assess morphological forms. Epithelial nuclei were stained with DAPI and mucus was stained with rhodamine-conjugated WGA-1 and UEA-1. Scale bar, 50 μm. **b.** The transepithelial translocation of *C. albicans* cells to mesenteric lymph nodes (MLNs) was assessed by carrying out qPCR on genomic DNA isolated from MLNs using *C. albicans*-specific ITS1 primers. Normalization was carried out using host-specific *Rps29* primers. **c**. The total IgA and *C. albicans* hyphae bound IgA were determined from cecal supernatants of $Duoxa1/a2^{fl/fl}$ and $Duoxa1/a2^{\Delta IEC}$ mice by ELISA and flow cytometry analysis, respectively. **d-g.** Percentage and numbers of lymphocytes and different T-cell subsets. $CD4^+$ lymphocytes gated as ($CD45^+$ $CD4^+$), Th1 (gated as $CD4^+$ $Tbet^+$), Th2 (gated as $CD4^+$ $GATA3^+$), Th17 (gated as $CD4^+$ $ROR\gamma t^+$). Percentage and numbers of Treg cells (gated as $CD4^+$ $FOXP3^+$), FOXP3 and RORγt expressing double positive cells, IL-17 producing TCRγδ T-cells (gated as $CD45^+$ $CD90.2^+$ $TCR\gamma\delta^+$ $IL\text{-}17^+$). Error bars indicate SEM. Statistical significance was determined using a two-tailed unpaired t-test. The source data is provided as a Source Data file.

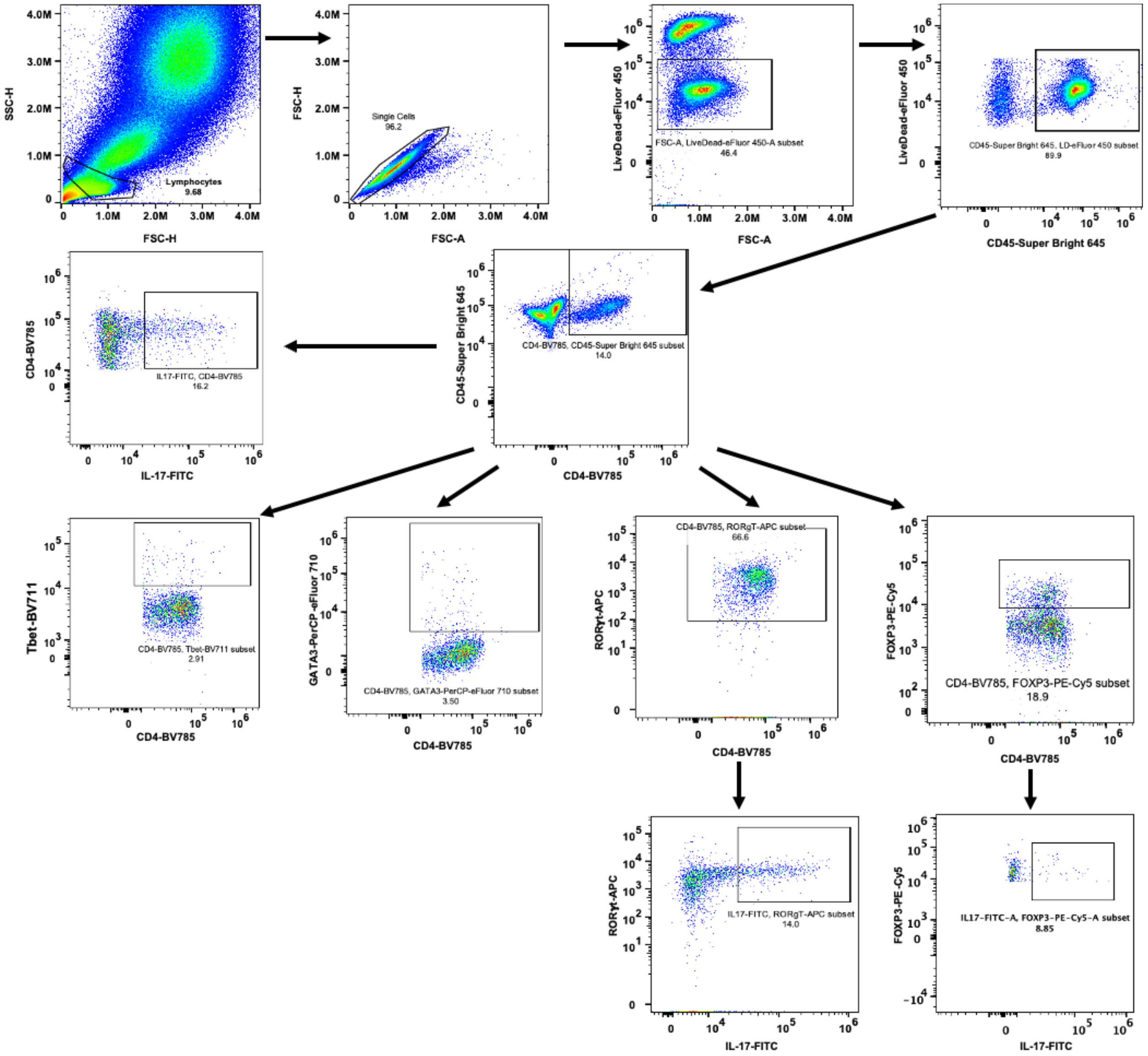

Supplementary Figure 13. Strategies used to gate different immune cell populations from the lamina propria.

Steps to gate lymphocytes (Th1, Th2, Th17 and Treg) isolated from lamina propria of *C. albicans* colonized *Duoxa1/a2*$^{fl/fl}$ and *Duoxa1/a2*$^{\Delta IEC}$ mice and to gate IL-17A producing Th17 and Treg cells.

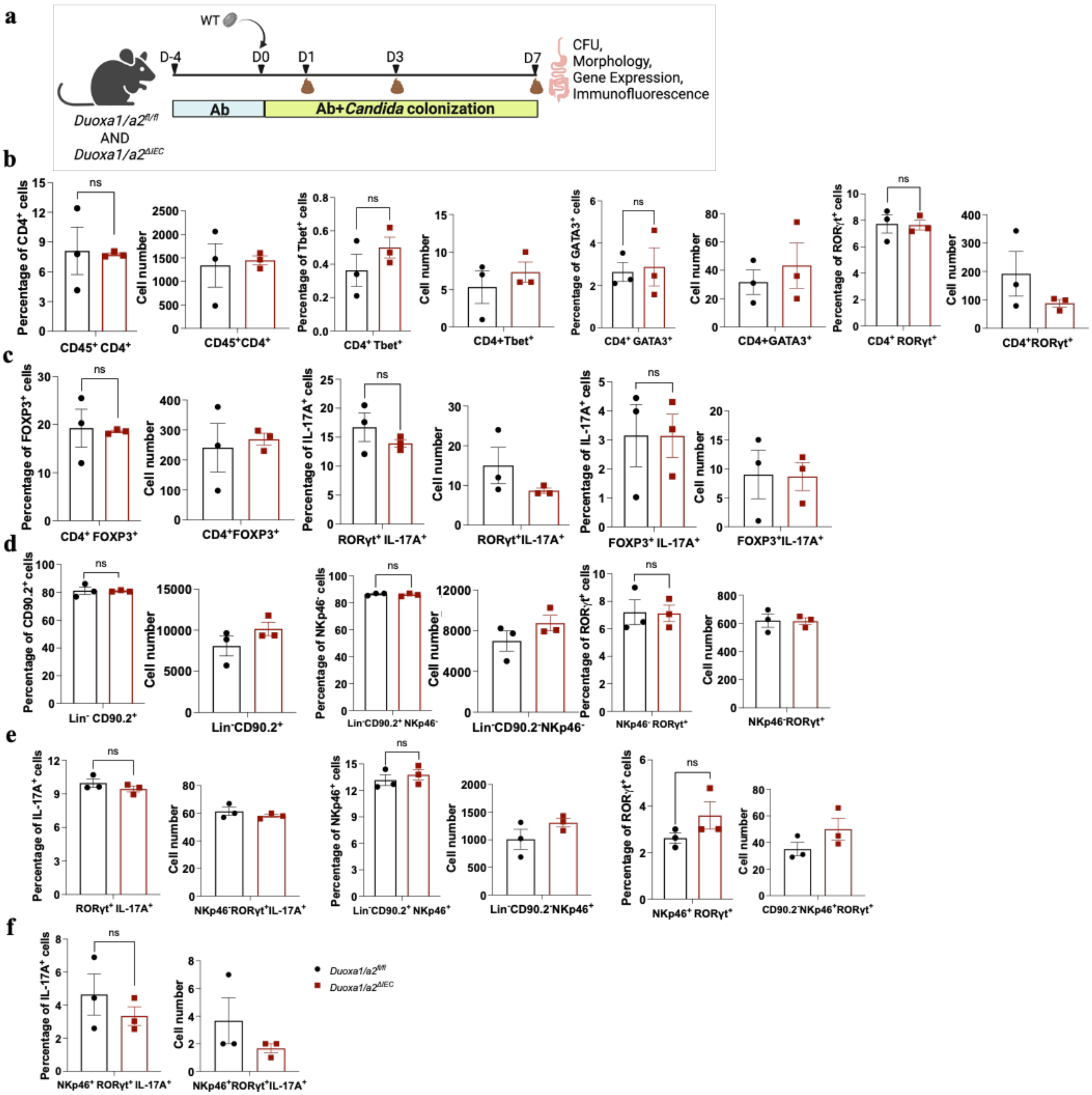

Supplementary Figure 14. Immune responses in WT vs. DUOX2-deficient mice upon *C. albicans* colonization for 7 days.

a. Experimental plan. Wild type and DUOX2-deficient mice were colonized with *C. albicans* for 7 days and immune cells obtained from the colon lamina propria. Created in BioRender. Kakade, P. (2026) and published under a BioRender CC-BY publication license (https://BioRender.com/odrox9n). **b-e.** Percentages and numbers of different cell types in the lamina propria. $CD4^+$ (gated as $CD45^+$ $CD4^+$), Th1 (gated as $CD4^+$ $Tbet^+$), Th2 (gated as $CD4^+$ $GATA3^+$), and Th17 (gated as $CD4^+$ $ROR\gamma t^+$) cells. Percentage and numbers of Treg (gated as $CD4^+$ $FOXP3^+$), IL-17 producing Th17 (gated as $CD4^+$ $ROR\gamma t^+$ $IL\text{-}17^+$) and Treg (gated as $CD4^+$

FOXP3^{+} IL-17^{+}) cells. Percentage and numbers of different cell types obtained while gating for ILC3 cells. Lineage negative -CD90.2 positive cells, non-pathogenic CD90.2 expressing cells (gated as CD90.2^{+} NKp46^{-}), non-pathogenic RORγt expressing ILC3 cells (gated as CD90.2^{+} NKp46^{-} RORγt^{+}), non-pathogenic IL-17 producing ILC3 cells (gated as NKp46^{-} RORγT^{+} IL-17^{+}). Proportion of pathogenic, CD90.2 expressing cells (gated as CD90.2^{+} NKp46^{+}), pathogenic RORγt expressing ILC3 cells (gated as CD90.2^{+} NKp46^{+} RORγt^{+}), and pathogenic IL-17 producing ILC3 cells gated as (NKp46^{+} RORγt^{+} IL-17^{+}). Error bars indicate SEM. Statistical significance was determined using a two-tailed unpaired t-test. The source data is provided as a Source Data file.

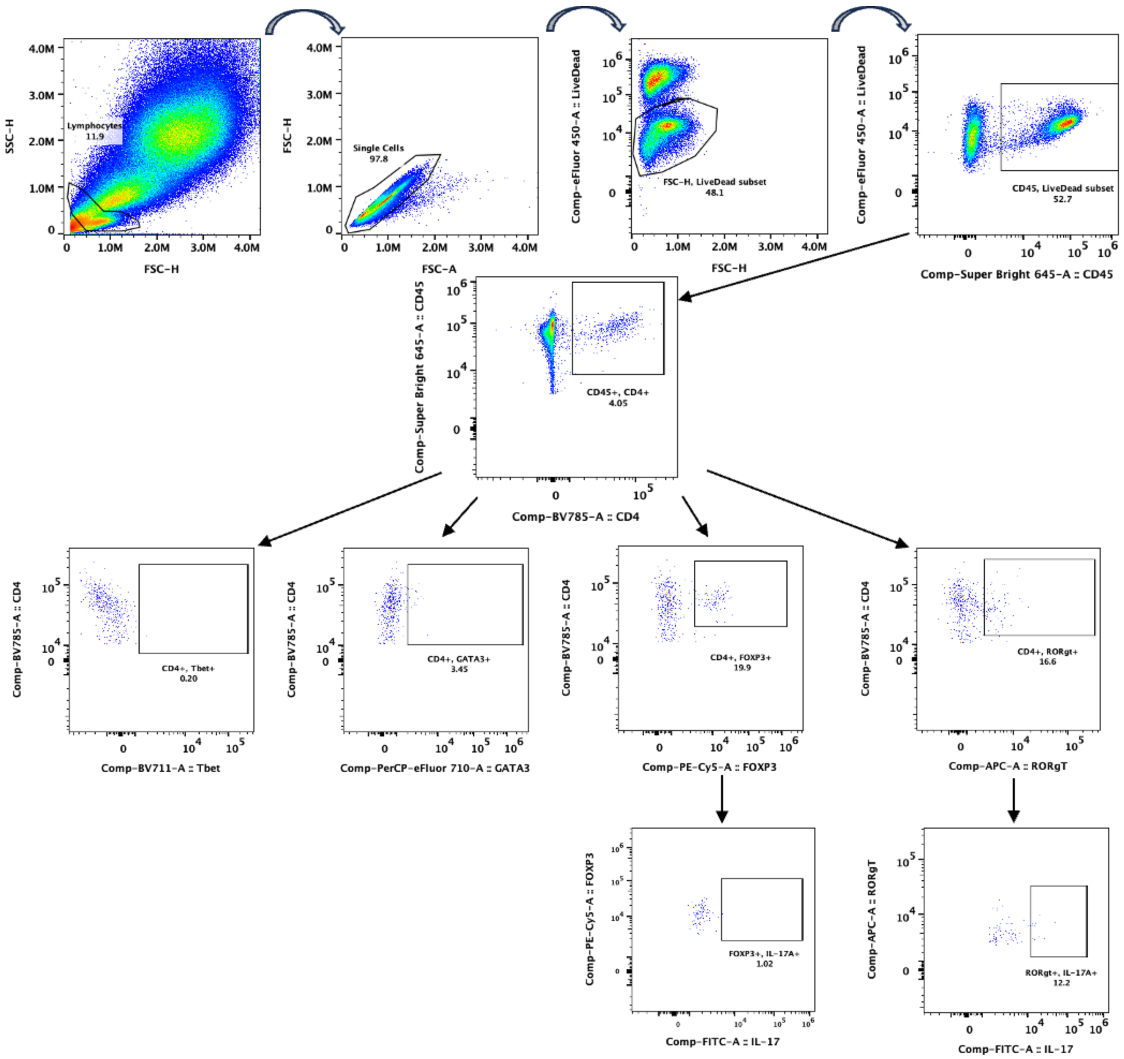

Supplementary Figure 15. Gating strategy for CD4+ T-cell subsets after 7 days colonization with *C. albicans* in *Duox2*-floxed and *Duox2*-deficient mice.

Steps followed to gate CD4^{+} cells (Th1, Th2, Th17 and Treg) isolated from the lamina propria of *C. albicans*-colonized *Duoxa1/a2*$^{fl/fl}$ and *Duoxa1/a2*$^{\Delta IEC}$ mice for 7 days.

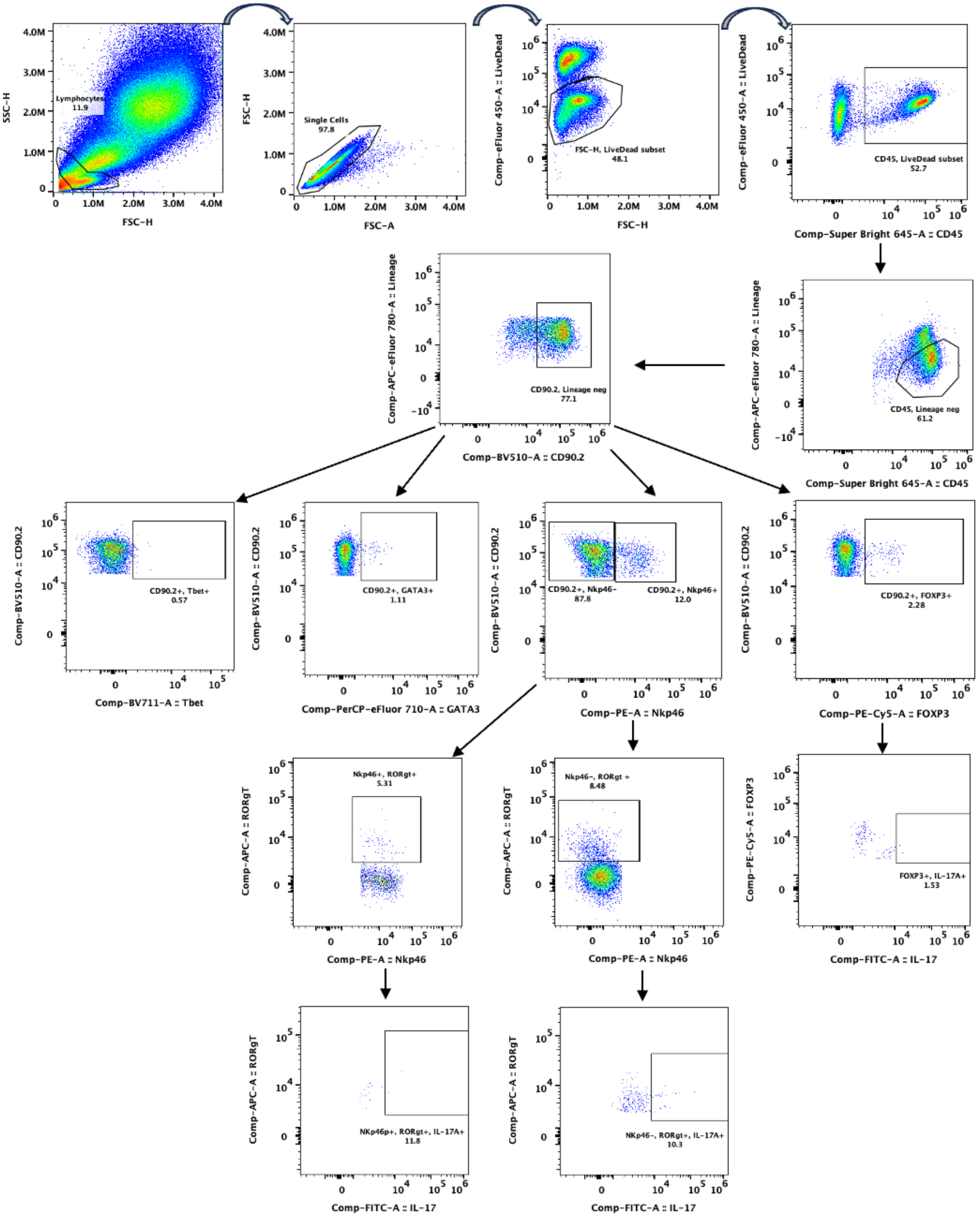

Supplementary Figure 16. Gating strategy for innate lymphoid cells upon 7 days colonization of *C. albicans* in *Duox2*-floxed and *Duox2*-deficient mice.

Steps followed to gate innate lymphoid cells (ILC1, ILC2 and ILC3) and γδ T-cells isolated from the lamina propria of *C. albicans*-colonized *Duoxa1/a2*$^{fl/fl}$ and *Duoxa1/a2*$^{\Delta IEC}$ mice after 7 days.

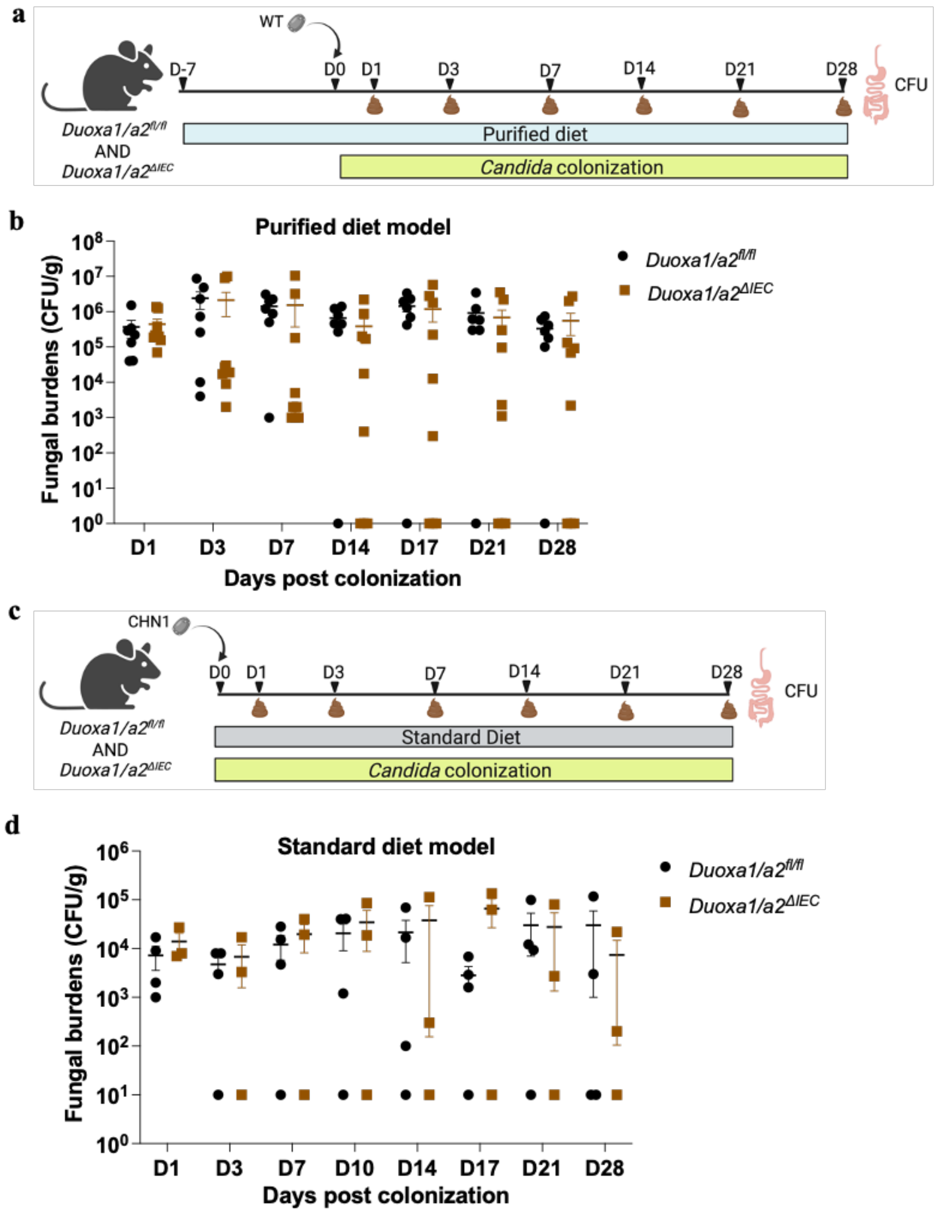

Supplementary Figure 17. Role of DUOX2 in regulating *C. albicans* colonization in purified and standard diet models without antibiotic intervention. a. Experimental plan for colonization of purified diet fed *Duoxa1/a2$^{fl/fl}$* and *Duoxa1/a2$^{\Delta IEC}$* mice over a period of 28 days. Created in BioRender. Kakade, P. (2026) and published under a BioRender CC-BY publication license (https://BioRender.com/kr9wbau). **b.** Fungal burdens were determined from fecal samples collected over a period of 28 days from purified diet fed *Duoxa1/a2$^{fl/fl}$* and *Duoxa1/a2$^{\Delta IEC}$* mice. n=6-8 mice per group. No significant differences were found. **c.** Experimental plan for colonization of standard diet fed *Duoxa1/a2$^{fl/fl}$* and *Duoxa1/a2$^{\Delta IEC}$* mice over a period of 28 days. Created in BioRender. Kakade, P. (2026) and published under a BioRender CC-BY publication license (https://BioRender.com/nwlbkt4). **d**. *C. albicans* CFUs were determined from fecal samples collected over a period of 28 days from SD fed *Duoxa1/a2$^{fl/fl}$* and *Duoxa1/a2$^{\Delta IEC}$* mice.

n=3-4 mice per group. Error bars indicate SEM. Statistical significance was determined using a two-tailed unpaired t-test. The source data is provided as a Source Data file.

Supplementary Table 1: *C. albicans* Strains used in this study

Strain	Genotype	Source
SC5314	Wild type	Fonzi and Irwin
*efg1*Δ/Δ	*efg1*::*FRT*/*efg1*::*FRT* in SC5314	[1]
*nrg1*Δ/Δ	*nrg1*::*FRT*/*nrg1*::*FRT* in SC5314	David Kadosh laboratory
SN95A+H+	SN95 strain prototroph for Arg and His	This study
*ece1*Δ/Δ	*ece1*::*FRT*/*ece1*::*FRT* in SN95A+H+	[2]

Supplementary Table 2: Mouse strains used in this study

**Strain**	**Source**	**Details**
C57BL/6J	Jackson Laboratories	Strain#000664
C57BL/6NTac	Taconic Biosciences	B6-MPFF
Duoxa1/a2$^{fl/fl}$	Dr. Jonathan Kaunitz's laboratory	*Duoxa1/2* -floxed
*Duoxa1/a2*Δ^{IEC}	Dr. Jonathan Kaunitz's laboratory	Epithelial specific knockouts of *Duoxa1/a2*
Villin-cre	Jackson Laboratories	Tg[Vil1-cre]997Gum
IL17ra-/-	Dr. Sarah Gaffen's laboratory	Mice lacking IL17 receptor A.

Supplementary Table 3: Primers used in this study

**Primer**	**Sequence**
Duox2 qRT_F1	CTGTGAGCATGAAGCCATTC
Duox2 qRT_R1	CGGGTAGAGGTAACATCTGT
Duoxa2 qRT_F1	CTTCGCTCCTTTCTGGATCT
Duoxa2 qRT_R1	CCCAAAGAGGGGTAGAAATC
Rps29 qRT_F	GGAGTCACCCACGGAAGTT
Rps29 qRT_R	GAAGCCTATGTCCTTCGCGT
Actb qRT_F	TGACAGGATGCAGAAGGAGA
Actb qRT_R	CGCTCAGGAGGAGCAATG
Gusb qRT_F	CCGATTATCCAGAGCGAGTATG
Gusb qRT_R	CTCAGCGGTGACTGGTTCG

Duox2 qRT_F2	TCCAGAAGGCGCTGAACAG
Duox2 qRT_R2	GCGACCAAAGTGGGTGATG
Duoxa2 qRT_F2	GCCTGGCTTTGCTCACCA
Duoxa2 qRT_R2	GAGGAGGAGGCTCAGGAT
16775	GCCTTCTCCTCTAGGCTCGT
16776	TATAGGGCAGAGCTGGAGGA
oIMR9074	AGGCAAATTTTGGTGTACGG
SFB_F	GACGCTGAGGCATGAGAGCA
SFB_R	GACGGCACGGATTGTTATTC
7212	ACTCCTACGGGAGGCAGCAGT
7213	ATTACCGCGGCTGCTGGC
ITS1-34F	CTGCCCTTTGTACACACCGC
ITS2-R	GCTGCGTTCTTCATCGATGC
Duox1 qRT_F	GTCAGCTCATCAACAGGCAG
Duox1 qRT_R	CCTACACATGATGCCTGAGC
Nox1 qRT_F	GCTGTAAATCCTGGGAACCT
Nox1 qRT_R	CTTAGTGGGTGATATGGGAG
Nox2 qRT_F	GAAAGCACCACACTCACAAG
Nox2 qRT_R	TCCCTTCGTCTAGTCCCTTC

Supplementary Table 4: List of reagents used in this study

**Reagents and Kits**			
	**Product**	**Company**	**Catalogue no.**
	JM109 comp cells	Promega	L2001
	Nourseothricin	Werner brothers	5.010.000
	Penicillin	Fisher	BP914-100
	Ampicillin	Fisher Scientific	BP1760-25
	Streptomycin	MP Biomedicals	219454180
	Kanamycin	Fisher	BP906-5
	Chloramphenicol	Fisher Scientific	BP904-100
	Doxycycline	Sigma	D9891-5G
	Vancomycin	Sigma-Aldrich	V2002-1G
	Fluconazole	Sigma-Aldrich	PHR1160-1G
	Lyticase	Sigma-Aldrich	L2524-50KU
	Methacarn	American Master Tech Scientific	NC0547175

	DAPI	Molecular Probes, Invitrogen	D1306
	WGA1-Fluorescein	Vector laboratories	FLK-2100
	UEA1-Fluorescein	Vector laboratories	FLK-2100
	WGA1-Rhodamine	Vector laboratories	RL-1022-5
	UEA1-Rhodamine	Vector laboratories	RL-1062-2
	Percoll	Sigma	P1644
	RPMI 1640 Medium, GlutaMAX™ Supplement	ThermoFisher	61870127
	HBSS, no calcium, no magnesium	ThermoFisher	14170161
	NaHCO3	Millipore Sigma	1370131000
	FBS	Gibco/Thermo Fischer	
	Pen-Strep	Gibco	15070063
	Na-Pyruvate	Thermo Fisher	11360070
	dPBS	Thermo Fisher Scientific	10010023
	DTT	Fisher Scientific	BP172-25
	DNase I	Millipore Sigma	11284932001
	Collagenase	Sigma-Aldrich	C2139-100MG
	Protein Transport Inhibitor	ThermoFischer	00-4980-03
	Cell Stimulation Cocktail	ThermoFischer	00-4970-03
	BSA	Fisher Scientific	B9000S
	rmIL17A	R&D Systems	7956-ML-025
	Mannan	Sigma-Aldrich	M7504-1G
	Curdlan	Invivogen	tlrl-curd
	Zymosan A	Sigma-Aldrich	Z4250-1G
	Beta-glucan	Sigma-Aldrich	346210-25MG
	EDTA	Thermo Fisher	15575020
	HBSS	Thermo Fisher	14175095
	Cultrex reduced GF basement membrane type R1	Cultrex	3433-005-R1
	Chir99021	Cayman chemical	13122
	Thiazovivin	Cayman chemical	14245
	Primocin	Invivogen	ant-pm-2
	Polymyxin B	Millipore Sigma	5291-500MG
	LPS	Invivogen	tlrl-3 pelps
	Amplex red	Biotium	10061

	DPBS	Thermo Fisher	14040117
	HRP	Millipore Sigma	P8125-5KU
	MTT	Cayman chemical	21795
	DMEM/F12	Thermo Fisher	11320033
	DMSO	Millipore Sigma	317275
	PMSF	Millipore Sigma	P7626-1G
**Antibodies**			
	**Product**	**Company**	**Catalogue no.**
	Anti-Candida antibody	Thermo Fisher Scientific	PA173154
	Anti-DUOX2 antibody	Novus Biologicals	NB110-61576
	Donkey anti-Rabbit IgG (H+L) Cross-Adsorbed Secondary Antibody, DyLight™ 594	ThermoFischer	SA5-10040
	Biotin anti-mouse Lineage Panel	BioLegend	133307
	Brilliant Violet 785™ anti-mouse CD4 Antibody	BioLegend	100551
	CD45 Monoclonal Antibody (2D1), Super Bright™ 645, eBioscience™	eBioscience	64-9459-42
	Brilliant Violet 510™ anti-mouse CD90.2 (Thy-1.2) Antibody	BioLegend	140319
	CD335 (NKp46) Monoclonal Antibody (29A1.4), PE, eBioscience	ThermoFisher	12-3351-80
	Brilliant Violet 711™ anti-T-bet Antibody	BioLegend	644819
	Gata-3 Monoclonal Antibody (TWAJ), PerCP-eFluor™ 710, eBioscience™	eBioscience	46-9966-41
	ROR gamma (t) Monoclonal Antibody (B2D), APC, eBioscience™	eBioscience	17-6981-80
	FOXP3 Monoclonal Antibody (FJK-16s), PE-Cyanine5, eBioscience™	eBioscience	15-5773-80
	Alexa Fluor® 700 anti-mouse CD45 Antibody	BioLegend	147716
	IL-17A Monoclonal Antibody (eBio17B7), Alexa Fluor 488, eBioscience™	Thermo Scientific	53-7177-81
	PE/Cyanine7 anti-mouse TCR γ/δ Antibody	BioLegend	118123
	CD3e Monoclonal Antibody (145-2C11), Brilliant Ultra Violet™ 496, eBioscience™	Invitrogen	364-0031-82

Supplementary References

1. Liang, S.H., et al., *Hemizygosity Enables a Mutational Transition Governing Fungal Virulence and Commensalism*. Cell Host Microbe, 2019. **25**(3): p. 418-431 e6.
2. Liang, S.H., et al., *The hyphal-specific toxin candidalysin promotes fungal gut commensalism*. Nature, 2024. **627**(8004): p. 620-627.
