# Supplementary material for: An IL-17-DUOX2 axis controls gastrointestinal colonization by Candida albicans
Source: Nat Commun. 2026 May 4;17:6013. doi: 10.1038/s41467-026-72174-5 (PMC13346697; doi:10.1038/s41467-026-72174-5)
Supplement: Supplementary file 2 — Reporting Summary [file 41467_2026_72174_MOESM2_ESM.pdf]

nature portfolio

Corresponding author(s): Richard Bennett, NCOMMS-24-55290A

Last updated by author(s): Feb 17, 2026

Reporting Summary

Nature Portfolio wishes to improve the reproducibility of the work that we publish. This form provides structure for consistency and transparency in reporting. For further information on Nature Portfolio policies, see our Editorial Policies and the Editorial Policy Checklist.

Statistics

For all statistical analyses, confirm that the following items are present in the figure legend, table legend, main text, or Methods section.

n/a	Confirmed	
☐	☒	The exact sample size (*n*) for each experimental group/condition, given as a discrete number and unit of measurement
☐	☒	A statement on whether measurements were taken from distinct samples or whether the same sample was measured repeatedly
☐	☒	The statistical test(s) used AND whether they are one- or two-sided<br>*Only common tests should be described solely by name; describe more complex techniques in the Methods section.*
☐	☒	A description of all covariates tested
☐	☒	A description of any assumptions or corrections, such as tests of normality and adjustment for multiple comparisons
☐	☒	A full description of the statistical parameters including central tendency (e.g. means) or other basic estimates (e.g. regression coefficient) AND variation (e.g. standard deviation) or associated estimates of uncertainty (e.g. confidence intervals)
☐	☒	For null hypothesis testing, the test statistic (e.g. F, t, r) with confidence intervals, effect sizes, degrees of freedom and P value noted<br>*Give P values as exact values whenever suitable.*
☒	☐	For Bayesian analysis, information on the choice of priors and Markov chain Monte Carlo settings
☒	☐	For hierarchical and complex designs, identification of the appropriate level for tests and full reporting of outcomes
☒	☐	Estimates of effect sizes (e.g. Cohen's *d*, Pearson's *r*), indicating how they were calculated

Our web collection on statistics for biologists contains articles on many of the points above.

Software and code

Policy information about availability of computer code

Data collection	No external software was used for data collection.
Data analysis	Statistical analyses were carried out using GraphPad Prism 10.

For manuscripts utilizing custom algorithms or software that are central to the research but not yet described in published literature, software must be made available to editors and reviewers. We strongly encourage code deposition in a community repository (e.g. GitHub). See the Nature Portfolio guidelines for submitting code & software for further information.

Data

Policy information about availability of data

All manuscripts must include a data availability statement. This statement should provide the following information, where applicable:

- Accession codes, unique identifiers, or web links for publicly available datasets
- A description of any restrictions on data availability
- For clinical datasets or third party data, please ensure that the statement adheres to our policy

Raw data for RNA-seq has been deposited at NCBI with GEO accession number GSE274260. The processed data files for RNA-seq can be found under BioProject PRJNA1145473.

Research involving human participants, their data, or biological material

Policy information about studies with human participants or human data. See also policy information about sex, gender (identity/presentation), and sexual orientation and race, ethnicity and racism.

Reporting on sex and gender	N/A
Reporting on race, ethnicity, or other socially relevant groupings	N/A
Population characteristics	N/A
Recruitment	N/A
Ethics oversight	N/A

Note that full information on the approval of the study protocol must also be provided in the manuscript.

Field-specific reporting

Please select the one below that is the best fit for your research. If you are not sure, read the appropriate sections before making your selection.

[x] Life sciences [ ] Behavioural & social sciences [ ] Ecological, evolutionary & environmental sciences

For a reference copy of the document with all sections, see nature.com/documents/nr-reporting-summary-flat.pdf

Life sciences study design

All studies must disclose on these points even when the disclosure is negative.

Sample size	Sample size of mouse experiment is determined by the feasibility and requirement of statistical analysis. Each experiment group consists of 3 or more mice.
Data exclusions	No data was excluded.
Replication	The findings were verified by using different mouse backgrounds , mouse lines and C. albicans strains, and by multiple research teams in different institutes. Experiments were conducted at least twice independently.
Randomization	Animals were grouped randomly to conduct the experiment.
Blinding	Blinding is not feasible in this study as all animals were included.

Reporting for specific materials, systems and methods

We require information from authors about some types of materials, experimental systems and methods used in many studies. Here, indicate whether each material, system or method listed is relevant to your study. If you are not sure if a list item applies to your research, read the appropriate section before selecting a response.

Materials & experimental systems

n/a	Involved in the study	
[ ]	[x]	Antibodies
[x]	[ ]	Eukaryotic cell lines
[x]	[ ]	Palaeontology and archaeology
[ ]	[x]	Animals and other organisms
[x]	[ ]	Clinical data
[x]	[ ]	Dual use research of concern
[x]	[ ]	Plants

Methods

n/a	Involved in the study	
[x]	[ ]	ChIP-seq
[ ]	[x]	Flow cytometry
[x]	[ ]	MRI-based neuroimaging

Antibodies

Antibodies used	Anti-Candida antibody-Thermo Fisher Scientific-PA173154, Donkey anti-Rabbit IgG (H+L) Cross-Adsorbed Secondary Antibody-DyLight™ 594 ThermoFischer-SA5-10040, Biotin anti-mouse Lineage Panel-BioLegend-133307, Brilliant Violet 785™ anti-mouse CD4 Antibody-BioLegend-100551, CD45 Monoclonal Antibody (2D1), Super Bright™ 645-eBioscience™ -64-9459-42, Brilliant Violet 510™ anti-mouse CD90.2 (Thy-1.2) Antibody-BioLegend-140319, CD335 (NKp46) Monoclonal Antibody (29A1.4), PE, eBioscience ThermoFisher 12-3351-80, Brilliant Violet 711™ anti-T-bet Antibody-BioLegend-644819, Gata-3 Monoclonal Antibody (TWAJ), PerCP-eFluor™ 710, eBioscience™ -46-9966-41, ROR gamma (t) Monoclonal Antibody (B2D), APC, eBioscience™ eBioscience 17-6981-80,

	FOXP3 Monoclonal Antibody (FJK-16s), PE-Cyanine5, eBioscience™ eBioscience 15-5773-80, IL-17A Monoclonal Antibody (eBio17B7), Alexa Fluor 488, eBioscience™ Thermo Scientific 53-7177-81, DUOX2 Antibody-Novus Biologicals-NB110-61576. All the antibodies used for FACS were used at a dilution of 1:100 in the staining buffer.
Validation	The anti-Candida-FITC antibody was validated by the company and the statement is quoted below: "The PA1-73154 antibody reacts with Candida albicans." Donkey anti-Rabbit IgG (H+L) Cross-Adsorbed Secondary Antibody, DyLight™ 594 Anti-Rabbit secondary antibodies are affinity-purified antibodies with well-characterized specificity for rabbit immunoglobulins and are useful in the detection, sorting or purification of its specified target. Biotin anti-mouse Lineage Panel Each lot of these antibodies is quality control tested by immunofluorescent staining with flow cytometric analysis. Brilliant Violet 785™ anti-mouse CD4 Antibody- Each lot of this antibody is quality control tested by immunofluorescent staining with flow cytometric analysis. CD45 Monoclonal Antibody (2D1), Super Bright™ 645, eBioscience™- This Antibody was verified by Knockout to ensure that the antibody binds to the antigen stated. Brilliant Violet 510™ anti-mouse CD90.2 (Thy-1.2) Antibody- Each lot of this antibody is quality control tested by immunofluorescent staining with flow cytometric analysis. CD335 (NKp46) Monoclonal Antibody (29A1.4), PE, eBioscience- This 29A1.4 antibody has been tested by flow cytometric analysis or mouse splenocytes. Brilliant Violet 711™ anti-T-bet Antibody- Each lot of this antibody is quality control tested by intracellular immunofluorescent staining using our True-Nuclear™ Transcription Factor Staining Protocol. Gata-3 Monoclonal Antibody (TWAJ), PerCP-eFluor™ 710, eBioscience™ -This TWAJ antibody has been pre-titrated and tested by intracellular staining and flow cytometric analysis of mouse thymocytes using the Foxp3/Transcription Factor Staining Buffer Set (Product # 00-5523) and protocol. ROR gamma (t) Monoclonal Antibody (B2D), APC, eBioscience™- This B2D antibody has been tested by intracellular staining and flow cytometric analysis using the Foxp3/Transcription Factor Staining Buffer Set (Product # 00-5523-00) and protocol. FOXP3 Monoclonal Antibody (FJK-16s), PE-Cyanine5, eBioscience™- This FJK-16s antibody has been tested by intracellular staining and flow cytometric analysis of mouse splenocytes using the Foxp3/Transcription Factor Buffer Set (Product # 00-5523-00) and protocol. IL-17A Monoclonal Antibody (eBio17B7), Alexa Fluor 488, eBioscience™- This eBio17B7 antibody has been tested by intracellular staining and flow cytometric analysis of restimulated, Th17-polarized mouse splenocytes using the Intracellular Fixation and Permeabilization Buffer Set (Product # 88-8824-00) and protocol. DUOX2 Antibody- Expression of the target protein is compared before and after knockout or knockdown using CRISPR/CAS9 or siRNA/shRNA. If protein expression following knockout or knockdown is substantially reduced, then antibody specificity is ensured. Alexa Fluor® 700 anti-mouse CD45 Antibody-Each lot of this antibody is quality control tested by immunofluorescent staining with flow cytometric analysis. PE/Cyanine7 anti-mouse TCR γ/δ Antibody- Each lot of this antibody is quality control tested by immunofluorescent staining with flow cytometric analysis. CD3e Monoclonal Antibody (145-2C11), Brilliant Ultra Violet™ 496, eBioscience™- This 145-2C11 antibody has been tested by flow cytometric analysis of mouse splenocytes.

Animals and other research organisms

Policy information about studies involving animals; ARRIVE guidelines recommended for reporting animal research, and Sex and Gender in Research

Laboratory animals	C57BL/6J female mice were purchased from Jackson Laboratories and C57BL/6nTac female mice were purchased from Taconic Biosciences. Epithelial knockout line of Duoxa1/a2 was provided by the laboratory of Dr. Jonathan Kaunitz. Experiments with Il17ra-/- mice were carried out in the laboratory of Dr. Sarah Gaffen.
Wild animals	No wild animals were used.
Reporting on sex	The outcomes of C. albicans fitness in the gastrointestinal tracts is independent of animal sex, which has been demonstrated in previous studies. For the experiments with epithelial knockout of Duoxa1/a2, both female and male mice were used.
Field-collected samples	No field-collected samples were used.
Ethics oversight	Animal studies were performed according to approved protocols by the Institutional Animal Care and Use Committee (IACUC) of Brown University and University of Pittsburgh.

Note that full information on the approval of the study protocol must also be provided in the manuscript.

Plants

Seed stocks	N/A
Novel plant genotypes	N/A
Authentication	N/A

Flow Cytometry

Plots

Confirm that:

- [x] The axis labels state the marker and fluorochrome used (e.g. CD4-FITC).
- [x] The axis scales are clearly visible. Include numbers along axes only for bottom left plot of group (a 'group' is an analysis of identical markers).
- [x] All plots are contour plots with outliers or pseudocolor plots.
- [x] A numerical value for number of cells or percentage (with statistics) is provided.

Methodology

Sample preparation	The immune cells were isolated from mouse colon lamina propria of Duoxa1/a2 floxed and Duoxa1/a2 Villin-Cre mice.
Instrument	CytekAurora - 4 Lasers
Software	FlowJo 10.10
Cell population abundance	~500,000 cells isolated from mouse colon lamina propria were gated on CytekAurora machine.
Gating strategy	In FSC/SSC gates, lymphocytes were selected followed by single cell gating with FSC-A/FSC-H and FSC-A/LiveDead gating. CD45+ cells were then gated from Live cell population. For T-cell sorting, cells were gated as CD45+CD4+. These populations were subjected to gate for different subsets of T-cells as Th1-CD45+CD4+Tbet+, Th2-CD45+CD4+GATA3+, Th17-CD45+CD4+RORt+ and Treg-CD45+CD4+FOXP3+. Th17 and Treg cells were further gated for IL-17A+ population as CD45+CD4+RORt+IL-17A+ and Treg-CD45+CD4+FOXP3+ IL-17A+. For the gating of TCR T-cells, CD45+ cells were gated as CD45+ TCR+.<br>To gate for different types of innate lymphoid cells (ILCs), in FSC/SSC gates, lymphocytes were selected followed by single cell gating with FSC-A/FSC-H and FSC-A/LiveDead gating. CD45+ cells were then gated from Live cell population. CD45+ population were gated for Lineage negative (Lin-) population using lineage markers. CD45+Lin- cells were further gated for CD90.2+ cells and then for different subsets of ILCs as ILC1-CD45+Lin-CD90.2+Tbet+, ILC2-CD45+Lin-CD90.2+GATA3+ and ILC3-CD45+Lin-CD90.2+RORt+.

- [x] Tick this box to confirm that a figure exemplifying the gating strategy is provided in the Supplementary Information.
