# Supplementary material for: An IL-17-DUOX2 axis controls gastrointestinal colonization by Candida albicans
Source: Nat Commun. 2026 May 4;17:6013. doi: 10.1038/s41467-026-72174-5 (PMC13346697; doi:10.1038/s41467-026-72174-5)
Supplement: Supplementary file 3 — Transparent Peer Review file [file 41467_2026_72174_MOESM3_ESM.pdf]

nature portfolio

Peer Review File

An IL-17-DUOX2 axis controls gastrointestinal colonization by *Candida albicans*

Corresponding Author: Professor Richard Bennett

This file contains all reviewer reports in order by version, followed by all author rebuttals in order by version.

Version 0:

Reviewer comments:

Reviewer #1

(Remarks to the Author)
Reviewer's response:
The study by Kakade et al. studies Candida colonization in mice under germ-free conditions. They found that IL-17 and Duox2 play both roles in regulating fungal colonization in the gut. While the work has novel elements and most of the data are convincing in supporting this novel mechanism, it needs additional, key experiments to be done to establish a more complete model, and also lacks significant points in the discussion. Please find below my main concerns:
- It is not obvious from the images shown in Figure 2F and I that only the expected morphologies (hyphae/yeasts) of Candida were observed with the mutants, as expected. Could the author perform the same quantitative analysis as in Supplementary figures 1C and 3C?
- What is the relevance or meaning of ece1-induced Duox2/Duoxa2 expression when the toxin does not play a major role in promoting fungal gut colonization?
- The data in figure 4 B-D show that Duox2 deficiency in the gut leads to lower fungal burden. This suggests that Duox2 promotes, does not inhibit, fungal gut colonization in mice. This has not been clearly articulated by the investigators. It leaves the reader somewhat confused since Duox2-derived H2O2 was originally proposed to have antifungal effects in the gut since reactive oxidants can kill fungi. The role of Duox2 here proposed by the authors suggests the opposite, Duox2-derived H2O2 is (somehow) needed to promote Candida gut colonization. Please elaborate on this in the discussion to paint a clearer picture for the reader.
- The mouse strain used to study Duox2 also lacks a functional Duox1/Duoxa1 complex. How do the authors know that the observations made in this mouse strain are due to deficiency in Duox2, not in Duox1? Please discuss this in more detail or provide alternative experiments delivering stronger data for a role of Duox2, not Duox1.
- Duox2 deficiency has long been known in human patients and its main clinical manifestation is problems with thyroid functions. Has anyone measured Candida colonization/infections in Duox2-deficient human patients? If yes, pls cite those papers. If not, please discuss this. Please also discuss briefly what consequences could an enhanced Candida colonization in Duox2-deficient human patients lead to?
- Several of the relevant findings are not relevant in general, to the entire GI tract, and are only specific to one single area of the GI tract. Please discuss this in more detail.
- This study used germ-free mice or mice treated with antibiotics and challenged them with the fungus. Candida established colonization in the mouse gut in the absence of bacteria. The authors should emphasize and justify this model better because in real life bacteria are present in the gut in large quantities.
- The authors should also take a look at RNA levels of Nox2, the core unit of the neutrophil NADPH oxidase, that produces superoxide and subsequent H2O2. As it was mentioned in the paper, IL-17 drives neutrophilic infiltration. Duox1 and Duox2 have also been shown or proposed to promote neutrophil migration into the airway lumen. A similar role could take place in the gut. Duox2 or IL-17 in the experimental model of the manuscript could affect H2O2 production and associated measures of the model. The authors should measure Nox2 transcript levels. If Nox2 levels go higher, Nox2-deficient mice (available at Jackson) should be tested in one of the models
- For the same reason, the authors should consider measuring neutrophil numbers in the gut lumen under the most important experimental conditions.
- The red staining (Duox2) is also present in the gut lumen on the fluorescent image of Candida-infected mice (Figure 2F, ileum). Does that mean Duox2 is present on some granular structures in the gut lumen, as well? Are those shed-off gut epithelial cells? Could that be a nonspecific staining of the Duox2 antibody? Could the authors show images of Candida-infected ileal Duox2 immunofluorescence in the absence of the primary Duox2 antibody to rule out a potential nonspecific

binding of the antibody.

Reviewer #2

(Remarks to the Author)
This manuscript by Bennett and colleagues explores how the antifungal response modulates C. albicans gut colonization. This is a timely and important question in light of the increasing recognition that C. albicans is not only an opportunistic fungal pathogen, but also modulates homeostatic processes and the pathogenesis of diverse non-infectious inflammatory disorders. The authors use the well-established model of gut colonization in germ-free or antibiotic treated wildtype mice. They first conduct a transcriptomic analysis to identify host genes that are differentially regulated in response to C. albicans gut colonization. From the numerous upregulated genes, they choose to follow up Duox2 and Duoxa2 (encoding NADPH oxidase activity). The authors propose that Duox2/Duoxa2 induction depends on the capacity of C. albicans to switch between the yeast and hyphal morphology and on the ECE1 gene, which codes for the virulence factor candidalysin, and that that Duox2/Duoxa2 expression by epithelial cells is indirect and depends on IL-17. Their data further suggest that DUOX2 governs C. albicans colonization and filamentation via IL-17. The detailed molecular relationships underlying causality remain somewhat unclear and merit additional experimental evidence to fully support the claims.

Specific comments:
The causal links between IL-17 production, Duox2/Duoxa2 expression, fungal colonization and fungal morphology remain vague.
(i) Is there a direct effect of reactive oxygen species on fungal colonization and morphology or is the entire effect mediated vie the impact of Duox2/Duoxa2 on the regulation of IL-17?
(ii) How does Duox2/Duoxa2 regulate IL-17 production by the different T cell subsets? Is this specific to the IL-17 induction in response to C. albicans, or a more general regulatory mechanism?
(iii) How are colonization levels and filamentation linked? Increased filamentation in IEC-specific Duoxa1-deficient mice is associated with reduced fungal load, which may be a consequence of the disadvantage of the hyphal morphology compared to yeasts in the gastrointestinal tract of GF/gnotobiotic mice, as several previous studies have shown. However, in IL-17ra knockout mice, the increased proportion of hyphae does not correlate with reduced, but rather increased fungal loads. This needs experimental clarification.
(iv) Likewise, the differences of the role of the IL-17/DUOX2 axis between ileum and colon remain vague.
(v) In line 284 and in the model in Figure 5 the authors refer to an innate source of IL-17 triggering Duox2/Duoxa2 expression. The functional relevance of ILCs upstream of Duox2/Duoxa2 is not provided by just showing that IL-17 production by this cellular subset is not altered in absence of Duox2/Duoxa2. Unless a causal link (e.g. by means of gene deletion/cell depletion experiment), the scheme is to be adjusted.

The authors have demonstrated recently that filamentation and ECE1 expression is an advantage for C. albicans colonization in eubiotic mice, opposed to the situation in GF/gnotobiotic mice. What is the role of the newly identified IL-17/DUOX2 axis in SPF mice, or even in wildling mice, which more closely reflect the situation in naturally colonized hosts?

Fig. 2B: why is the mucus staining (in green) so abundant in the lumen and why is it so patchy? Why does it look so different from what is shown in panels F, I and L, where UEA1 and WGA1 are labelled with a different fluorescent marker.

Figure 2J, 2M: To what extent is the reduced Duox2/Duoxa2 expression in response to the nrg1 deletion mutant (and to a lesser degree to the ece1 deletion mutant) compared to the respective WT control strain of C. albicans a consequence of the reduced fungal load?

For T cell subsets and IL-17 production by different T cell and ILC populations: The y-axis labels are unclear. What do the % refer to? e.g. in Fig. 4D , is this %IL-17+ of CD4+? -> write it explicitly in each case. In addition to showing the % IL-17+, absolute numbers of IL-17+ cell subsets should also be shown. For ILCs, the gating strategy is missing and should be shown.

Display of FACS plots in Fig. 4D-G, Fig. S9A: Please use proper biexponential axes throughout. In several plots, a fraction of the cells is on/below the axis.

Reviewer #3

(Remarks to the Author)
Comments for review of “An IL-17-DUOX2 axis controls gastrointestinal colonization by Candida albicans”

The author’s stated goal is to take a global expression profile approach to understanding host-pathogen crosstalk that regulates C. albicans colonization in the gastrointestinal tract. From their RNAseq analysis, they focus on the Duox/Duoxa2 genes, which are highly expressed in the GI tract but have not been functionally linked to fungal colonization. The authors further identify that C. albicans hyphal-specific factor candidalysin can induce Duox2/Duoxa2. Additionally, IL-17 signaling was shown to induce Duox2/Duoxa2 expression. While IL-17 signaling suppressed C. albicans colonization at early time points (day 7), Duox/Duoxa2 promotes colonization at later time points (day 17-28).

An understanding of molecular interactions that govern fungal colonization in host niches is needed. The authors’ study seeks to address this knowledge gap by investigating the role of a dual oxidase system in regulating C. albicans

colonization in the intestinal tract. Multiple mouse models are used to demonstrate the induction of these genes by C. albicans, which contributes to the robustness of the study. Additionally, the manuscript is well written, and results are explained clearly throughout. However, additional experiments or rephrasing some of the text to discuss the timing discrepancy of the data would strengthen the manuscript and bolster the conclusions drawn. Specific comments are detailed below:

Major comments:

The authors describe a system by which C. albicans induces IL-17, which drives Duox2/Duoxa2 expression in the gut to modulate C. albicans colonization. However, the effects of IL-17 signaling (in Figure 3) are evaluated at 7 days post inoculation, and the effects of Duox2/Duoxa2 (in Figure 4) are evaluated at 28 days post inoculation. So while both of these factors are shown to play a role, it is at distinct time points during Candida colonization. Do the IL-17ra mice have defects in Duox2/duox2a induction at this later time point? Does candidalysin still contribute to Duox2/duox2a gene expression at 28 dpi? The temporal differences examined could also be expanded upon in the discussion (such as with lines 362-364), or made more clear in the introduction and throughout the results to set up that temporal difference in the role of IL-17-Duox axis.

Fig 3 - The authors hypothesize that IL-17 is induced by C. albicans (linked to candidalysin production) and that this is driving Duox2/Duoxa2 expression in the GI tract. However, IL-17 levels were not measured in these tissues- are IL-17 levels elevated during colonization at these time points? Does the Ece1 mutant fail to induce IL-17 in this niche, or can supplementation of IL-17 rescue the defect of Ece1 in inducing Duox2/Duoxa2?

Minor comments:

Line 100 – Why are the ileum and colon specifically chosen for RNA-seq analysis over other tissues?

Fig 1E – The GSEA analysis pathways presented as a bar graph with the FDR q-value isn't particularly informative. Perhaps the data could be plotted with a normalized expression score? The upregulated and downregulated genes could be combined on the same graph.

182-184 – Alternatively, the lack of Duox2/Duoxa2 expression in tissues from mice inoculated with the nrg1 mutant may be due to the poor colonization of this mutant, with ~100 fold less fungi present in the colon vs. WT C. albicans. So, there may be too few fungi to induce Duox2/Duoxa2.

Line 206-207 & Supplementary Figure 5 – Could direct interaction with live C. albicans cells be required? Would the presence of candidalysin (either purified or comparing an ece1 mutant) induce hydrogen peroxide production? Also, are Duox2/Duoxa2 being induced when stimulated with these different cell wall components, or could there be another source of hydrogen peroxide in the colonoids?

Lines 222-238 – The logical flow of the story could be enhanced by moving this section to after the section in lines 239-248.

Line 229/230 & Supplementary Figure 6B – The significance bars are missing on the graph.

337-339 – A little more explanation of this reference is needed it is not clear to understand how the CD11c phagocyte profiling demonstrates hyphal-independent host responses.

368-369 – The impacts to IgA levels were not shown in the figures.

Fig 5 - How does candidalysin expression fit into this model?

Version 1:

Reviewer comments:

Reviewer #1

(Remarks to the Author)
I thank the authors for responding to my critiques. The revised manuscript has carefully addressed all my concerns and questions by either discussing it in more detail in the article or by performing new experiments and delivering new results.

Reviewer #2

(Remarks to the Author)
The authors have addressed my comments and generated new data to support their claims about the contribution of different lymphocyte subset to IL-17 production. They also provide additional data with IL-17RA-deficient mice to provide a more solid correlation between DUOX and the IL-17 response. They adjusted the text for more transparency regarding the unresolved open questions about the mechanism by which the different components are linked (fungal load, filamentation, direct effect of H_2O_2 on the fungus, etc).

The authors have however not addressed my question as to whether the DUOX-dependent change in IL-17 induction was C. albicans-specific. They don't show how T cell IL-17 production is affected by the absence of epithelial DUOX in non-colonized animals, and they do not selectively assess C. albicans-responsive T cells by re-stimulating T cells with the fungus (instead of PMA/ionomycin).

The following statements in the discussion need to be tuned down as the authors do not provide direct experimental evidence (irrespective of what they put in the limitations sections):
- The statement in line 360 ("… the latter an important reactive oxygen species (ROS) in GI homeostasis")   the authors do not provide experimental evidence for H2O2 playing an important role in vivo in GI homeostasis.
- Line 415: "…and elevates H2O2 levels in the gut."   the authors show H2O2 production by colonoids, but not in the gut in vivo.
Line 416: "DUOX2 and H2O2 levels remain elevated which leads to a reduction in IL-17A."   the authors do not quantify H2O2 levels in the gut over time.

A few additional concerns, mainly related to the newly added data, need to be addressed by the authors:
Figure 3D-E: y-axis labels are unclear. When showing the proportion of IL-17+ cells within a cell population, the y-axis label should read "% IL-17+ of …" The same applies in other figures showing % of cell populations.
Suppl. Figure S6 (and S14): Gating strategy: The separation of lineage+ and lineage- cells is far from convincing, given the bad separation between the two populations and the diagonal shape of the "lineage+" population. Which antibodies were included to determine 'lineage'?? This information should be included in the methods section and/or legends and not only in the reporting summary! If 'lineage' contains several different antibodies that bind distinct cells differing in size and expression levels of the antibody-bound molecules, it is hardly possible that the lineage+ population is so condensed and uniform as it appears in the CD45-AF700 vs. lin-APC-eFluor780 flow plot. If the lineage cocktail contains anti-CD3ε, anti-Ly-6G/Ly-6C, anti-CD11b, anti-CD45R/B220, anti-Ter-119 (according to the information in the reporting summary) it is also hardly possible that the lineage+ population makes up only ~50% of all CD45+ cells. In Supplementary Figure 14, the gating on lineage-negative cells was done differently gate on lineage-negative prior to gating on CD45+. Why this inconsistency? The size of the lin-CD45+ population does not appear more reliable in the latter figure.
Gating on CD45+ TCRgd+ to identify gdT cells is minimalistic. Including CD3 or CD90 in the gating strategy would much improve resolution of the population for accurate quantification.
Please also show isotype or FMO controls for 'lineage', TCRgd, Tbet, GATA3, RORgt and IL-17 stainings in Suppl. Figures S6, S11, S13

Supplementary Fig. S10C: what are the y-axis units in the panel to the right?

Supplementary Fig. S13 and S14 (panels at the bottom): the gates for the IL-17+ subsets of Foxp3+ and RORgt+ T cells are misplaced. Please include isotype or FMO controls for IL-17 to correctly determine the boundary between the IL-17+ vs. IL-17- subsets. The quantification graphs need to be adjusted after adjusting the gates.

The title of Supplementary Figures S12, S13 and S14 should be adjusted, if Duoxa1/a2ΔIEC mice are compared to Duoxa1/a2fl/fl mice. The latter are not WT mice.

Reviewer #3

(Remarks to the Author)
I appreciate the authors' attention to addressing each of the major and minor comments for the manuscript. My concerns have been sufficiently addressed with the revisions.

Version 2:

Reviewer comments:

Reviewer #2

(Remarks to the Author)
The authors have made significant efforts to address my comments.

Open Access This Peer Review File is licensed under a Creative Commons Attribution 4.0 International License, which permits use, sharing, adaptation, distribution and reproduction in any medium or format, as long as you give appropriate credit to the original author(s) and the source, provide a link to the Creative Commons license, and indicate if changes were made.
In cases where reviewers are anonymous, credit should be given to 'Anonymous Referee' and the source.
The images or other third party material in this Peer Review File are included in the article's Creative Commons license, unless indicated otherwise in a credit line to the material. If material is not included in the article's Creative Commons license and your intended use is not permitted by statutory regulation or exceeds the permitted use, you will need to obtain permission directly from the copyright holder.
To view a copy of this license, visit https://creativecommons.org/licenses/by/4.0/

We would like to thank the three reviewers for their comments on the paper and for the suggestion of further experiments to strengthen the overall work. We have taken the necessary time to perform several additional experiments that are now included in the revised manuscript. We hope that the extensive changes made to the paper now meet with the expectations of the reviewers. The response to each reviewer comment is highlighted in blue below.

Reviewer #1 (Remarks to the Author):

Reviewer's response:
The study by Kakade et al. studies Candida colonization in mice under germ-free conditions. They found that IL-17 and Duox2 play both roles in regulating fungal colonization in the gut. While the work has novel elements and most of the data are convincing in supporting this novel mechanism, it needs additional, key experiments to be done to establish a more complete model, and also lacks significant points in the discussion. Please find below my main concerns:
- It is not obvious from the images shown in Figure 2F and I that only the expected morphologies (hyphae/yeasts) of Candida were observed with the mutants, as expected. Could the author perform the same quantitative analysis as in Supplementary figures 1C and 3C?

- We thank the reviewer for highlighting this point and we have carried out quantification of yeast and hyphal morphotypes from WT, *efg1* and *nrg1* colonized mice. These data have been added as Supplementary Figure 3I and 4C.

- What is the relevance or meaning of ece1-induced Duox2/Duoxa2 expression when the toxin does not play a major role in promoting fungal gut colonization?

- We agree with the reviewer that this is a relevant point and note that the Ece1-generated toxin does play a major role in promoting gut colonization but that this effect is most relevant in the present of bacteria (when competition for growth in the gut niche is at its highest; Liang et al., 2024, PMID: 38448595). We emphasize that we are interested in the mechanisms directing fungal-host interactions, including the factors downstream of Efg1 and Nrg1 as cells lacking either factor show defects in Duox2/Duoxa2 induction. We have edited the text to better put these results into context.

- The data in figure 4 B-D show that Duox2 deficiency in the gut leads to lower fungal burden. This suggests that Duox2 promotes, does not inhibit, fungal gut colonization in mice. This has not been clearly articulated by the investigators. It leaves the reader somewhat confused since Duox2-derived H2O2 was originally proposed to have antifungal effects in the gut since reactive oxidants can kill fungi. The role of Duox2 here proposed by the authors suggests the opposite, Duox2-derived H2O2 is (somehow)

needed to promote Candida gut colonization. Please elaborate on this in the discussion to paint a clearer picture for the reader.

- We agree that our results are counterintuitive as we also expected for Duox2 to inhibit *C. albicans* colonization, and this touches on the complexities of microbe-host interactions. To address this, we have further edited the Discussion to make this clearer.

- The mouse strain used to study Duox2 also lacks a functional Duox1/Duoxa1 complex. How do the authors know that the observations made in this mouse strain are due to deficiency in Duox2, not in Duox1? Please discuss this in more detail or provide alternative experiments delivering stronger data for a role of Duox2, not Duox1.

- This is a valid point and we note that Duox1 is expressed at very low levels in the mouse GI tract in comparison to Duox2 (Grasberger et al, Gastroenterology, 2015). Moreover, we did not observe any changes in the expression level of Duox1 in the ileum and colon tissues of mice colonized with *C. albicans* (Supplementary Figure 3E-F). The epithelial knockout of Duoxa1 and a2 (Duoxa1/a2$^{\Delta IEC}$) is a well-accepted model to study the functional role of Duox2 and has been used in other studies including Burgueño et al, Frontiers in Physiology, 2019, Burgueño et al, Gastroenterology, 2021, Hazime et al, Cellular and Molecular Gastroenterology and Hepatology, Hazime et al, Inflammatory Bowel Diseases, 2025. We have further edited the text to make this point clearer.

- Duox2 deficiency has long been known in human patients and its main clinical manifestation is problems with thyroid functions. Has anyone measured Candida colonization/infections in Duox2-deficient human patients? If yes, pls cite those papers. If not, please discuss this. Please also discuss briefly what consequences could an enhanced Candida colonization in Duox2-deficient human patients lead to?

- We are not aware of any study that has examined Candida colonization or disease in Duox2-deficient patients. We used a Villin-Cre regulated gut specific knockout of Duoxa1/a2 which results in absence of functional Duox2 only in the gastrointestinal tract of mice. Text has been added to this effect.

- Several of the relevant findings are not relevant in general, to the entire GI tract, and are only specific to one single area of the GI tract. Please discuss this in more detail.

- We have added the text to highlight that our work has focused on the small and large intestinal regions of the GI tract.

- This study used germ-free mice or mice treated with antibiotics and challenged them with the fungus. Candida established colonization in the mouse gut in the absence of

bacteria. The authors should emphasize and justify this model better because in real life bacteria are present in the gut in large quantities.

- The reviewer is correct that our study deliberately utilized germ-free mice to initially investigate *Candida*-specific changes in the host. This approach was chosen so that the response to *Candida* could be investigated without confounding changes in the bacterial microbiome. However, we then validated several of the key findings in conventional mice treated with antibiotics. These approaches were taken to obtain the higher levels of *Candida* colonization necessary to see responses in the host and are the standard approaches used in the field to study *Candida*-gut interactions. Moreover, we have also examined *Candida* colonization in mice without antibiotics for comparison and this data is now included as Supplementary Figure 11.

- The authors should also take a look at RNA levels of Nox2, the core unit of the neutrophil NADPH oxidase, that produces superoxide and subsequent H2O2. As it was mentioned in the paper, IL-17 drives neutrophilic infiltration. Duox1 and Duox2 have also been shown or proposed to promote neutrophil migration into the airway lumen. A similar role could take place in the gut. Duox2 or IL-17 in the experimental model of the manuscript could affect H2O2 production and associated measures of the model. The authors should measure Nox2 transcript levels. If Nox2 levels go higher, Nox2-deficient mice (available at Jackson) should be tested in one of the models.

- This is an excellent suggestion, and we carried out gene expression analyses of the NADPH oxidases Duox1, Nox1 and Nox2 in control vs *C. albicans*-colonized mice. However, we did not observe significant changes in the expression of these genes as shown in Supplementary Figure 3E and 3F.

- For the same reason, the authors should consider measuring neutrophil numbers in the gut lumen under the most important experimental conditions.

- As we did not observe any *C. albicans*-mediated changes in Nox2 expression levels we did not assess neutrophil infiltration in our model.

- The red staining (Duox2) is also present in the gut lumen on the fluorescent image of Candida-infected mice (Figure 2F, ileum). Does that mean Duox2 is present on some granular structures in the gut lumen, as well? Are those shed-off gut epithelial cells? Could that be a nonspecific staining of the Duox2 antibody? Could the authors show images of Candida-infected ileal Duox2 immunofluorescence in the absence of the primary Duox2 antibody to rule out a potential nonspecific binding of the antibody.

- We carried out the staining of colon sections from *C. albicans*-colonized mice with

only the secondary antibody and observed that the antibody shows some non-specific binding to gut luminal material (Supplementary Fig 2H).

Reviewer #2 (Remarks to the Author):

This manuscript by Bennett and colleagues explores how the antifungal response modulates C. albicans gut colonization. This is a timely and important question in light of the increasing recognition that C. albicans is not only an opportunistic fungal pathogen, but also modulates homeostatic processes and the pathogenesis of diverse non-infectious inflammatory disorders. The authors use the well-established model of gut colonization in germ-free or antibiotic treated wildtype mice. They first conduct a transcriptomic analysis to identify host genes that are differentially regulated in response to C. albicans gut colonization. From the numerous upregulated genes, they choose to follow up Duox2 and Duoxa2 (encoding NADPH oxidase activity). The authors propose that Duox2/Duoxa2 induction depends on the capacity of C. albicans to switch between the yeast and hyphal morphology and on the ECE1 gene, which codes for the virulence factor candidalysin, and that that Duox2/Duoxa2 expression by epithelial cells is indirect and depends on IL-17. Their data further suggest that DUOX2 governs C. albicans colonization and filamentation via IL-17. The detailed molecular relationships underlying causality remain somewhat unclear and merit additional experimental evidence to fully support the claims.

- We thank the reviewer for their positive comments on the goals of the study and have addressed the additional comments below.

Specific comments:
The causal links between IL-17 production, Duox2/Duoxa2 expression, fungal colonization and fungal morphology remain vague.
(i) Is there a direct effect of reactive oxygen species on fungal colonization and morphology or is the entire effect mediated vie the impact of Duox2/Duoxa2 on the regulation of IL-17?

- We agree with the reviewer that it can be very challenging to make direct connections between these factors given the complexity of the GI environment, and that measuring reactive oxygen species in this niche is particularly demanding due to their short half-lives. We have edited the text to highlight where questions in the model remain but note that colonization levels decrease in mice lacking Duox2 and it is therefore likely that the effect of Duox2 is primarily mediated through the IL-17-DUOX2 axis rather than via ROS. In terms of morphology, H_2O_2 has been shown to inhibit the growth but promote the hyphal formation of *C. albicans* cells in vitro (Nasution et al, Eukaryotic cell, 2008). Given that we observe more hyphae in the Duox2-deficient mice this is therefore unlikely to be a response to a change in ROS.

(ii) How does Duox2/Duoxa2 regulate IL-17 production by the different T cell subsets? Is this specific to the IL-17 induction in response to C. albicans, or a more general regulatory mechanism?

- We agree with the reviewer that the connection between Duox2/Duoxa2 and IL-17 production by T cell subsets is an important one. We examined T cell populations and did not observe any significant changes in the differentiation of T cells into Th17 or Treg subsets upon *C. albicans* colonization.
- In our model, we demonstrate that *C. albicans* colonization triggers upregulation of Duox2 resulting in increased H_2O_2 levels which in turn enhances the production of IL-17A, primarily from Th17 cells and, to a lesser extent, from Treg cells. These findings suggest that the DUOX2-dependent increase in IL-17A production is specific to *C. albicans* colonization and is not due to altered T-cell differentiation. However, the precise mechanisms by which DUOX2/DUOXA2 modulates IL-17A expression across different T-cell subsets remain unclear and warrant further investigation. Elucidating these pathways lies beyond the scope of the current study.

(iii) How are colonization levels and filamentation linked? Increased filamentation in IEC-specific Duoxa1-deficient mice is associated with reduced fungal load, which may be a consequence of the disadvantage of the hyphal morphology compared to yeasts in the gastrointestinal tract of GF/gnotobiotic mice, as several previous studies have shown. However, in IL-17ra knockout mice, the increased proportion of hyphae does not correlate with reduced, but rather increased fungal loads. This needs experimental clarification.

- We now note that the connection between colonization levels and filamentation is a more complex one than first envisaged, as a simple relationship between the degree of filamentation and colonization levels is not observed across all experiments. We initially examined *C. albicans* colonization of Duox2-deficient mice for 4 weeks while the study with *Il17ra*$^{-}$/$^{-}$ mice was performed only for 7 days. To enable a direct comparison of colonization and filamentation phenotypes between these two models, we extended the *C. albicans* colonization in *Il17ra*$^{-}$/$^{-}$ mice to 4 weeks. Interestingly, *C. albicans* showed increased colonization in *Il17ra*$^{-}$/$^{-}$ mice on day 28 compared to the wild-type (WT) mice controls. Notably, however, we also observed increased filamentation in the *Il17ra*$^{-}$/$^{-}$ mice compared to WT mice at day 28. These findings reveal that, in antibiotic-treated hosts, increased filamentation does not necessarily correlate with reduced fungal colonization of the gut. This new experiment has been added as Figure 4 and the role of filamentation more carefully outlined in the Discussion.

(iv) Likewise, the differences of the role of the IL-17/DUOX2 axis between ileum and colon remain vague.

- We note that we observed increased expression of Duox2/Duoxa2 in both the ileum and the colon, with generally higher induction observed in the colon. We have therefore carried out most of our analyses in both the tissues and have added additional text to highlight this point.

(v) In line 284 and in the model in Figure 5 the authors refer to an innate source of IL-17 triggering Duox2/Duoxa2 expression. The functional relevance of ILCs upstream of Duox2/Duoxa2 is not provided by just showing that IL-17 production by this cellular subset is not altered in absence of Duox2/Duoxa2. Unless a causal link (e.g. by means of gene deletion/cell depletion experiment), the scheme is to be adjusted.

- To address this point, we carried out an additional experiment in which mice were colonized with *C. albicans* WT or *ece1Δ/Δ* cells for 7 days. Our data show that ILC3 and TCR $\gamma\delta$ T cells exhibited enhanced production of IL-17A in the presence of WT *C. albicans* cells but not with *ece1* cells that lack the candidalysin toxin (Figure 3D-E). We have updated the model to include these results.

The authors have demonstrated recently that filamentation and ECE1 expression is an advantage for C. albicans colonization in eubiotic mice, opposed to the situation in GF/gnotobiotic mice. What is the role of the newly identified IL-17/DUOX2 axis in SPF mice, or even in wildling mice, which more closely reflect the situation in naturally colonized hosts?

- The contribution of IL-17/DUOX2 to *C. albicans* colonization in eubiotic mice is a very valid question. We therefore examined *C. albicans* colonization in WT and Duox2-deficient mice under conditions without antibiotic intervention, using both standard murine chow and a purified murine diet (that promotes more stable *C. albicans* colonization). In both eubiotic models, we observed no significant differences in *C. albicans* colonization levels between Duox2-deficient and WT mice (Supplementary Figure 15). Thus, the IL-17/Duox2 axis does not influence overall levels of fungal colonization in these models.

Fig. 2B: why is the mucus staining (in green) so abundant in the lumen and why is it so patchy? Why does it look so different from what is shown in panels F, I and L, where UEA1 and WGA1 are labelled with a different fluorescent marker.

- We thank the reviewer for noting this and have replaced these images with better images.

Figure 2J, 2M: To what extent is the reduced Duox2/Duoxa2 expression in response to the nrg1 deletion mutant (and to a lesser degree to the ece1 deletion mutant) compared

to the respective WT control strain of *C. albicans* a consequence of the reduced fungal load?

- It is difficult to know exactly to what extent the reduced Duox2/Duoxa2 expression in the nrg1-colonized mice is simply the result of the lower colonization levels. However, given that the colonization levels of nrg1 cells are 50-100-fold lower than those with WT cells we expect that this will be a substantial part of the reason for the lower response, and this is now stated more clearly in the text (and in the new Discussion section describing the limitations of the study).

For T cell subsets and IL-17 production by different T cell and ILC populations: The y-axis labels are unclear. What do the % refer to? e.g. in Fig. 4D, is this %IL-17+ of CD4+? -> write it explicitly in each case. In addition to showing the % IL-17+, absolute numbers of IL-17+ cell subsets should also be shown. For ILCs, the gating strategy is missing and should be shown.

- We thank the reviewer for these points. These edits have been made and the gating strategy for ILCs is included as a Supplementary Figure 14.

Display of FACS plots in Fig. 4D-G, Fig. S9A: Please use proper biexponential axes throughout. In several plots, a fraction of the cells is on/below the axis.

- Edits have been made as suggested.

Reviewer #3 (Remarks to the Author):

Comments for review of “An IL-17-DUOX2 axis controls gastrointestinal colonization by Candida albicans”

The author’s stated goal is to take a global expression profile approach to understanding host-pathogen crosstalk that regulates C. albicans colonization in the gastrointestinal tract. From their RNAseq analysis, they focus on the Duox/Duoxa2 genes, which are highly expressed in the GI tract but have not been functionally linked to fungal colonization. The authors further identify that C. albicans hyphal-specific factor candidalysin can induce Duox2/Duoxa2. Additionally, IL-17 signaling was shown to induce Duox2/Duoxa2 expression. While IL-17 signaling suppressed C. albicans colonization at early time points (day 7), Duox/Duoxa2 promotes colonization at later time points (day 17-28).

An understanding of molecular interactions that govern fungal colonization in host

niches is needed. The authors' study seeks to address this knowledge gap by investigating the role of a dual oxidase system in regulating C. albicans colonization in the intestinal tract. Multiple mouse models are used to demonstrate the induction of these genes by C. albicans, which contributes to the robustness of the study. Additionally, the manuscript is well written, and results are explained clearly throughout. However, additional experiments or rephrasing some of the text to discuss the timing discrepancy of the data would strengthen the manuscript and bolster the conclusions drawn. Specific comments are detailed below:

- We thank Reviewer 3 for their positive feedback on the manuscript while also highlighting several important points that we discuss below.

Major comments:

The authors describe a system by which C. albicans induces IL-17, which drives Duox2/Duoxa2 expression in the gut to modulate C. albicans colonization. However, the effects of IL-17 signaling (in Figure 3) are evaluated at 7 days post inoculation, and the effects of Duox2/Duoxa2 (in Figure 4) are evaluated at 28 days post inoculation. So while both of these factors are shown to play a role, it is at distinct time points during Candida colonization. Do the IL-17ra mice have defects in Duox2/duox2a induction at this later time point? Does candidalysin still contribute to Duox2/duox2a gene expression at 28 dpi? The temporal differences examined could also be expanded upon in the discussion (such as with lines 362-364), or made more clear in the introduction and throughout the results to set up that temporal difference in the role of IL-17-Duox axis.

- We agree with the reviewer that this is a critical question and therefore conducted an additional experiment in which *C. albicans* colonization was performed in *Il17ra*$^{-}$/$^{-}$ mice for 4 weeks to directly compare colonization to that in the DUOX2-defective mice at this time point. Consistent with our observations at day 7, *Il17ra*$^{-}$/$^{-}$ mice showed increased fungal colonization (and increased filamentation) at 28 days relative to WT controls. These findings highlight that loss of IL-17 signaling leads to increased fungal levels at both early and late stages of colonization. This data has been added as part of the new Figure 4.
- We also evaluated the levels of *Duox2*/*Duoxa2* expression at day 28 in these mice. We observed decreased expression of these genes in the colon of *Il17ra* $^{-/-}$ mice relative to WT mice (with no difference in the ileum tissues). This text has been added to the Results.
- We further note that our RNA-seq analyses were performed on germ-free mice colonized with *C. albicans* for 7 and 21 days and that we observed greater transcriptional changes at day 7 than at day 21. Duox2 and Duoxa2 showed robust upregulation at day 7 but only moderate upregulation at day 21 (Figure 1, Supplementary Figure 3). Based on these findings, we believe that further analysis of host gene expression at later time points (day 21 or day 28) is unlikely to yield

additional insights. We have added the appropriate text in the discussion to clarify this point.

Fig 3 - The authors hypothesize that IL-17 is induced by C. albicans (linked to candidalysin production) and that this is driving Duox2/Duoxa2 expression in the GI tract. However, IL-17 levels were not measured in these tissues- are IL-17 levels elevated during colonization at these time points? Does the Ece1 mutant fail to induce IL-17 in this niche, or can supplementation of IL-17 rescue the defect of Ece1 in inducing Duox2/Duoxa2?

- We agree that the induction of IL-17 is an important point and we therefore carried out an additional experiment in which mice were colonized by WT or *ece1* C. albicans cells for 7 days and analysis of IL-17A producing cells performed. We found that ILC3 and $\gamma\delta$ T-cells showed an enhanced production of IL-17 in WT cells but that this was lacking from mice colonized with ece1 mutant cells (Figure 3C-D). Based on this data appropriate changes have been made in the model.

Minor comments:

Line 100 – Why are the ileum and colon specifically chosen for RNA-seq analysis over other tissues?

- We have examined *C. albicans* colonization in each part of the murine gastrointestinal tract and found increasing fungal cells in descending the GI from the duodenum to the colon. For these reasons, the RNA-seq analysis was performed on tissues from the distal ileum and colon. To clarify this, appropriate text has been added in the result section.

Fig 1E – The GSEA analysis pathways presented as a bar graph with the FDR q-value isn't particularly informative. Perhaps the data could be plotted with a normalized expression score? The upregulated and downregulated genes could be combined on the same graph.

- The requested edits have been made in Figure 1E.

182-184 – Alternatively, the lack of Duox2/Duoxa2 expression in tissues from mice inoculated with the nrg1 mutant may be due to the poor colonization of this mutant, with ~100 fold less fungi present in the colon vs. WT C. albicans. So, there may be too few fungi to induce Duox2/Duoxa2.

- It is challenging to determine the extent to which reduced colonization by nrg1 cells has impacted Duox2/Duoxa2 expression. However, since nrg1 colonization levels are 50-100-fold lower than those observed with WT cells, this likely contributes substantially to the reduced response. This point is now more clearly addressed in the main text and in the limitations section of the Discussion.

Line 206-207 & Supplementary Figure 5 – Could direct interaction with live C. albicans cells be required? Would the presence of candidalysin (either purified or comparing an ece1 mutant) induce hydrogen peroxide production? Also, are Duox2/Duoxa2 being induced when stimulated with these different cell wall components, or could there be another source of hydrogen peroxide in the colonoids?

- We tried adding live *Candida* cells to colonoids but even at low doses, the fungal cells caused extensive damage to the colonoids, preventing analysis of H_2O_2 production or host gene expression. To overcome this limitation, we also tested heat-killed *C. albicans* cells (both yeast and hyphal forms) as well as various fungal cell wall components to assess their impact on H_2O_2 production. However, none of these conditions led to an increase in H_2O_2 levels. We therefore did not proceed further with the analysis of *Duox2*/*Duoxa2* gene expression in this experimental setup.
- Although other NADPH oxidases such as *Duox1* and *Nox2* are expressed at low levels in colonic epithelial cells, *C. albicans* colonization did not increase their expression. Moreover, colonoids generated from mice defective in Duox1/Duox2 failed to produce H_2O_2. Our data is therefore consistent with DUOX2 being responsible for H_2O_2 production in colonoids in response to *C. albicans*.

Lines 222-238 – The logical flow of the story could be enhanced by moving this section to after the section in lines 239-248.

- We have made the appropriate changes to the text.

Line 229/230 & Supplementary Figure 6B – The significance bars are missing on the graph.

- Errors bars have been added to this figure as requested.

337-339 – A little more explanation of this reference is needed it is not clear to understand how the CD11c phagocyte profiling demonstrates hyphal-independent host responses.

- We have edited the text to clarify this point.

368-369 – The impacts to IgA levels were not shown in the figures.

- Total IgA and Candida-bound IgA levels have now been examined and this data has been added as a Supplementary Figure 10E.

Fig 5 - How does candidalysin expression fit into this model?

- Additional experiments show that *C. albicans* candidalysin promotes innate IL-17A production from ILC3 and $\gamma\delta$ T-cells which in turn induces expression of

Duox2/*Duoxa2*. At the later time points of colonization (post day 14), functional *Duox2* represses the synthesis of IL-17A from Th17 and Treg cells leading to increased colonization of *C. albicans*. We have made these edits in the model to clarify these points (Figure 6).

We would like to thank the three reviewers for their comments and are appreciative that reviewers 1 and 3 now find the revised version of the paper acceptable for publication in its current form. We respond to the additional comments from reviewer 2 below.

REVIEWER COMMENTS

Reviewer #2 (Remarks to the Author):

The authors have addressed my comments and generated new data to support their claims about the contribution of different lymphocyte subset to IL-17 production. They also provide additional data with IL-17RA-deficient mice to provide a more solid correlation between DUOX and the IL-17 response. They adjusted the text for more transparency regarding the unresolved open questions about the mechanism by which the different components are linked (fungal load, filamentation, direct effect of H_2O_2 on the fungus, etc).

We thank the reviewer for noting how the suggested changes to the paper have strengthened the overall manuscript.

The authors have however not addressed my question as to whether the DUOX-dependent change in IL-17 induction was C. albicans-specific. They don't show how T cell IL-17 production is affected by the absence of epithelial DUOX in non-colonized animals, and they do not selectively assess C. albicans-responsive T cells by re-stimulating T cells with the fungus (instead of PMA/ionomycin).

While we agree that these are interesting lines of questioning, we did not address whether the DUOX2-dependent change in IL-17 production was C. albicans specific in this study. We chose to focus on the role of DUOX2 in C. albicans colonized mice as that was the target of our investigation. Unfortunately, it is not feasible for us to repeat the experiments in WT versus DUOX2-deficient mice +/- C. albicans colonization as we no longer have a breeding colony of the DUOX2 KO mice.

The following statements in the discussion need to be tuned down as the authors do not provide direct experimental evidence (irrespective of what they put in the limitations sections):
- The statement in line 360 ("… the latter an important reactive oxygen species (ROS) in GI homeostasis") ◊ the authors do not provide experimental evidence for H_2O_2 playing an important role in vivo in GI homeostasis.

This sentence in the discussion section has been modified.

- Line 415: "…and elevates H_2O_2 levels in the gut." ◊ the authors show H_2O_2 production by colonoids, but not in the gut in vivo.

We have edited this sentence.

Line 416: "DUOX2 and H_2O_2 levels remain elevated which leads to a reduction in IL-17A." ◊ the authors do not quantify H_2O_2 levels in the gut over time.

We have made edits to this sentence.

A few additional concerns, mainly related to the newly added data, need to be addressed by the authors:
Figure 3D-E: y-axis labels are unclear. When showing the proportion of IL-17+ cells within a cell population, the y-axis label should read "% IL-17+ of …" The same applies in other figures showing % of cell populations.

The y-axis labels in the figures showing FACS data has been edited as per the Reviewer's suggestion.

Suppl. Figure S6 (and S14): Gating strategy: The separation of lineage+ and lineage- cells is far from convincing, given the bad separation between the two populations and the diagonal shape of the "lineage+" population. Which antibodies were included to determine 'lineage'?? This information should be included in the methods section and/or legends and not only in the reporting summary! If 'lineage' contains several different antibodies that bind distinct cells differing in size and expression levels of the antibody-bound molecules, it is hardly possible that the lineage+ population is so condensed and uniform as it appears in the CD45-AF700 vs. lin-APC-eFluor780 flow plot. If the lineage cocktail contains anti-CD3ε, anti-Ly-6G/Ly-6C, anti-CD11b, anti-CD45R/B220, anti-Ter-119 (according to the information in the reporting summary) it is also hardly possible that the lineage+ population makes up only ~50% of all CD45+ cells.

We thank the reviewer for this comment and their careful analysis of the gating. The lineage cocktail included biotinylated anti-CD3ε, anti-Ly-6G/Ly-6C, anti-CD11b, anti-CD45R/B220 and anti-Ter-119 antibodies and this this information is now included in the methods section. After more careful analysis we noted that there is an intermediate population between the lineage positive and the negative population which is tending towards the positive population. We have omitted this intermediate population from the lineage negative gate and reanalyzed the data. This data is now provided as Figure 3D-E and Supplementary Fig. 6A-C.

In Supplementary Figure 14, the gating on lineage-negative cells was done differently gate on lineage-negative prior to gating on CD45+. Why this inconsistency? The size of the lin-CD45+ population does not appear more reliable in the latter figure.

This data has been re-analyzed and the CD45+ population is gated first followed by the lineage negative population. This data is in Supplementary figure 14-16.

Gating on CD45+ TCRgd+ to identify gdT cells is minimalistic. Including CD3 or CD90 in the gating strategy would much improve resolution of the population for accurate quantification.

As per the reviewer's suggestion, we repeated the experiment to get better resolution of early immune responses and included the CD3 marker in addition to other surface markers. We analyzed the data by gating on the CD45+CD3+ population followed by gating for TCRgd+ and IL-17A+ cells.

Please also show isotype or FMO controls for 'lineage', TCRgd, Tbet, GATA3, RORgt and IL-17 stainings in Suppl. Figures S6, S11, S13.

Additional controls for these experiments have been included in the revision in Supplementary Figure 7 and 8 as requested.

Supplementary Fig. S10C: what are the y-axis units in the panel to the right?

Units have been added to the y-axis in the right panel of this figure.

Supplementary Fig. S13 and S14 (panels at the bottom): the gates for the IL-17+ subsets of Foxp3+ and RORgt+ T cells are misplaced. Please include isotype or FMO controls for IL-17 to correctly determine the boundary between the IL-17+ vs. IL-17- subsets. The quantification graphs need to be adjusted after adjusting the gates.

We thank the reviewer for noting this and the gating has been corrected for the bottom panels of Supplementary Figures 13-14 which are now Supplementary Figures 15-16. The quantification has also been reevaluated accordingly (Supplementary Figure 14).

The title of Supplementary Figures S12, S13 and S14 should be adjusted, if Duoxa1/a2ΔIEC mice are compared to Duoxa1/a2fl/fl mice. The latter are not WT mice.

These edits have been made.
